# Synthesis and Crystallographic Insight into the Structural Aspects of Some Novel Adamantane-Based Ester Derivatives

**DOI:** 10.3390/molecules201018827

**Published:** 2015-10-16

**Authors:** C. S. Chidan Kumar, Huey Chong Kwong, Siau Hui Mah, Tze Shyang Chia, Wan-Sin Loh, Ching Kheng Quah, Gin Keat Lim, Siddegowda Chandraju, Hoong-Kun Fun

**Affiliations:** 1 X-ray Crystallography Unit, School of Physics, Universiti Sains Malaysia, Penang 11800, Malaysia; E-Mails: chiatzeshyang@hotmail.com (T.S.C.); wansin_loh@live.com (W.-S.L.); hkfun@usm.my (H.-K.F.); 2 Department of Chemistry, Alva’s Institute of Engineering & Technology, Visvesvaraya Technological University, Mijar, Moodbidri 574225, India; 3 School of Chemical Sciences, Universiti Sains Malaysia, Penang 11800, Malaysia; E-Mails: stonekg01@gmail.com (H.C.K.); limgk@usm.my (G.K.L.); 4 School of Biosciences, Taylor’s University, Lakeside Campus, Subang Jaya 47500, Selangor, Malaysia; E-Mail: siauhui.mah@taylors.edu.my; 5 Department of Sugar Technology & Chemistry, University of Mysore, Sir M.V. PG Center, Mandya 571402, Karnataka, India; E-Mail: chandraju1@yahoo.com; 6 Department of Pharmaceutical Chemistry, College of Pharmacy, King Saud University, Riyadh 11451, Saudi Arabia

**Keywords:** adamantyl, synclinal, crystal packing, antioxidant, anti-inflammatory

## Abstract

Adamantyl-based compounds are commercially important in the treatments for neurological conditions and type-2 diabetes, aside from their anti-viral abilities. Their values in drug design are chronicled as multi-dimensional. In the present study, a series of 2-(adamantan-1-yl)-2-oxoethyl benzoates, **2**(**a**–**q**), and 2-(adamantan-1-yl)-2-oxoethyl 2-pyridinecarboxylate, **2r**, were synthesized by reacting 1-adamantyl bromomethyl ketone with various carboxylic acids using potassium carbonate in dimethylformamide medium at room temperature. Three-dimensional structures studied using X-ray diffraction suggest that the adamantyl moiety can serve as an efficient building block to synthesize 2-oxopropyl benzoate derivatives with synclinal conformation with a looser-packed crystal packing system. Compounds **2a**, **2b**, **2f**, **2g**, **2i**, **2j**, **2m**, **2n**, **2o**, **2q** and **2r** exhibit strong antioxidant activities in the hydrogen peroxide radical scavenging test. Furthermore, three compounds, **2p**, **2q** and **2r**, show good anti-inflammatory activities in the evaluation of albumin denaturation.

## 1. Introduction

Adamantane is the simplest diamondoid with a chemical formula of C_10_H_16_. It consists of four cyclohexane rings in an armchair configuration. To date, seven adamantane derivatives have been applied in clinical usage to treat acne vulgaris [1], Alzheimer’s disease [2], as an anti-viral [3,4,5,6,7] and for type-2 diabetes [8,9,10], while the others are in development as potential therapeutics for iron overload disease, neurological conditions, malaria, tuberculosis and cancers [11]. Previous studies on adamantyl-based compounds revealed their antioxidant and anti-inflammatory properties [12,13,14]. As a reactive oxygen species, hydrogen peroxide is generated as a by-product of biological reaction. These reactive species will cause oxidative damaging effects in living cells. Hydrogen peroxide acts as a weak oxidizing agent and reacts with Fe^2+^ or Cu^2+^ ions to form the hydroxyl radical [15]. Similarly, free radicals, such as DPPH (2,2-diphenyl-1-picrylhydrazyl), are readily attacked and promote oxidative damage of biomolecules, such as lipids, proteins and DNA, leading to many human pathological conditions [16,17]. On the other hand, failure in the downregulation of pro-inflammatory mediators will result in an imbalance between inflammation and its regulation, which leads to chronic inflammation due to excessive macrophage responses. The resulting disease conditions from chronic inflammation include asthma, rheumatic arthritis, atherosclerosis and cancer [18]. Protection against protein denaturation, a well-documented phenomenon caused by the inflammation process, is the main consideration in developing non-steroidal anti-inflammatory drugs (NSAIDs) [19].

Encouraged by the biological activities of adamantane derivatives, we herein present FTIR, ^1^H-NMR and ^13^C-NMR spectra and single-crystal X-ray diffraction analysis of some new adamantyl-based ester derivatives. Using a group comparison of single-crystal X-ray diffraction data, the structural conformation, structural occupancy and crystal packing similarity were studied. Their antioxidant activities were evaluated by hydrogen peroxide and the DPPH radical scavenging assay. In addition, the anti-inflammatory activity of these adamantyl-based ester derivatives was determined by the protein denaturation assay.

## 2. Results and Discussion

**Scheme 1:**
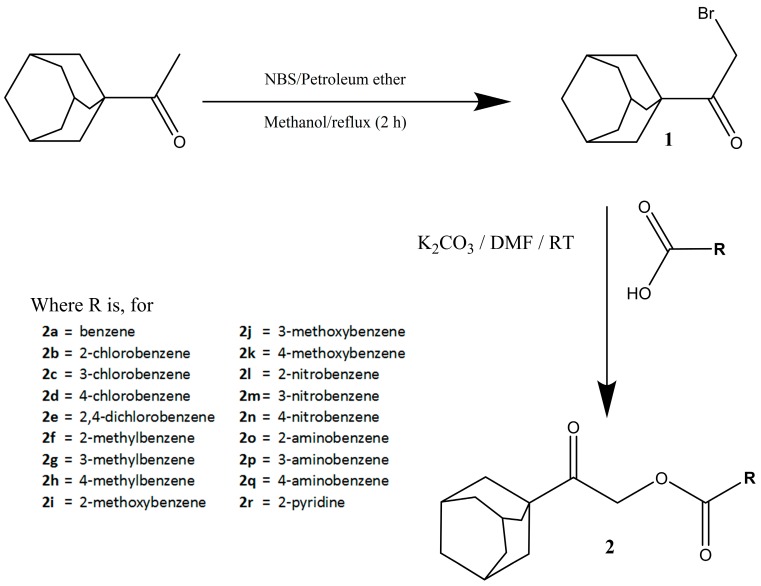
Reaction scheme for **2**(**a**–**r**).

The full reaction involved the synthesis of 1-adamantyl bromomethyl ketone, **1**, which is essential for subsequent synthesis of **2**(**a**–**r**) in accordance with the reported procedure [20], as shown in Scheme 1. The structures of the intermediate and final compounds were elucidated by various spectral techniques, like FTIR, ^1^H-NMR, ^13^C-NMR and single-crystal X-ray diffraction analysis. The asymmetric and symmetric stretchings of the methyl group (asym 2960 cm^−1^: sym 2870 cm^−1^) in **1** had changed to methylene stretchings (asym 2920 cm^−1^: sym 2850 cm^−1^); however, the synthesis of **1** is confirmed by ^1^H-NMR when the peak corresponding to the methyl group of 1-adamantyl methyl ketone is replaced by the peak corresponding to the methylene group near δ 4.1 ppm. Esterification of **2**(**a**–**r**) was confirmed by FTIR, where the peak corresponding to the hydroxyl of carboxylic acid disappeared. Furthermore, some peaks were observed at ~1300 cm^−1^–1020 cm^−1^, which corresponded to C—O stretching of ester. Similarly, the ^1^H-NMR of **2**(**a**–**r**) displays a singlet corresponding to the methylene group at higher chemical shifts (δ 5.1 ppm) associated with the other required aromatic peaks. In the FTIR spectra of **2**(**a**–**r**), the exceptional and predictable observations are the occurrences of N=O stretching in **2**(**l**–**m**) and the N—H stretching in **2**(**o**–**q**) at ~1530 cm^−1^ and ~3490 cm^−1^, respectively; whereas for ^13^C-NMR, the constant appearances of carbonyl carbons peaks at 207 and 165 ppm further support the formation of the ester bond in Compounds **2**(**a**–**r**). The crystal structure for all compounds, except **2m** and **2q**, were determined by single-crystal X-ray diffraction analysis. All spectra, crystallography data and crystal packing are described in detail in the Supplementary Data.

### 2.1. General Description of the Crystal Structure Conformations

The asymmetric unit (*Z*′) of the studied compounds consists of either one (**2c**, **2d**, **2g**, **2h**, **2j**, **2k**, **2n**–**p** and **2r**) or two (**2a**, **2b**, **2e**, **2f**, **2i** and **2l**) crystallographic independent molecules. For a crystal with *Z*′ = 2, the independent molecules are hereafter denoted as Molecules *A* and *B*, respectively. The orientational disorder of the adamantane group is observed in **2a**, **2b**, **2f**, **2j**, **2l** and **2o** with a refined site occupancy ratio, as summarized in the table below (Table 1). All disordered components of adamantane are rotationally-related, and in particular, two-fold rotation disorder (*ca*. 180°) is observed in **2a** (Molecule *B*) and **2f** (Molecule *B*).

**Table 1:** Refined site occupancy ratio for disordered compounds.

Compound	Refined Site Occupancy Ratio
**2a**	0.390 (10):0.610 (10) (*A*)
	0.695 (4):0.305(4) (*B*)
**2b**	0.431 (10):0.569 (10) (*A*)
**2f**	0.716 (6):0.284 (6) (*A*)
	0.793 (4):0.207 (4) (*B*)
**2j**	0.753 (3):0.247 (3)
**2l**	0.873 (4):0.127 (4) (*B*)
**2o**	0.897 (4):0.103 (4)

**Figure 1:**
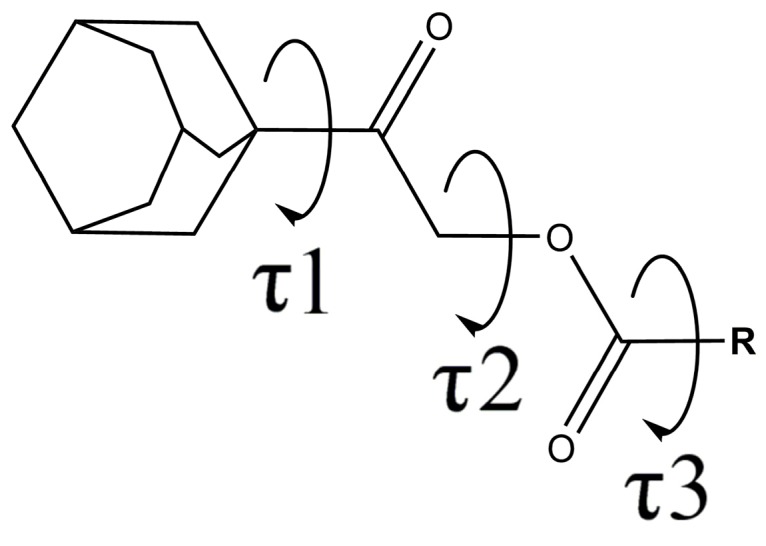
General chemical scheme for all compounds, showing τ1, τ2 and τ3 as torsion angles.

Molecular conformation of these compounds can be characterized by three degrees of freedom, which are the torsion angles of C1—C8—C11—O2 (τ1), C11—C12—O1—C13 (τ2) and O1—C13—C14—C15 (τ3) (Figure 1). Although the torsion angle between the adamantane moiety and the adjacent carbonyl group (C1—C8—C11—O2, τ1) is one of the degrees of freedom, it was irrelevant for the comparison due to its randomness and the effect of disordered orientations. By referring to the previous report [20], torsion angle τ2, which interconnects two carbonyl groups, tends to adopt two types of conformations, either synclinal or periplanar. However, all structures reported in this study adopt only one conformation in which τ2 torsion angles are all in synclinal conformation ranging from 70°–86°, which may be due to the introduction of the bulky adamantane moiety. The range of the O1—C13—C14—C15 τ3 torsion angle, which defines the twisted angle between the carboxylate group and the six-membered ring, is relatively larger compared to τ2, ranging from 1.25°–39.08° and 126.4°–179.57°. In compounds **2e** (2,4-dichlorobenzene), **2i** (2-methoxybenzene) and **2l** (2-nitrobenzene), the τ3 is probably induced by the steric repulsion between the *o*-substituent and the adjacent carbonyl oxygen atom. Nevertheless, exceptional cases are observed in **2b** (2-chlorobenzene), **2f** (2-methylbenzene) and **2o** (2-aminobenzene) with small deviations of 3.78°, 11.55°, 6.64°, 9.58° and 8.08° from perfect planarity. The amino substituent at the -*ortho* position (**2o**) forms a strong intramolecular N—H···O hydrogen bond with the adjacent carbonyl group to improve the planarity of the benzoate group (τ3 = 171.92°). Torsion angles between two carbonyl group (C11—C12—O1—C13, τ2) and torsion angles between the carbonyl group with its adjacent benzene ring (O1—C13—C14—C15, τ3) of the reported compounds are summarized in Table 2.

**Table 2:** Summary of the C11—C12—O1—C13 and O1—C13—C14—C15 torsion angles.

Compound	Substituent	Torsion Angles C11—C12—O1—C13, τ2	Torsion Angles O1—C13—C14—C15, τ3
**2a**	Benzene	−81.31, 75.84	−9.95, 2.84
**2b**	2-Chlorobenzene	78.96, 75.23	11.55, −3.78
**2c**	3-Chlorobenzene	73.10	3.22
**2d**	4-Chlorobenzene	73.75	−179.57
**2e**	2,4-Dichlorobenzene	−69.7, −85.50	138.13, 135.26
**2f**	2-Methylbenzene	−76.09, 77.77	−9.58, 6.64
**2g**	3-Methylbenzene	−72.91	1.25
**2h**	4-Methylbenzene	75.42	−170.83
**2i**	2-Methoxybenzene	−86.12, −79.34	39.08, 156.87
**2j**	3-Methoxybenzene	−75.05	−173.58
**2k**	4-Methoxybenzene	−76.60	168.36
**2l**	2-Nitrobenzene	−70.96, −70.23	126.4, 130.00
**2n**	4-Nitrobenzene	73.57	−1.86
**2o**	2-Aminobenzene	−76.5	171.92
**2p**	3-Aminobenzene	77.13	−17.86
**2r**	2-Pyridine	−75.07	−17.79, 22.32

### 2.2. Structural Occupancy and Crystal Packing Similarity

**Figure 2:**
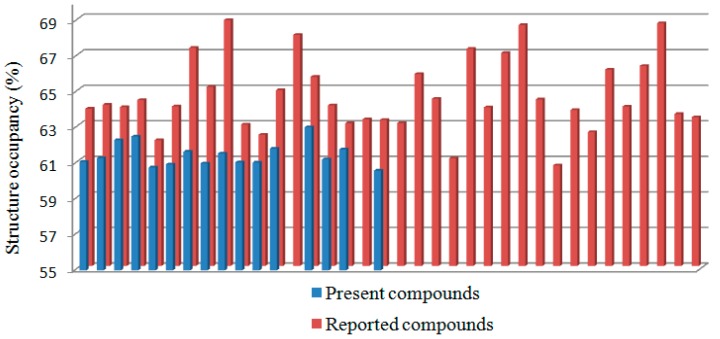
Structural occupancy comparison of the present and reported compounds.

Sixteen present adamantyl-based ester derivatives and thirty-six reported phenacyl benzoate derivatives, which are found from the Cambridge Structure Database (CSD 5.35) search, are compared to each other in order to identify the effect of the replacement of the electron-rich phenyl ring (search result) with the bulky adamantane moiety (present compounds) or *vice versa* on the molecular conformation and structural occupancy. In the aspect of molecular conformation, it is noteworthy to observe that neither periplanar conformation nor mixed (periplanar and synclinal) conformation is adopted by the currently studied compounds, unlike the variations displayed in phenacyl benzoates. All present compounds adopt synclinal conformation with C13—O1—C12—C11 torsion angles falling in the range from 69.7°–86.12°, which is comparable to those previously reported for phenacyl benzoate derivatives that adopt the same conformation (71°–91°) [20]. The structural occupancies of all present and reported compounds are listed in Table 3. The structural occupancy of phenacyl benzoate derivatives has a larger range compared to adamantyl-based ester derivatives and is mostly in between 63% and 69% (30 out of 36). All of the present adamantyl-based compounds are below this range, except **2n** (Figure 2). The existence of the adamantane moiety in the present compounds not only reduces the weak intermolecular π…π or C—H…π interactions as compared to phenacyl benzoates, it also limits the packing patterns of adamantyl-based ester derivatives as indicated by the high occurrence of isostructures.

**Table 3:** List of the structural occupancy of the present and reported compounds ^i^.

Compound	Packing Coefficient (%)	Compound	Packing Coefficient (%)	Compound	Packing Coefficient (%)
**2a**	61.11	**CIXVUC** [20]	63.94	**GITHUN** [21]	64.40
**2b**	61.32	**CIXWAJ** [20]	64.33	**MANGIR** [22]	61.06
**2c**	62.33	**CIXWEN** [20]	62.08	**OBOYIP** [23]	67.22
**2d**	62.53	**CIXWIR** [20]	63.98	**OCAKUA** [24]	63.92
**2e**	60.79	**CIYCAQ** [20]	67.27	**OCAQUG** [25]	66.98
**2f**	60.97	**CIYCEU** [20]	65.07	**OCEFEJ** [26]	68.55
**2g**	61.68	**CIYCIY** [20]	68.83	**PECZAA** [27]	64.37
**2h**	61.02	**CIYCOE** [20]	62.96	**PODQIK** [28]	60.66
**2i**	61.58	**CIYFUN** [20]	62.38	**PODRAD** [29]	63.77
**2j**	61.08	**CIYGAU** [20]	64.89	**USIWID** [30]	62.53
**2k**	61.07	**EVAFOX** [31]	68.00	**USIWOJ** [32]	66.04
**2l**	61.85	**EVAJAN** [33]	65.64	**VOBYUI** [34]	63.97
**2n**	63.05	**EVAJIV** [35]	64.03	**YAFWEJ** [36]	66.25
**2o**	61.24	**EVAZEH** [37]	63.03	**YAFZAI** [38]	68.65
**2p**	61.81	**EVEGIW** [39]	63.25	**YAHGUL** [40]	63.55
**2r**	60.61	**EVEGOC** [41]	63.22	**YAHYOX** [42]	63.37
**AZULUD** [43]	63.85	**EVEVEH** [44]	63.04		
**CIQNEW** [45]	64.07	**GARCEJ** [46]	65.80		

^i^ Each reported compound is represented by the Cambridge Crystallographic Data Centre (CCDC) reference code, and its systematic name is provided in the Supplementary Materials.

**Figure 3:**
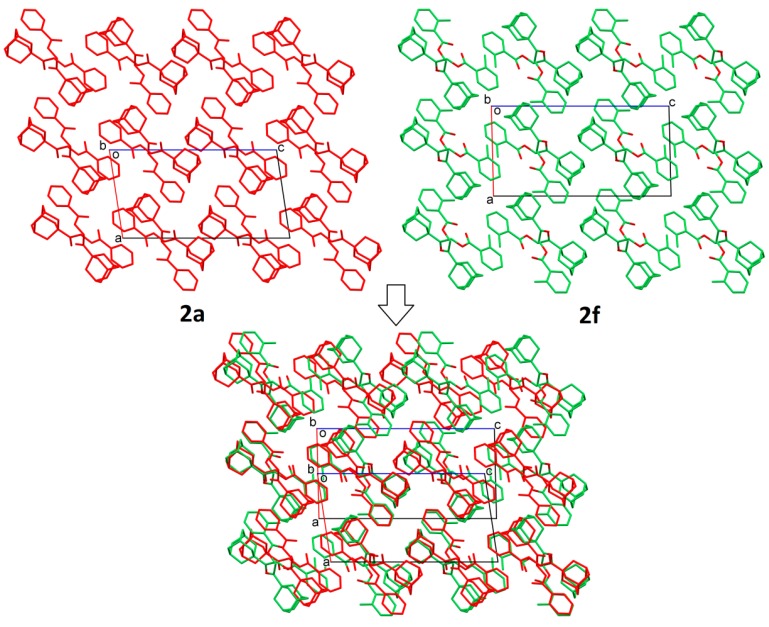
Crystal packing comparison of Compounds **2a** and **2f**.

**Figure 4:**
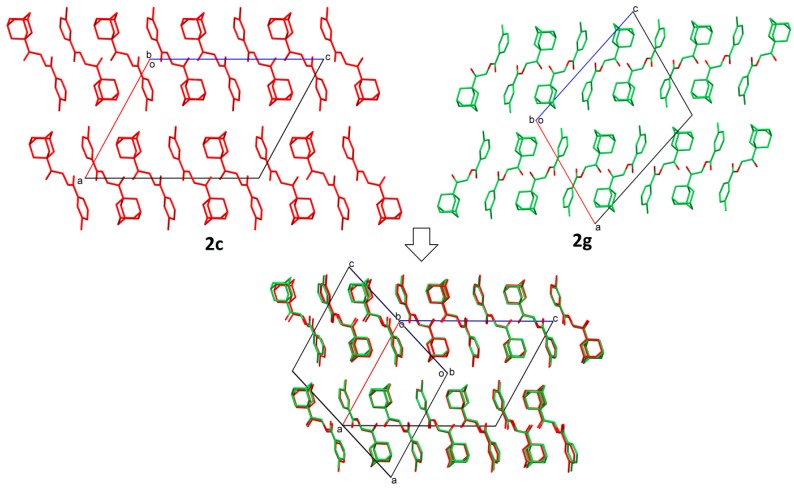
Crystal packing comparison of Compounds **2c** and **2g**.

**Figure 5:**
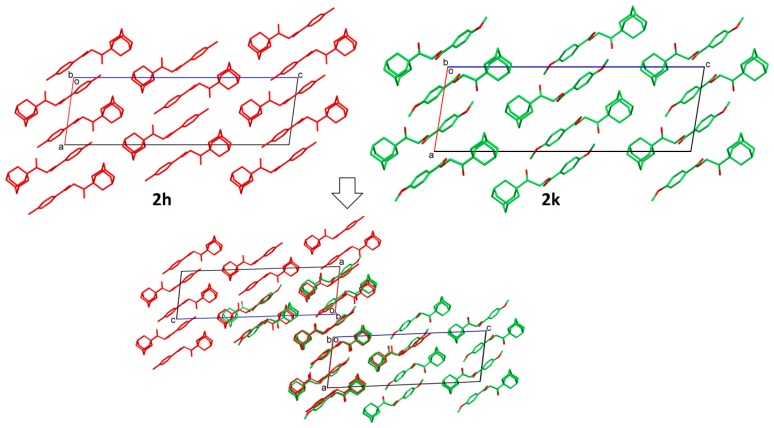
Crystal packing comparison of Compounds **2h** and **2k**.

**Figure 6:**
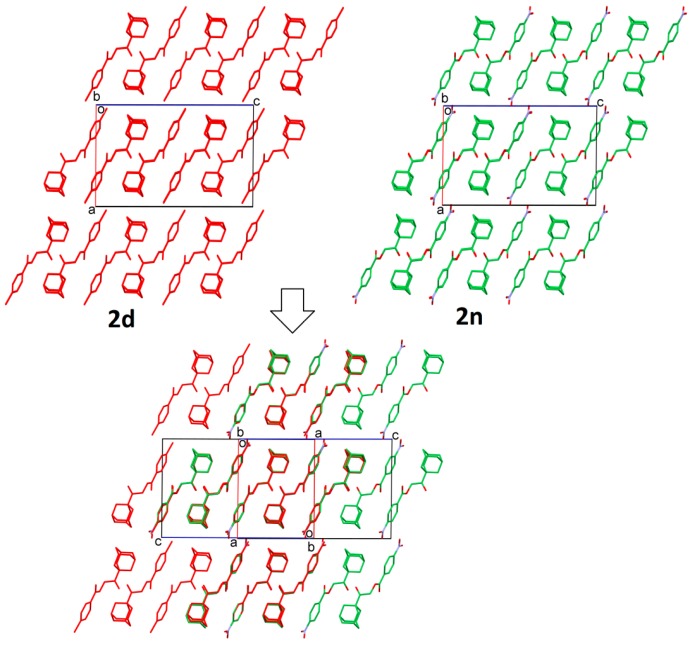
Crystal packing comparison of Compounds **2d** and **2n**.

**Figure 7:**
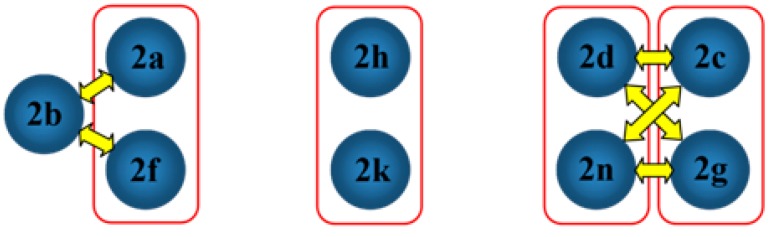
Crystal packing relationship in some studied compounds. Red boxes and yellow arrows indicate 3D and 2D similarities, respectively.

In the investigation of the crystal structural similarity among the current compounds, there are four pairs of compounds (**2a**/**2f**, **2c**/**2g**, **2h**/**2k** and **2d**/**2n**) found, and each pair is crystallized in the same space group with similar lattice constants, which are two main characteristics of 3D structural similarity. Indeed, these four pairs of compounds show isostructural relationships with a similar packing pattern, as shown in their overlaid crystal structure diagrams (Figure 3, Figure 4, Figure 5 and Figure 6, respectively). Besides that, 2D similarities are observed in **2b/2a** and **2b**/**2f**, while Compounds **2c**, **2d**, **2g** and **2n** show 2D similarity among each other (Figure 7).

### 2.3. Antioxidant and Anti-Inflammatory Properties

**Figure 8:**
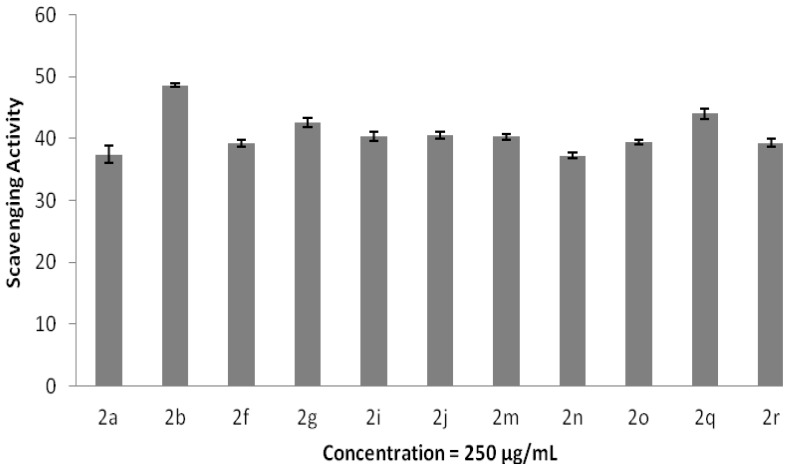
Hydrogen peroxide radical scavenging. The data represent the percentage of hydrogen peroxide radical inhibition (mean ± SD), and experiments were performed in triplicate.

The antioxidant properties of adamantane-based compounds were determined by two methods, which are hydrogen peroxide and DPPH radicals scavenging abilities. The scavenging effects of hydrogen peroxide by the adamantyl-based compounds were evaluated at a concentration of 250 μg/mL and are summarized in Figure 8. Eleven adamantane derivatives show positive scavenging effects on H_2_O_2_. Compound **2b** possesses the strongest scavenging activity, which is 48.55%, followed by Compounds **2q** and **2g** with 42.96% and 42.56%, respectively. The scavenging activities of these compounds are comparable to the standard compound, ascorbic acid, with an inhibition percentage of 43.38%. On the other hand, adamantyl-based compounds did not show positive DPPH radical scavenging properties at a concentration of 1000 μg/mL. The results show that adamantane derivatives are selective to the inhibition of hydrogen peroxide radicals.

**Figure 9:**
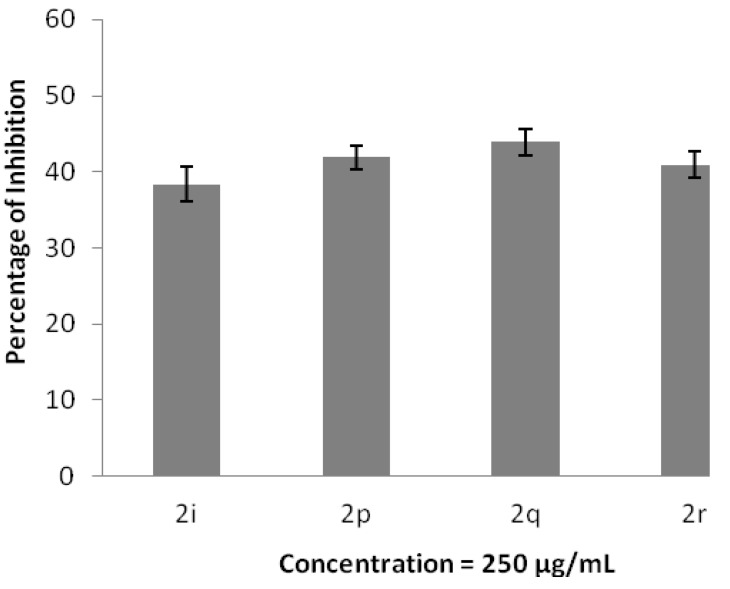
Inhibition of protein denaturation. The data represent the percentage of hydrogen peroxide radical inhibition (mean ± SD), and experiments were performed in triplicate.

The anti-inflammatory effects of adamantyl-based compounds at a concentration of 250 μg/mL were evaluated by the protein denaturation assay. The results of the percentage of inhibition against protein denaturation are presented in Figure 9. Compounds **2p**, **2q** and **2r** are strong protein denaturation inhibitors with inhibition percentages of 41.92%, 43.94% and 40.91%, respectively, which are better than the standard drug, diclofenac sodium, with an inhibition percentage of 37.88%. These three compounds consist of either an amino-substituted phenyl ring or a pyridine ring. Hence, the high inhibition effects are deduced to be the direct contribution from the nitrogen-containing group in adamantyl-based compounds.

## 3. Experimental Section

The reagents and solvents for the synthesis work were obtained commercially from Sigma Aldrich Corporation (St. Louis, MO, USA) and used without any additional purification. Melting points were determined on a Stuart (Staffordshire, UK) SMP10 apparatus. ^1^H and ^13^C nuclear magnetic resonance (NMR) spectra were determined in CDCl_3_ at 500 MHz and 125 MHz, respectively, using a Bruker Advance III 500 spectrometer (Bruker Corporation, Billerica, MA, USA). Fourier transform infrared spectroscopy (FTIR) spectra were recorded on a Perkin Elmer Frontier FTIR spectrometer (PerkinElmer, Inc., Waltham, MA, USA) equipped with attenuated total reflection (ATR).

The X-ray analysis for all samples was performed using a Bruker APEX II DUO CCD diffractometer (Bruker Corporation) employing MoKα radiation (λ = 0.71073 Å) with φ and ω scans. X-ray data for all compounds were collected at room temperature. Data reduction and absorption correction were performed using the SAINT and SADABS program [47]. All structures were solved by the direct method and refined by full-matrix least-squares techniques on *F^2^* using the SHELXTL software package [48]. All non-hydrogen atoms were refined anisotropically, except for the minor disordered components of **2l** and **2o** with a site occupancy of less than 0.2. The C-bound H atoms were calculated geometrically with isotropic displacement parameters set to 1.2-times the equivalent isotropic *U* value of the parent carbon atoms. N-bound H atoms are located by the difference Fourier map and refined freely (N—H = 0.77 (5)—0.87 (6) Å). A similar geometry restraint (SAME) was applied to the disordered adamantane moiety in **2f** and **2o** and also the full molecule in **2r**. Packing coefficient (%)/structure occupancy was calculated using the Olex2 program with the command (-calcvoid) [49]. Crystallographic data for the structures reported in this paper (**2a**–**r**, excluding **2m** and **2q**) have been deposited with the Cambridge Crystallographic Data Centre (CCDC) as supplementary publication. CCDC 1030854–1030869 contain the supplementary crystallographic data for this paper. These data can be obtained free of charge via http://www.ccdc.cam.ac.uk/conts/retrieving.html (or from the CCDC, 12 Union Road, Cambridge CB2 1EZ, UK; Fax: +44 1223 336033; E-mail: deposit@ccdc.cam.ac.uk).

### 3.1. Synthesis

1-Adamantyl methyl ketone was refluxed with *N*-bromo succinimide and petroleum ether in methanol at 333 K for two hours. The resultant 1-adamantyl bromomethyl ketone (**1**) precipitate was filtered and recrystallized with ethanol. After that, 1-adamantyl bromomethyl ketone (**1**) (0.51 g, 0.002 mol) was reacted with the corresponding carboxylic acid (0.003 mol) with the presence of potassium carbonate in DMF (8 mL) and stirred at room temperature for about 3 h. The reaction progress was monitored by thin layer chromatography (TLC). After the reaction completed, the reaction mixture was poured into ice-cooled water and kept stirring for 10 min. The solid obtained was filtered out, washed successively with distilled water and recrystallized from acetone after it dried [20]. All targeted compounds were synthesized in good yield and high purity. Suitable single-crystal specimens were obtained from various types of solvents, as described below. The chemical structures were characterized by using FTIR and NMR spectroscopies. The crystal structures for all compounds except **2m** and **2q** were determined by single-crystal X-ray diffraction analysis.

### 3.2. Spectroscopic Details

*1-Adamantyl bromomethyl ketone* (**1**): Solvent for growing crystal: ethanol; yield: 85%; M.P. 326–328 K; FTIR (ATR (solid) cm^−1^): 2905, 2851 (C–H, ν), 1709 (C=O, ν), 1254, 1094, 1063 (C–O, ν), 733 (C–Cl, ν); ^1^H-NMR (500 MHz, CDCl_3_): δ ppm 4.18 (s, 2H, -CH_2_), 2.09 (br-s, 3H, adamantane-CH), 1.91–1.90 (br-d, 6H, adamantane-CH_2_), 1.80–1.72 (br-q, 6H, adamantane-CH_2_); ^13^C-NMR (125 MHz, CDCl_3_): δ ppm 205.57 (C=O), 46.62 (-CH_2_), 38.53, 36.34, 31.83, 27.83 (adamantane-Cs).

*2-(Adamantan-1-yl)-2-oxoethyl benzoate* (**2a**): Solvent for growing crystal: acetone; yield: 80%; M.P. 374–376 K; FTIR (ATR (solid) cm^−1^): 2917, 2850 (C–H, ν), 1709 (C=O, ν), 1602, 1413 (Ar, C=C, ν), 1277, 1120 (C–O, ν), 706 (C–H, ω); ^1^H-NMR (500 MHz, CDCl_3_): δ ppm 8.10–8.08 (d, 2H, *J* = 7.5 Hz, Ar), 7.58–7.55 (t, 1H, *J* = 7.5 Hz, Ar), 7.46–7.42 (t, 2H, *J* = 7.5 Hz, Ar), 5.10 (s, 2H, -CH_2_), 2.08(br-s, 3H, adamantane-CH), 1.94–1.93 (br-d, 6H, adamantane-CH_2_), 1.80–1.72 (br-q, 6H, adamantane-CH_2_); ^13^C-NMR (125 MHz, CDCl_3_): δ ppm 207.16 (C=O), 166.04 (O-C=O), 133.19, 129.87, 129.58, 128.37 (Ar), 64.94 (-CH_2_), 45.39, 38.02, 36.47, 27.79 (adamantane-Cs).

*2-(Adamantan-1-yl)-2-oxoethyl 2-chlorobenzoate* (**2b**): Solvent for growing crystal: acetone and ethanol (1: 1 *v*/*v*); yield: 83%; M.P. 348–350 K; FTIR (ATR (solid) cm^−1^): 2904, 2850 (C–H, ν), 1709 (C=O, ν), 1590, 1416 (Ar, C=C, ν), 1247, 1024 (C–O, ν), 742 (C–Cl, ν); ^1^H-NMR (500 MHz, CDCl_3_): δ ppm 8.01–8.00 (d, 1H, *J* = 8.0 Hz, Ar), 7.47–7.43 (q, 2H, *J* = 8.0 Hz, Ar), 7.35–7.32 (t, 1H, *J* = 8.0 Hz, Ar), 5.12 (s, 2H, -CH_2_), 2.09 (br-s, 3H, adamantane-CH), 1.93–1.93 (br-d, 6H, adamantane-CH_2_), 1.80–1.72 (br-q, 6H, adamantane-CH_2_); ^13^C-NMR (125 MHz, CDCl_3_): δ ppm 206.98 (C=O), 164.89 (O-C=O), 133.98, 132.84, 131.95, 131.03, 129.35, 126.62 (Ar), 65.18(-CH_2_), 45.38 37.99, 36.44, 27.75 (adamantane-Cs).

*2-(Adamantan-1-yl)-2-oxoethyl 3-chlorobenzoate* (**2c**): Solvent for growing crystal: acetone, ethanol and acetonitrile (1:1:1 *v*/*v*/*v*); yield: 73%; M.P. 403–405 K; FTIR (ATR (solid) cm^−1^): 2911, 2850 (C–H, ν), 1718 (C=O, ν), 1571, 1442 (Ar, C=C, ν), 1295, 1253, 1130 (C–O, ν), 745 (C–Cl, ν); ^1^H-NMR (500 MHz, CDCl_3_): δ ppm 8.07 (s, 1H, Ar), 7.98–7.96 (d, 1H, *J* = 8.0 Hz, Ar), 7.55–7.54 (d, 1H, *J* = 8.0 Hz, Ar), 7.41–7.38 (t, 1H, *J* = 8.0 Hz, Ar), 5.11 (s, 2H, -CH_2_), 2.09 (br-s, 3H, adamantane-CH), 1.93–1.93 (br-d, 6H, adamantane-CH_2_), 1.800–1.719 (br-q, 6H, adamantane-CH_2_); ^13^C-NMR (125 MHz, CDCl_3_): δ ppm 206.94 (C=O), 164.88 (O-C=O), 134.56, 133.27, 131.27, 129.95, 129.73, 128.01 (Ar), 65.23(-CH_2_), 45.37, 37.98, 36.43, 27.74 (adamantane-Cs).

*2-(Adamantan-1-yl)-2-oxoethyl 4-chlorobenzoate* (**2d**): Solvent for growing crystal: acetone, ethanol and acetonitrile (1:1:1 *v*/*v*/*v*); yield: 75%; M.P. 411–413 K; FTIR (ATR (solid) cm^−1^): 2910, 2851 (C–H, ν), 1723 (C=O, ν), 1593, 1421 (Ar, C=C, ν), 1269, 1119 (C–O, ν), 752 (C–Cl, ν); ^1^H-NMR (500 MHz, CDCl_3_): δ ppm 8.03–8.01 (d, 2H, *J* = 8.8 Hz, Ar), 7.43–7.41 (d, 2H, *J* = 8.8 Hz, Ar), 5.09 (s, 2H, -CH_2_), 2.08 (br-s, 3H, adamantane-CH), 1.93–1.92 (br-d, 6H, adamantane-CH_2_), 1.80–1.72 (br-q, 6H, adamantane-CH_2_); ^13^C-NMR (125 MHz, CDCl_3_): δ ppm 207.07 (C=O), 165.19 (O-C=O), 139.71, 131.27, 128.76, 128.01 (Ar), 65.11(-CH_2_), 45.37, 37.99, 36.43, 27.75 (adamantane-Cs).

*2-(Adamantan-1-yl)-2-oxoethyl 2,4-dichlorobenzoate* (**2e**): Solvent for growing crystal: acetone; yield: 85%; M.P. 395–397 K; FTIR (ATR (solid) cm^−1^): 2912, 2850 (C–H, ν), 1711 (C=O, ν), 1583, 1417 (Ar, C=C, ν), 1244, 1129, 1098, 1023 (C–O, ν), 829 (C–Cl, ν); ^1^H-NMR (500 MHz, CDCl_3_): δ ppm 7.98–7.96 (d, 1H, *J* = 8.5 Hz, Ar), 7.48 (s, 1H, Ar), 7.33–7.31 (d, 1H, *J* = 8.5 Hz, Ar), 5.11 (s, 2H, -CH_2_), 2.09 (br-s, 3H, adamantane-CH), 1.93–1.92 (br-d, 6H, adamantane-CH_2_), 1.80–1.72 (br-q, 6H, adamantane-CH_2_); ^13^C-NMR (125 MHz, CDCl_3_): δ ppm 206.86 (C=O), 164.01 (O-C=O), 138.66, 135.20, 133.00, 131.00, 127.62, 127.06 (Ar), 65.30(-CH_2_), 45.38, 37.98, 36.42, 27.73 (adamantane-Cs).

*2-(Adamantan-1-yl)-2-oxoethyl 2-methylbenzoate* (**2f**): Solvent for growing crystal: acetone; yield: 83%; M.P. 367–369 K; FTIR (ATR (solid) cm^−1^): 2905, 2851 (C–H, ν), 1708 (C=O, ν), 1602, 1415 (Ar, C=C, ν), 1253, 1026 (C–O, ν), 733 (C–H, ω); ^1^H-NMR (500 MHz, CDCl_3_): δ ppm 8.02–8.00 (d, 1H, *J* = 8.0 Hz, Ar), 7.42–7.39 (t, 1H, *J* = 8.0 Hz, Ar), 7.25–7.24 (m, 2H, Ar), 5.09 (s, 2H, -CH_2_), 2.09 (br-s, 3H, adamantane-CH), 1.94–1.94 (br-d, 6H, adamantane-CH_2_), 1.80–1.72 (br-q, 6H, adamantane-CH_2_); ^13^C-NMR (125 MHz, CDCl_3_): δ ppm 207.41 (C=O), 166.98 (O-C=O), 140.43, 132.19, 131.58, 130.89, 129.06, 125.72 (Ar), 64.74 (-CH_2_), 45.37, 38.03, 36.47, 27.78 (adamantane-Cs), 21.60 (-CH_3_).

*2-(Adamantan-1-yl)-2-oxoethyl 3-methylbenzoate* (**2g**): Solvent for growing crystal: acetone, ethanol and acetonitrile (1:1:1 *v*/*v*/*v*); yield: 75%; M.P. 372–374 K; FTIR (ATR (solid) cm^−1^): 2902, 2848 (C–H, ν), 1724 (C=O, ν), 1588, 1421, (Ar, C=C, ν), 1302, 1279, 1195, 1109 (C–O, ν), 740 (C–H, ω); ^1^H-NMR (500 MHz, CDCl_3_): δ ppm 7.91 (s, 1H, Ar), 7.89–7.88 (d, 2H, *J* = 7.5 Hz, Ar), 7.39–7.37 (d, 1H, *J* = 7.5 Hz, Ar), 7.35–7.32 (t, 1H, *J* = 7.5 Hz Ar), 5.09 (s, 2H, -CH_2_), 2.08 (br-s, 3H, adamantane-CH), 1.94–1.93 (br-d, 6H, adamantane-CH_2_), 1.80–1.72 (br-q, 6H, adamantane-CH_2_); ^13^C-NMR (125 MHz, CDCl_3_): δ ppm 207.32 (C=O), 166.25 (O-C=O), 138.17, 134.00, 130.39, 129.44, 128.29, 127.04 (Ar), 64.91 (-CH_2_), 45.39, 38.01, 36.47, 27.78 (adamantane-Cs), 21.26 (-CH_3_).

*2-(Adamantan-1-yl)-2-oxoethyl 4-methylbenzoate* (**2h**): Solvent for growing crystal: acetone; yield: 81%; M.P. 413–415 K; FTIR (ATR (solid) cm^−1^): 2902, 2853 (C–H, ν), 1714 (C=O, ν), 1610, 1418, (Ar, C=C, ν), 1258, 1115, (C–O, ν), 747 (C–H, ω); ^1^H-NMR (500 MHz, CDCl_3_): δ ppm 7.98–7.97 (d, 2H, *J* = 8.0 Hz, Ar), 7.25–7.23 (d, 2H, *J* = 8.0 Hz, Ar), 5.08 (s, 2H, -CH_2_), 2.41 (s, 3H, -CH_3_), 2.08 (br-s, 3H, adamantane-CH), 1.93–1.93 (br-d, 6H, adamantane-CH_2_), 1.80–1.72 (br-q, 6H, adamantane-CH_2_); ^13^C-NMR (125 MHz, CDCl_3_): δ ppm 207.37 (C=O), 166.12 (O-C=O), 143.93, 129.92, 129.10, 126.80 (Ar), 64.82 (-CH_2_), 45.39, 38.01, 36.47, 27.79 (adamantane-Cs), 21.70 (-CH_3_).

*2-(Adamantan-1-yl)-2-oxoethyl 2-methoxybenzoate* (**2i**): Solvent for growing crystal: acetone and ethanol (1: 1 *v*/*v*); yield: 80%; M.P. 362–364 K; FTIR (ATR (solid) cm^−1^): 2902, 2851 (C–H, ν), 1702 (C=O, ν), 1599, 1442 (Ar, C=C, ν), 1248, 1096, 1020 (C–O, ν), 758 (C–H, ω); ^1^H-NMR (500 MHz, CDCl_3_): δ ppm 7.97–7.95 (d, 1H, *J* = 8.5 Hz, Ar), 7.50–7.47 (t, 1H, *J* = 8.5 Hz, Ar), 7.01–6.97 (t, 2H, *J* = 8.5 Hz, Ar), 5.07 (s, 2H, -CH_2_), 3.91 (s, 3H, -CH_3_), 2.07 (br-s, 3H, adamantane-CH), 1.93–1.93 (br-d, 6H, adamantane-CH_2_), 1.79–1.71 (br-q, 6H, adamantane-CH_2_); ^13^C-NMR (125 MHz, CDCl_3_): δ ppm 207.41 (C=O), 165.23 (O-C=O), 159.52, 133.90, 132.21, 120.17, 119.19, 111.99 (Ar), 64.72 (-CH_2_), 56.03 (-CH_3_), 45.38, 38.01, 36.48, 27.79 (adamantane-Cs).

*2-(Adamantan-1-yl)-2-oxoethyl 3-methoxybenzoate* (**2j**): Solvent for growing crystal: acetone and ethanol (1: 1 *v*/*v*); Yield: 74%; M.P. 430–432 K; FTIR (ATR (solid) cm^−1^): 2926, 2853 (C–H, ν), 1711 (C=O, ν), 1584, 1489 (Ar, C=C, ν), 1288, 1221, 1029 (C–O, ν), 759 (C–H, ω). ^1^H-NMR (500 MHz, CDCl_3_): δ ppm 7.70–7.68 (d, 1H, *J* = 8.0 Hz, Ar), 7.60 (s, 1H, Ar), 7.37–7.33 (t, 1H, *J* = 8.0 Hz, Ar), 7.13–7.11 (m, 1H, Ar), 5.09 (s, 2H, -CH_2_), 3.85 (s, 3H, -CH_3_), 2.08 (br-s, 3H, adamantane-CH), 1.94–1.93 (br-d, 6H, adamantane-CH_2_), 1.80–1.72 (br-q, 6H, adamantane-CH_2_); ^13^C-NMR (125 MHz, CDCl_3_): δ ppm 207.18 (C=O), 165.96 (O-C=O), 159.56, 130.79, 129.42, 122.35, 120.04, 114.05 (Ar), 65.04 (-CH_2_), 55.44 (-CH_3_), 45.39, 38.01, 36.46, 27.77 (adamantane-Cs).

*2-(Adamantan-1-yl)-2-oxoethyl 4-methoxybenzoate* (**2k**): Solvent for growing crystal: acetone and ethanol (1: 1 *v*/*v*); yield: 80%; M.P. 390–392 K; FTIR (ATR (solid) cm^−1^): 2903, 2851 (C–H, ν), 1710 (C=O, ν), 1605, 1417 (Ar, C=C, ν), 1253, 1167, 1028 (C–O, ν), 767 (C–H, ω); ^1^H-NMR (500 MHz, CDCl_3_): δ ppm 8.05–8.04 (d, 2H, *J* = 9.0 Hz, Ar), 6.93–6.91 (d, 2H, *J* = 9.0 Hz Ar), 5.07 (s, 2H, -CH_2_), 3.86 (s, 3H, -CH_3_), 2.08 (br-s, 3H, adamantane-CH), 1.93–1.93 (br-d, 6H, adamantane-CH_2_), 1.80–1.72 (br-q, 6H, adamantane-CH_2_); ^13^C-NMR (125 MHz, CDCl_3_): δ ppm 207.54 (C=O), 165.77 (O-C=O), 163.61, 131.96, 121.94, 113.66 (Ar), 64.73 (-CH_2_), 55.44 (-CH_3_), 45.39, 38.02, 36.47, 27.79 (adamantane-Cs).

*2-(Adamantan-1-yl)-2-oxoethyl 2-nitrobenzoate* (**2l**): Solvent for growing crystal: acetone, ethanol and acetonitrile (1:1:1 *v*/*v*/*v*); yield: 70%; M.P. 391–393 K; FTIR (ATR (solid) cm^−1^): 2919, 2850 (C–H, ν), 1731 (C=O, ν), 1579, 1450 (Ar, C=C, ν), 1535, 1353 (N–O, ν), 1290, 1132, 1079 (C–O, ν), 732 (C–H, ω); ^1^H-NMR (500 MHz, CDCl_3_): δ ppm 7.97–7.95 (d, 1H, *J* = 7.5 Hz, Ar), 7.94–7.92 (d, 1H, *J* = 7.5 Hz Ar), 7.73–7.70 (t, 1H, *J* = 7.5 Hz, Ar), 7.66–7.63 (t, 1H, *J* = 7.5 Hz, Ar), 5.12 (s, 2H, -CH_2_), 2.09 (br-s, 3H, adamantane-CH), 1.92(br-s, 6H, adamantane-CH_2_), 1.78–1.74 (br-d, 6H, adamantane-CH_2_); ^13^C-NMR (125 MHz, CDCl_3_): δ ppm 206.75 (C=O), 164.97 (O-C=O), 147.74, 133.17, 131.78, 130.35, 127.46, 123.94 (Ar), 65.92 (-CH_2_), 45.39, 37.95, 36.41, 27.72 (adamantane-Cs).

*2-(Adamantan-1-yl)-2-oxoethyl 3-nitrobenzoate* (**2m**): yield: 72%; M.P. 385–387 K; FTIR (ATR (solid) cm^−1^): 3092 (Ar, C–H, ν), 2917, 2851 (C–H, ν), 1727 (C=O, ν), 1631, 1421 (Ar, C=C, ν), 1533, 1347 (N–O, ν), 1297, 1261, 1131 (C–O, ν), 716 (C–H, ω); ^1^H-NMR (500 MHz, CDCl_3_): δ ppm 8.92 (s, 1H, Ar), 8.45–8.43 (d, 1H, *J* = 8.1 Hz, Ar), 8.42–8.40 (d, 1H, *J* = 8.1 Hz, Ar), 7.69–7.65 (t, 1H, *J* = 8.1 Hz, Ar), 5.17 (s, 2H, -CH_2_), 2.10 (br-s, 3H, adamantane-CH), 1.94 (br-s, 6H, adamantane-CH_2_), 1.79–1.76 (br-d, 6H, adamantane-CH_2_); ^13^C-NMR (125 MHz, CDCl_3_): δ ppm 206.60 (C=O), 164.00 (O-C=O), 148.31, 135.52, 131.35, 129.67, 127.66, 124.92 (Ar), 65.61 (-CH_2_), 45.39, 37.99, 36.42, 27.74 (adamantane-Cs).

*2-(Adamantan-1-yl)-2-oxoethyl 4-nitrobenzoate* (**2n**): Solvent for growing crystal: acetone, ethanol and acetonitrile (1:1:1 *v*/*v*/*v*); yield: 76%; M.P. 437–439 K; FTIR (ATR (solid) cm^−1^): 3112 (Ar, C–H, ν), 2905, 2854 (C–H, ν), 1723 (C=O, ν), 1606, 1422 (Ar, C=C, ν), 1530, 1344 (N–O, ν), 1283, 1118 (C–O, ν), 714 (C–H, ω); ^1^H-NMR (500 MHz, CDCl_3_): δ ppm 8.31–8.29 (d, 2H, *J* = 8.5 Hz, Ar), 8.27–8.25 (d, 2H, *J* = 8.5 Hz Ar), 5.16 (s, 2H, -CH_2_), 2.10 (br-s, 3H, adamantane-CH), 1.94 (br-s, 6H, adamantane-CH_2_), 1.81–1.73 (br-q, 6H, adamantane-CH_2_); ^13^C-NMR (125 MHz, CDCl_3_): δ ppm 206.61 (C=O), 164.21 (O-C=O), 150.72, 134.97, 131.00, 123.56 (Ar), 65.61 (-CH_2_), 45.40, 37.99, 36.41, 27.73 (adamantane-Cs).

*2-(Adamantan-1-yl)-2-oxoethyl 2-aminobenzoate* (**2o**): Solvent for growing crystal: acetone, ethanol and acetonitrile (1:1:1 *v*/*v*); yield: 76%; M.P. 440–442 K; FTIR (ATR (solid) cm^−1^): 3497, 3373 (N–H, ν), 2902, 2848 (C–H, ν), 1695 (C=O, ν), 1613, 1418 (Ar, C=C, ν), 1581 (N–H, δ), 1243, 1104 (C–O, ν), 746 (C–H, ω); ^1^H-NMR (500 MHz, CDCl_3_): δ ppm 7.95–7.94 (d, 1H, *J* = 8.3 Hz, Ar), 7.29–7.26 (t, 1H, *J* = 8.3 Hz Ar), 6.67–6.64 (m, 2H, Ar), 5.07 (s, 2H, -CH_2_), 2.08 (br-s, 3H, adamantane-CH), 1.93 (br-s, 6H, adamantane-CH_2_), 1.80–1.72 (br-d, 6H, adamantane-CH_2_); ^13^C-NMR (125 MHz, CDCl_3_): δ ppm 207.64 (C=O), 167.29 (O-C=O), 150.52, 134.38, 131.63, 116.67, 116.41, 110.34 (Ar), 64.46 (-CH_2_), 45.38, 38.05, 36.47, 27.79 (adamantane-Cs).

*2-(Adamantan-1-yl)-2-oxoethyl 3-aminobenzoate* (**2p**): Solvent for growing crystal: acetone, ethanol and acetonitrile (1:1:1 *v*/*v*/*v*); yield: 70%; M.P. 396–398 K; FTIR (ATR (solid) cm^−1^): 3465, 3347 (N–H, ν), 2902, 2849 (C–H, ν), 1700 (C=O, ν), 1631, 1461 (Ar, C=C, ν), 1603 (N–H, δ), 1248, 1113 (C–O, ν), 751 (C–H, ω); ^1^H-NMR (500 MHz, CDCl_3_): δ ppm 7.50–7.49 (d, 1H, *J* = 7.9 Hz, Ar), 7.42 (s, 1H, Ar), 7.24–7.21 (t, 1H, *J* = 7.9 Hz, Ar), 6.91–6.89 (d, 1H, *J* = 7.9 Hz, Ar), 5.08 (s, 2H, -CH_2_), 2.08 (br-s, 3H, adamantane-CH), 1.93–1.92 (br-d, 6H, adamantane-CH_2_), 1.79–1.72 (br-q, 6H, adamantane-CH_2_); ^13^C-NMR (125 MHz, CDCl_3_): δ ppm 207.36 (C=O), 166.17 (O-C=O), 145.98, 130.49, 129.34, 120.42, 119.99, 116.28 (Ar), 64.91 (-CH_2_), 45.39, 38.00, 36.46, 27.77(adamantane-Cs).

*2-(Adamantan-1-yl)-2-oxoethyl 4-aminobenzoate* (**2q**): yield: 76%; M.P. 428–430 K; FTIR (ATR (solid) cm^−1^): 3493, 3362 (N–H, ν), 2906, 2850 (C–H, ν), 1697 (C=O, ν), 1619, 1414 (Ar, C=C, ν), 1600 (N–H, δ), 1276, 1118 (C–O, ν), 768 (C–H, ω); ^1^H-NMR (500 MHz, CDCl_3_): δ ppm 7.91–7.89 (d, 2H, *J* = 8.5 Hz, Ar), 6.65–6.63 (d, 2H, *J* = 8.5 Hz, Ar), 5.04 (s, 2H, -CH_2_), 2.07 (br-s, 3H, adamantane-CH), 1.93–1.92 (br-d, 6H, adamantane-CH_2_), 1.79–1.71 (br-q, 6H, adamantane-CH_2_); ^13^C-NMR (125 MHz, CDCl_3_): δ ppm 207.90 (C=O), 166.03 (O-C=O), 151.08, 132.00, 119.04, 113.92 (Ar), 64.50 (-CH_2_), 45.57, 38.02, 36.60, 27.77 (adamantane-Cs).

*2-(Adamantan-1-yl)-2-oxoethyl 2-pyridinecarboxylate* (**2r**): Solvent for growing crystal: acetone, ethanol and acetonitrile (1:1:1 *v*/*v*/*v*); yield: 76%; M.P. 402–404 K; FTIR (ATR (solid) cm^−1^): 2903, 2849 (C–H, ν), 1707 (C=O, ν), 1582, 1419 (Ar, C=C, ν), 1304 (C–N, ν) 1242, 1128 (C–O, ν), 745 (C–H, ω); ^1^H-NMR (500 MHz, CDCl_3_): δ ppm 8.79–8.78 (d, 1H, *J* = 4.7 Hz, Ar), 8.19–8.17 (d, 1H, *J* = 4.7 Hz, Ar), 7.88–7.86 (t, 1H, *J* = 4.7 Hz, Ar), 7.52–7.50 (q, 1H, *J* = 4.7 Hz, Ar), 5.20 (s, 2H, -CH_2_), 2.09 (br-s, 3H, adamantane-CH), 1.93–1.93 (br-d, 6H, adamantane-CH_2_), 1.80–1.72 (br-q, 6H, adamantane-CH_2_); ^13^C-NMR (125 MHz, CDCl_3_): δ ppm 206.36 (C=O), 164.54 (O-C=O), 149.86, 147.41, 137.12, 127.18, 125.53 (Ar), 65.70 (-CH_2_), 45.31, 37.99, 36.44, 27.75 (adamantane-Cs).

### 3.3. Bioactivity Methods

#### 3.3.1. Hydrogen Peroxide Radical Scavenging Assay

The abilities of adamantyl-based compounds to scavenge hydroxyl radical were determined by following the method of [50] with some modifications. The solution of hydrogen peroxide (40 mM) was prepared in PBS (phosphate-buffered saline). The adamantyl-based derivatives with a concentration of 250 μg/mL were prepared in PBS and added to 0.6 mL of hydrogen peroxide solution. The mixture was mixed well and incubated at room temperature for 10 min. The absorbance was determined at 230 nm by using a spectrophotometer. Ascorbic acid was used as a standard drug, while PBS was used as the blank. The percentage of hydrogen peroxide scavenging activity was calculated by using the following formula:

(1) $$\text{Percentage of scavenging activity}=\frac{\text{Absorbance of control}-\text{Absorbance of sample}}{\text{Absorbance of control}}\times 100$$

#### 3.3.2. DPPH Radical Scavenging Assay

The DPPH radical scavenging assay was used to evaluate the antioxidant properties of the adamantyl-based compounds. This assay was carried out following the method of [51]. The DPPH solution (0.16 mM) was prepared in ethanol. The adamantyl-based derivatives were prepared in ethanol with concentrations of 250, 500 and 1000 μg/mL. Approximately 100 μL of each adamantyl-based derivative were mixed with 100 μL of DPPH solution in a 96-well plate. Each sample and the control were prepared in triplicate. The plate was then incubated in a dark room at room temperature for 30 min, and the absorbance was measured at 517 nm with a microplate reader. Ascorbic acid was used as a standard drug. The percentage of DPPH free radical scavenging activity was calculated by using the following formula:

(2) $$\text{Percentage of scavenging activity}=\frac{\text{Absorbance of blank}-\text{Absorbance of sample}}{\text{Absorbance of blank}}\times 100$$

#### 3.3.3. Protein Denaturation Assay

Adamantyl-based compounds were tested on egg albumin to observe their anti-inflammatory activity against protein denaturation. The assay was performed following the method described by [52] with some modifications. The 2.5 mL of reaction mixture consisted of 0.1 mL of egg albumin, 1.4 mL of PBS and 1 mL of adamantyl-based derivatives with a concentration of 250 μg/mL. A similar volume of PBS with egg albumin was used as the control. The mixture solutions were incubated at 37 °C for 15 min and then heat in a water bath at 70 °C for 5 min. After cooling to room temperature, the absorbance was measured at 660 nm using a microplate reader. Diclofenac sodium was used as the standard drug. The percentage of inhibition of protein denaturation was calculated by using the following formula:

(3) $$\text{Percentage of scavenging activity}=\frac{\text{Absorbance of control}-\text{Absorbance of sample}}{\text{Absorbance of control}}\times 100$$

## 4. Conclusions

A series of 2-(adamantan-1-yl)-2-oxoethyl benzoates, **2**(**a**–**q**), and 2-(adamantan-1-yl)-2-oxoethyl 2-pyridinecarboxylate, **2r**, were synthesized and structurally characterized by FTIR, NMR and single-crystal X-ray diffraction analysis. Introduction of the adamantane moiety into the synthesis of 2-oxopropyl benzoate derivatives produced all molecular structures in synclinal conformation. In crystals, molecules are commonly packed in a head-to-tail (adamantane moiety to phenyl moiety) pattern. These similarities lead to redundant 2D or 3D structural similarity in adamantane-based ester derivatives. The adamantyl-based compounds show selective antioxidant abilities and good hydrogen peroxide radical scavenging activities, especially 2-(adamantan-1-yl)-2-oxoethyl 2-chlorobenzoate, which outperformed the standard compound. Besides that, three nitrogen-containing adamantane compounds, **2p**, **2q** and **2r**, show strong anti-inflammatory effects towards protein denaturation, which performed better than diclofenac sodium. Thus, further modifications of adamantane compounds with nitrogen-containing groups can potentially produce promising anti-inflammatory agents for clinical use in the future.

## Supplementary Materials

Supplementary materials can be accessed at: http://www.mdpi.com/1420-3049/20/10/18827/s1.

## References

[1] Piérard G.E., Piérard-Franchimont C., Paquet P., Quatresooz P. (2009). Spotlight on adapalene. Expert Opin. Drug Metab. Toxicol..

[2] Reisberg B., Doody R., Stöffler A., Schmitt F., Ferris S., Möbius H.J. (2003). Memantine in moderate-to-severe alzheimer’s disease. N. Engl. J. Med..

[3] Rosenthal K.S., Sokol M.S., Ingram R.L., Subramanian R., Fort R.C. (1982). Tromantadine: Inhibitor of early and late events in herpes simplex virus replication. Antimicrob. Agents Chemother..

[4] Kelly J.M., Miles M.A., Skinner A.C. (1999). The anti-influenza virus drug rimantadine has trypanocidal activity. Antimicrob. Agents Chemother..

[5] De Clercq E. (2006). Antiviral agents active against influenza a viruses. Nat. Rev. Drug Discov..

[6] Cady S.D., Luo W., Hu F., Hong M. (2009). Structure and function of the influenza a M2 proton channel. Biochemistry.

[7] Zoidis G., Kolocouris N., Kelly J.M., Prathalingam S.R., Naesens L., de Clercq E. (2010). Design and synthesis of bioactive adamantanaminoalcohols and adamantanamines. Eur. J. Med. Chem..

[8] Von Geldern T.W., Trevillyan J.M. (2006). The next big thing in diabetes: Clinical progress on DPP-IV inhibitors. Drug Dev. Res..

[9] Havale S.H., Pal M. (2009). Medicinal chemistry approaches to the inhibition of dipeptidyl peptidase-4 for the treatment of type 2 diabetes. Bioorg. Med. Chem..

[10] Zettl H., Schubert-Zsilavecz M., Steinhilber D. (2010). Medicinal chemistry of incretin mimetics and DPP-4 inhibitors. ChemMedChem.

[11] Liu J., Obando D., Liao V., Lifa T., Codd R. (2011). The many faces of the adamantyl group in drug design. Eur. J. Med. Chem..

[12] Al-Omar M.A., Al-Abdullah E.S., Shehata I.A., Habib E.E., Ibrahim T.M., El-Emam A.A. (2010). Synthesis, antimicrobial, and anti-inflammatory activities of novel 5-(1-adamantyl)-4-arylideneamino-3-mercapto-1,2,4-triazoles and related derivatives. Molecules.

[13] Lamanna G., Russier J., Dumortier H., Bianco A. (2012). Enhancement of anti-inflammatory drug activity by multivalent adamantane-based dendrons. Biomaterials.

[14] Antoniadou-Vyza E., Avramidis N., Kourounakis A., Hadjipetrou L. (1998). Anti-inflammatory properties of new adamantane derivatives. Design, synthesis, and biological evaluation. Arch. Pharm..

[15] Priyanka B., Anitha K., Shirisha S., Dipankar B., Rajesh K. (2013). Evaluation of anti-oxidant activity of ethanolic root extract of albizla lebbeck l. Int. Res. J. Pharm. Appl. Sci..

[16] Joyce D.A. (1987). Oxygen radicals in disease. Advers. Drug React. Bull..

[17] Farber J.L. (1994). Mechanisms of cell injury by activated oxygen species. Environ. Health Perspect..

[18] Lee C.T., Repasky E.A. (2012). Opposing roles for heat and heat shock proteins in macrophage functions during inflammation: A function of cell activation state?. Front. Immunol..

[19] Saso L., Valentini G., Casini M., Grippa E., Gatto M., Leone M., Silvestrini B. (2001). Inhibition of heat-induced denaturation of albumin by nonsteroidal antiinflammatory drugs (NSAIDs): Pharmacological implications. Arch. Pharm. Res..

[20] Kumar Chandraju Sadolalu C., Chia Tze S., Ooi Chin W., Quah Ching K., Chandraju S., Fun H.K. (2014). Conformational studies of 2-(4-bromophenyl)-2-oxoethyl benzoates. Z. Kristallogr. Cryst. Mater..

[21] Jin Y., Guo J.N., Lin K., Tang G., Zhao Y.F. (2008). Benzoylmethyl 4-chlorobenzoate. Acta Crystallogr. Sect. E Struct. Rep. Online.

[22] Komarov I.V., Gorichko M.V., Shishkin O.V., Kornilov M.Y. (1999). Short communications—Unusual by-product in the bromination of 3,3-dibromocamphor. Russ. J. Org. Chem..

[23] Fun H.K., Shahani T., Garudachari B., Isloor A.M., Satyanarayan M.N. (2011). 2-(4-bromophenyl)-2-oxoethyl 4-methylbenzoate. Acta Crystallogr. Sect. E Struct. Rep. Online.

[24] Fun H.K., Quah C.K., Vijesh A.M., Isloor A.M., Arulmoli T. (2011). 2-(4-chlorophenyl)-2-oxoethyl 3,4-dimethoxybenzoate. Acta Crystallogr. Sect. E Struct. Rep. Online.

[25] Fun H.K., Chia T.S., Shenvi S., Isloor A.M., Garudachari B. (2011). 2-(2,4-dichlorophenyl)-2-oxoethyl 4-methoxybenzoate. Acta Crystallogr. Sect. E Struct. Rep. Online.

[26] Fun H.K., Loh W.S., Garudachari B., Isloor A.M., Satyanarayan M.N. (2011). 2-(4-fluorophenyl)-2-oxoethyl 4-methoxybenzoate. Acta Crystallogr. Sect. E Struct. Rep. Online.

[27] Khan I., Ibrar A., Korzanski A., Kubicki M. (2012). 2-(4-methylphenyl)-2-oxoethyl 3-bromobenzoate. Acta Crystallogr. Sect. E Struct. Rep. Online.

[28] Chidan Kumar C.S., Yohannan Panicker C., Fun H.-K., Sheena Mary Y., Harikumar B., Chandraju S., Quah C.K., Ooi C.W. (2014). Molecular structure, FT-IR, first order hyperpolarizability, NBO analysis, HOMO and LUMO analysis of 2-(4-chlorophenyl)-2-oxoethyl 3-methylbenzoate by HF and density functional methods. Spectrochim. Acta A.

[29] Chidan Kumar C.S., Panicker C.Y., Fun H.-K., Mary Y.S., Harikumar B., Chandraju S., Quah C.K., Ooi C.W. (2014). FT-IR, molecular structure, first order hyperpolarizability, HOMO and LUMO analysis, MEP and NBO analysis of 2-(4-chlorophenyl)-2-oxoethyl 3-nitrobenzoate. Spectrochim. Acta A.

[30] Fun H.K., Arshad S., Garudachari B., Isloor A.M., Satyanarayan M.N. (2011). 2-oxo-2-phenylethyl benzoate. Acta Crystallogr. Sect. E Struct. Rep. Online.

[31] Fun H.K., Arshad S., Garudachari B., Isloor A.M., Satyanarayan M.N. (2011). 2-(4-bromophenyl)-2-oxoethyl 4-bromobenzoate. Acta Crystallogr. Sect. E Struct. Rep. Online.

[32] Fun H.K., Loh W.S., Garudachari B., Isloor A.M., Satyanarayan M.N. (2011). 2-(4-bromophenyl)-2-oxoethyl 4-methoxybenzoate. Acta Crystallogr. Sect. E Struct. Rep. Online.

[33] Fun H.K., Loh W.S., Garudachari B., Isloor A.M., Satyanarayan M.N. (2011). 2-(4-chlorophenyl)-2-oxoethyl 3-(trifluoromethyl)benzoate. Acta Crystallogr. Sect. E Struct. Rep. Online.

[34] Chidan Kumar C.S., Fun H.K., Tursun M., Ooi C.W., Chandraju S., Quah C.K., Parlak C. (2014). Synthesis, molecular structure, FT-IR and XRD investigations of 2-(4-chlorophenyl)-2-oxoethyl 2-chlorobenzoate: A comparative DFT study. Spectrochim. Acta A.

[35] Fun H.K., Arshad S., Garudachari B., Isloor A.M., Satyanarayan M.N. (2011). 2-(4-chlorophenyl)-2-oxoethyl 2,4-difluorobenzoate. Acta Crystallogr. Sect. E Struct. Rep. Online.

[36] Fun H.K., Arshad S., Garudachari B., Isloor A.M., Shivananda K.N. (2011). 2-(4-fluorophenyl)-2-oxoethyl 3-(trifluoromethyl)benzoate. Acta Crystallogr. Sect. E Struct. Rep. Online.

[37] Fun H.K., Asik S.I.J., Garudachari B., Isloor A.M., Satyanarayan M.N. (2011). 2-(4-chlorophenyl)-2-oxoethyl 2-methoxybenzoate. Acta Crystallogr. Sect. E Struct. Rep. Online.

[38] Fun H.K., Loh W.S., Garudachari B., Isloor A.M., Satyanarayana M.N. (2011). 2-(4-bromophenyl)-2-oxoethyl 4-hydroxybenzoate. Acta Crystallogr. Sect. E Struct. Rep. Online.

[39] Fun H.K., Yeap C.S., Garudachari B., Isloor A.M., Satyanarayan M.N. (2011). 2-(4-bromophenyl)-2-oxoethyl 4-chlorobenzoate. Acta Crystallogr. Sect. E Struct. Rep. Online.

[40] Fun H.K., Loh W.S., Garudachari B., Isloor A.M., Satyanarayana M.N. (2011). 2-(4-chlorophenyl)-2-oxoethyl 4-methylbenzoate. Acta Crystallogr. Sect. E Struct. Rep. Online.

[41] Fun H.K., Quah C.K., Garudachari B., Isloor A.M., Satyanarayan M.N. (2011). 2-(4-bromophenyl)-2-oxoethyl 2-methoxybenzoate. Acta Crystallogr. Sect. E Struct. Rep. Online.

[42] Fun H.K., Ooi C.W., Garudachari B., Isloor A.M., Satyanarayan M.N. (2011). 2-(4-bromophenyl)-2-oxoethyl 2-methylbenzoate. Acta Crystallogr. Sect. E Struct. Rep. Online.

[43] Fun H.K., Shahani T., Garudachari B., Isloor A.M., Shivananda K.N. (2011). 2-(4-chlorophenyl)-2-oxoethyl 4-hydroxybenzoate. Acta Crystallogr. Sect. E Struct. Rep. Online.

[44] Fun H.K., Shahani T., Garudachari B., Isloor A.M., Satyganarayan M.N. (2011). 2-(4-chlorophenyl)-2-oxoethyl benzoate. Acta Crystallogr. Sect. E: Struct. Rep. Online.

[45] Ji T., Wang X.L., Gao Y.X., Tang G., Zhao Y.F. (2007). Benzoylmethyl 4-methoxybenzoate. Acta Crystallogr. Sect. E Struct. Rep. Online.

[46] Isloor A.M., Garudachari B., Satyanarayan M.N., Gerber T., Hosten E., Betz R. (2012). 2-(4-fluorophenyl)-2-oxoethyl 2-methoxybenzoate. Acta Crystallogr. Sect. E Struct. Rep. Online.

[47] Bruker (2009). Apex2, Saint and Sadabs.

[48] Sheldrick G.M. (2008). A short history of shelx. Acta Cryst..

[49] Dolomanov O.V., Bourhis L.J., Gildea R.J., Howard J.A., Puschmann H. (2009). *OLEX2*: A complete structure solution, refinement and analysis program. J. Appl. Cryst..

[50] Ruch R.J., Cheng S., Klaunig J.E. (1989). Prevention of cytotoxicity and inhibition of intercellular communication by antioxidant catechins isolated from chinese green tea. Carcinogenesis.

[51] Farasat M., Khavari-Nejad R.A., Nabavi S.M.B., Namjooyan F. (2014). Antioxidant activity, total phenolics and flavonoid contents of some edible green seaweeds from northern coasts of the persian gulf. Iran. J. Pharm. Res..

[52] Ashok Kumar B.S., Saran G., Harshada R., Manoj B., Archana P.G. (2014). Evaluation of anti-arthritic activity of vitex negundo by *in vitro* protein denaturation method. J. Tradit. Med..

