# Supplementary material for: Synthesis and Crystallographic Insight into the Structural Aspects of Some Novel Adamantane-Based Ester Derivatives
Source: Molecules. 2015 Oct 16;20(10):18827–46. doi: 10.3390/molecules201018827 (PMC6331964; doi:10.3390/molecules201018827)
Supplement: Supplementary file 1 [file molecules-20-18827-s001.pdf]

Supplementary Materials

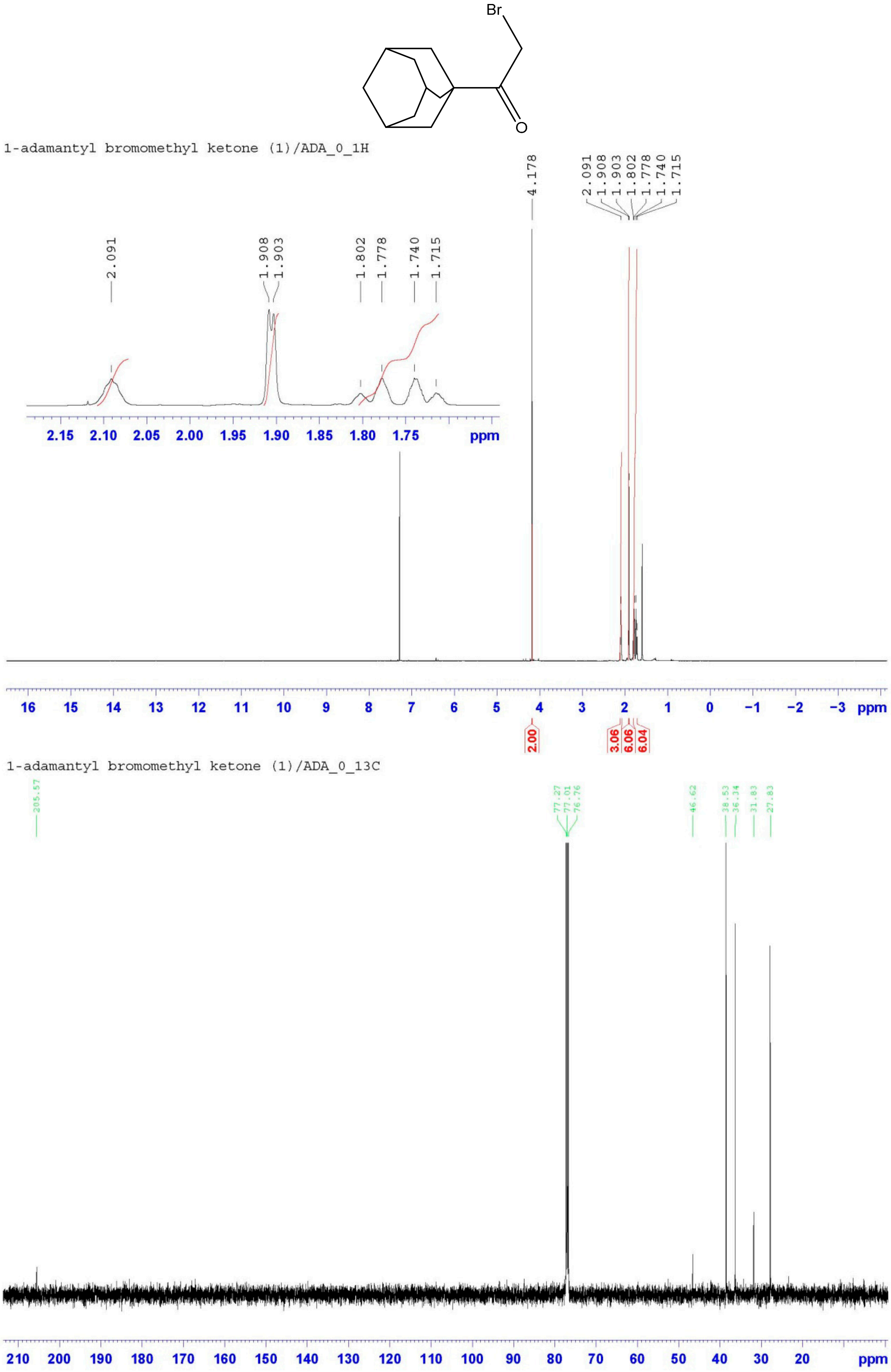

Figure S1. ^{1}H-NMR and ^{13}C-NMR spectra of *1-adamantyl bromomethyl ketone* (**1**).

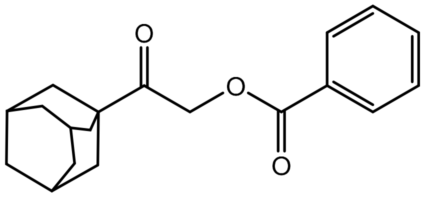

2-(Adamantan-1-yl)-2-oxoethyl benzoate (2a)/ADA_1_1H

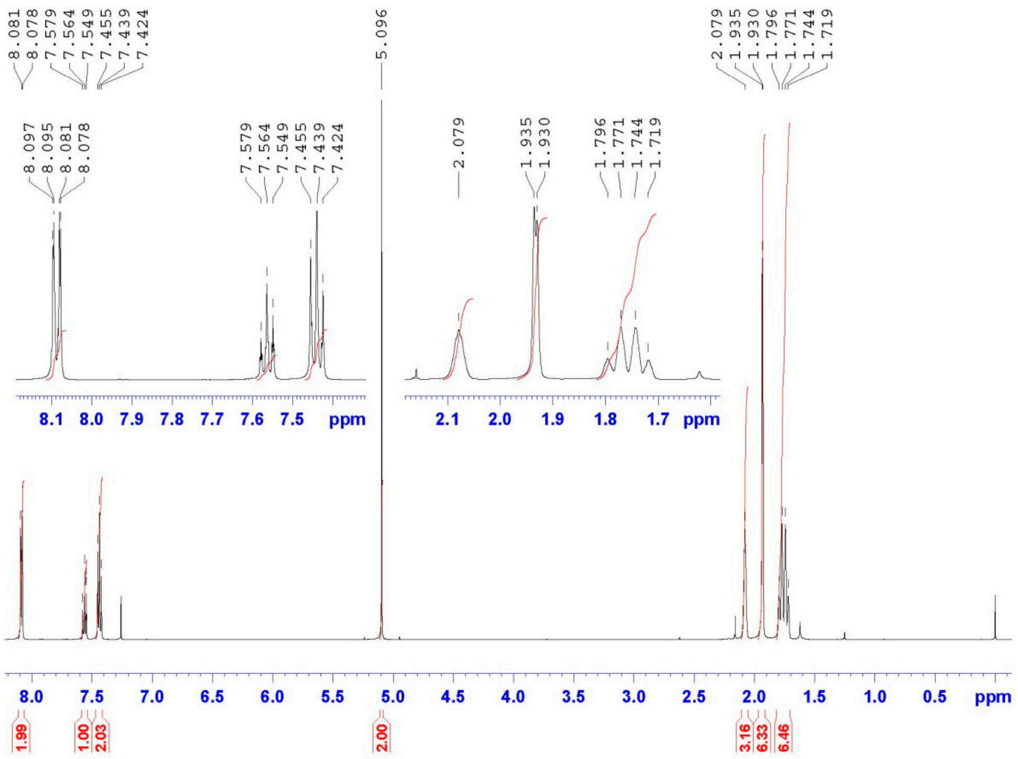

2-(Adamantan-1-yl)-2-oxoethyl benzoate (2a)/ADA_1_13C

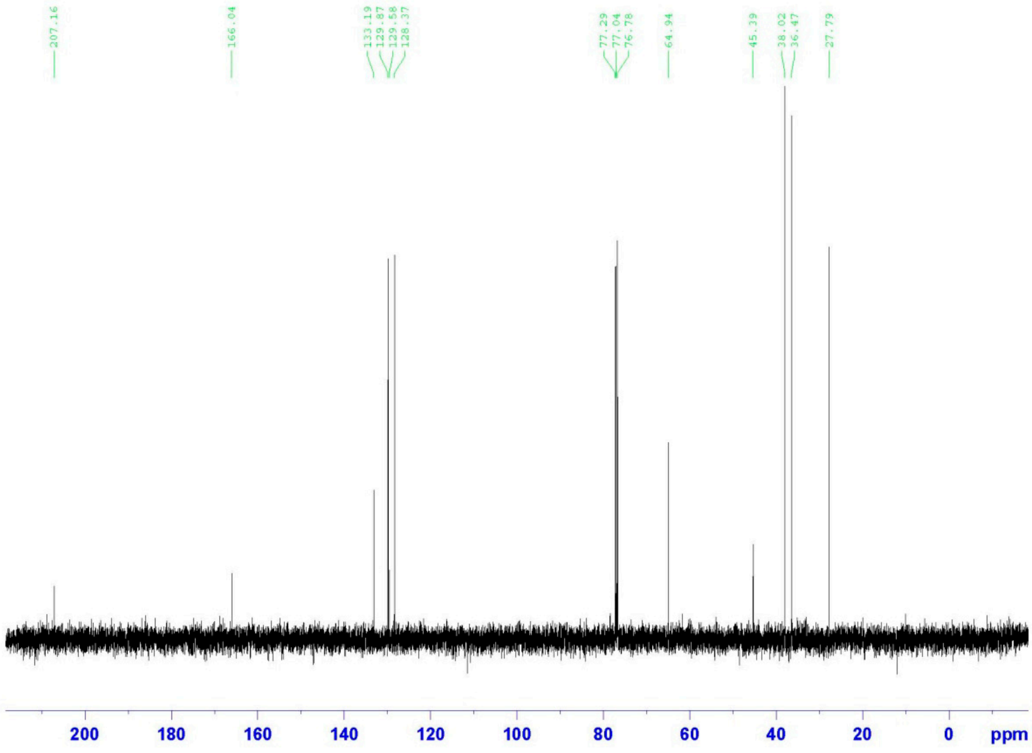

Figure S2. *Cont.*

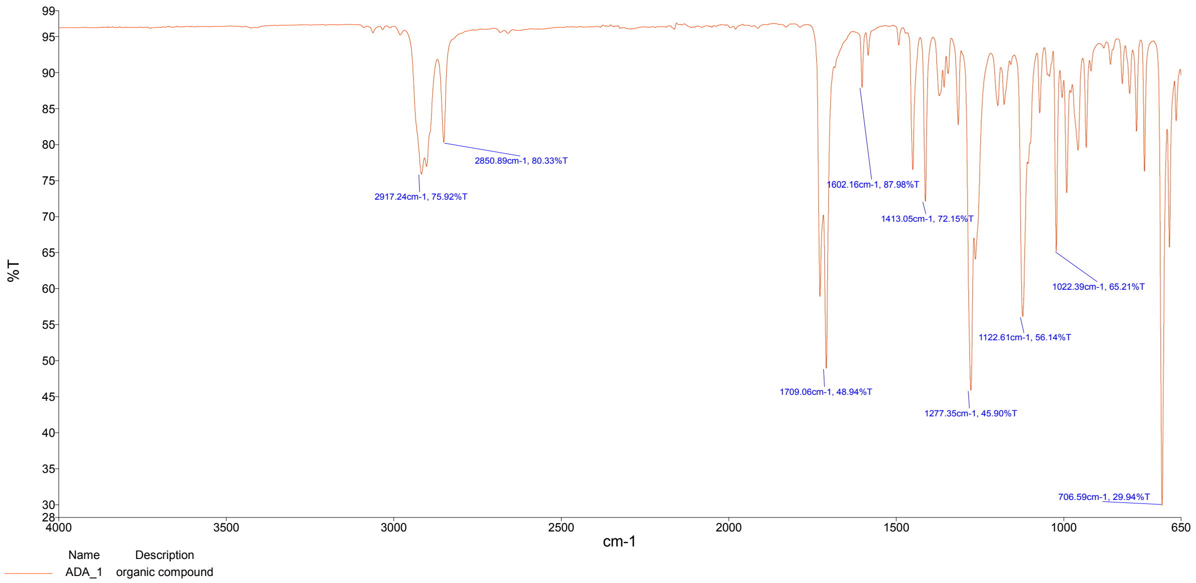

Figure S2. ^{1}H-NMR, ^{13}C-NMR and FTIR spectra of *2-(Adamantan-1-yl)-2-oxoethyl benzoate* (**2a**).

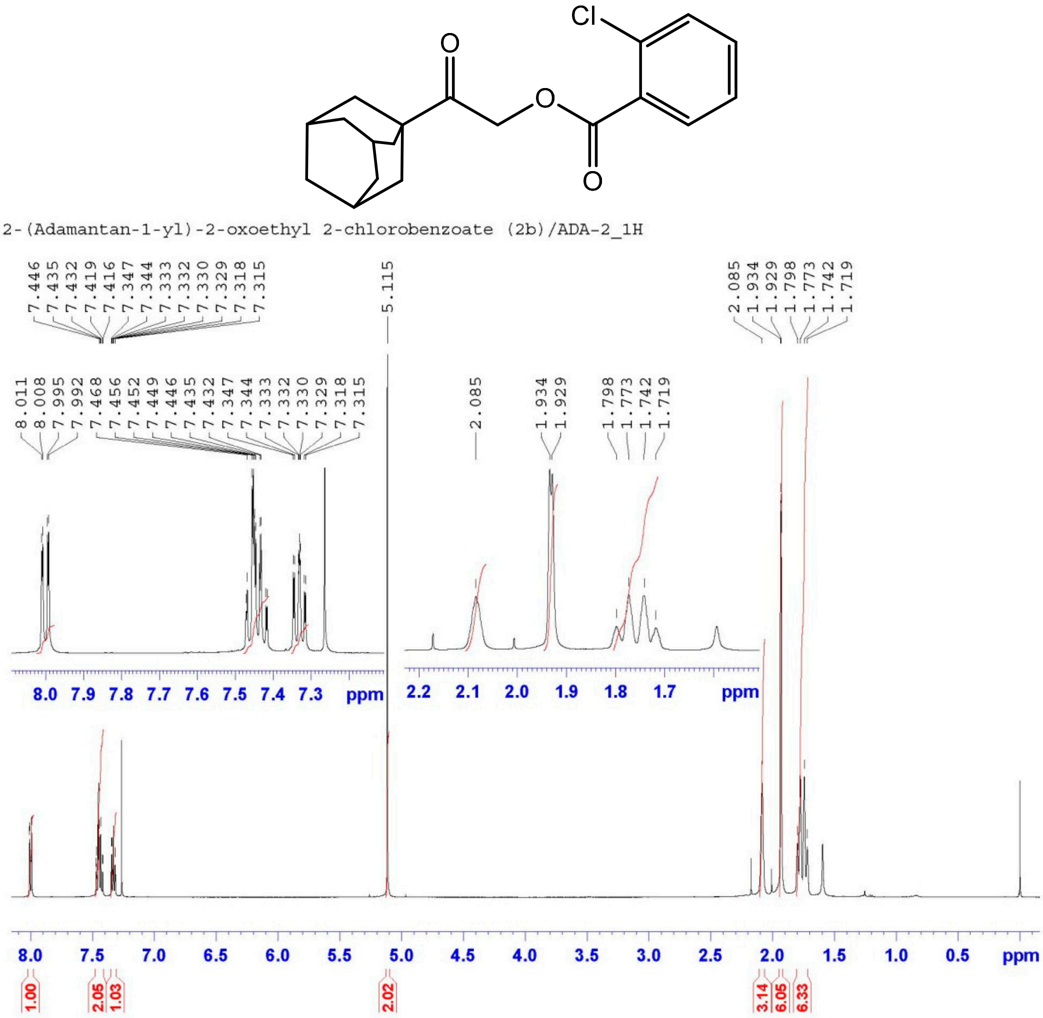

Figure S3. *Cont.*

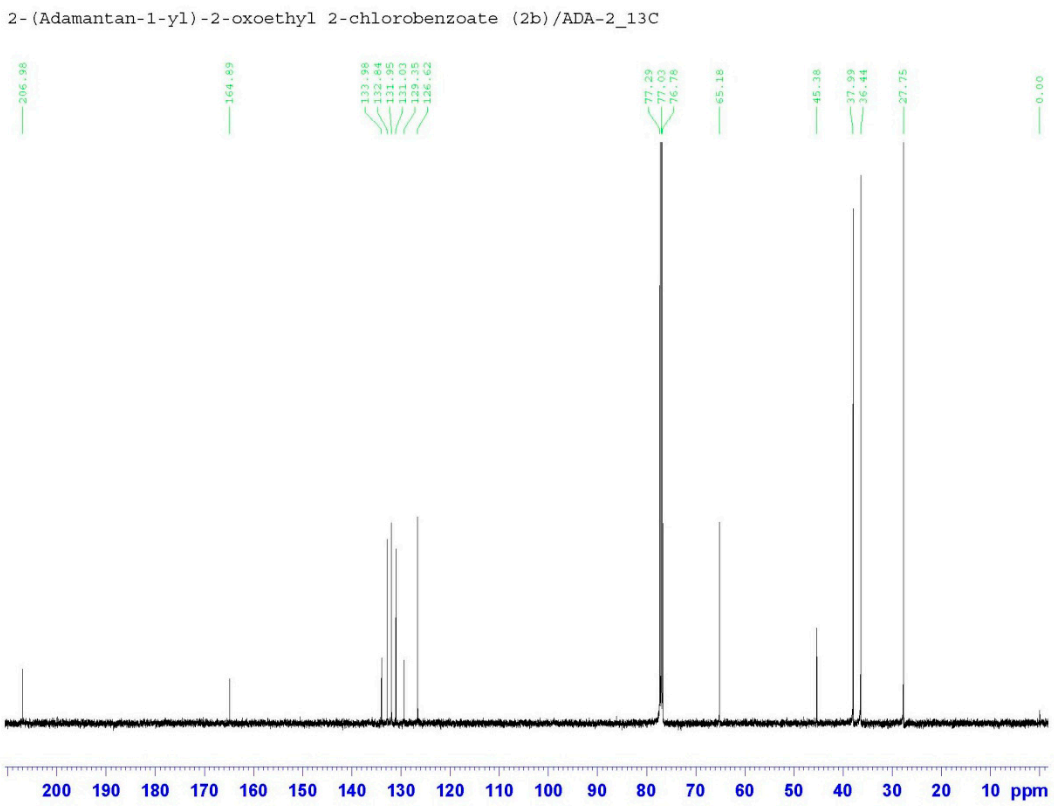

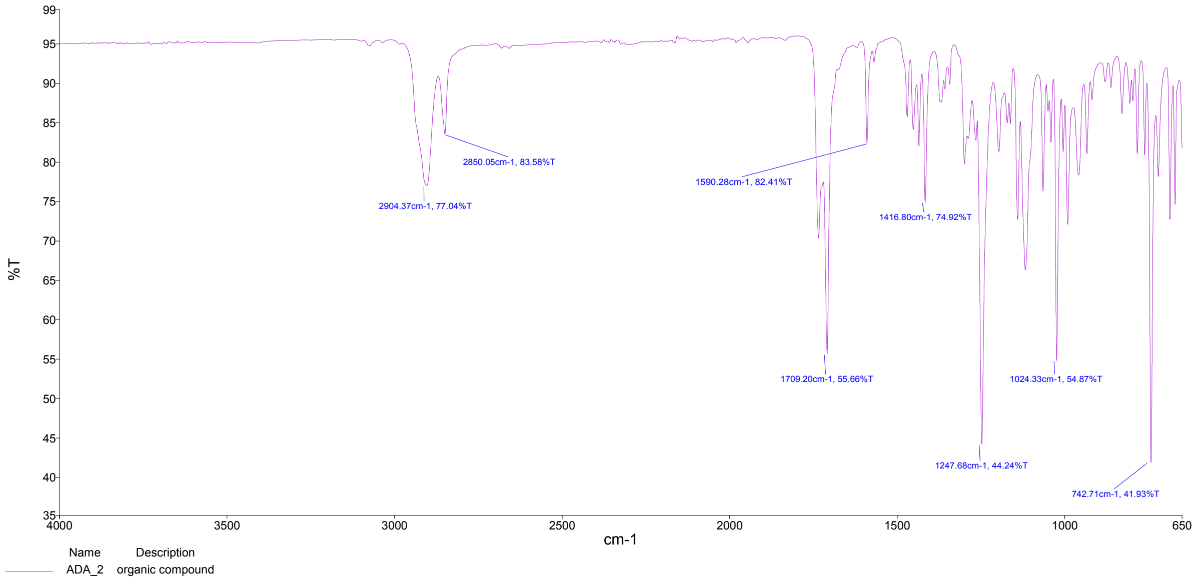

Figure S3. ^{1}H-NMR, ^{13}C-NMR and FTIR spectra of *2-(Adamantan-1-yl)-2-oxoethyl 2-chlorobenzoate* (**2b**).

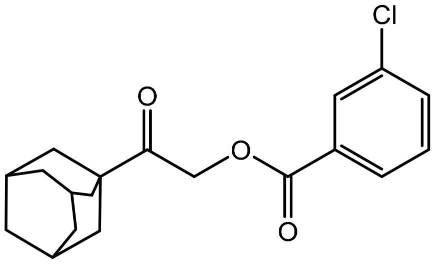

Figure S4. *Cont.*

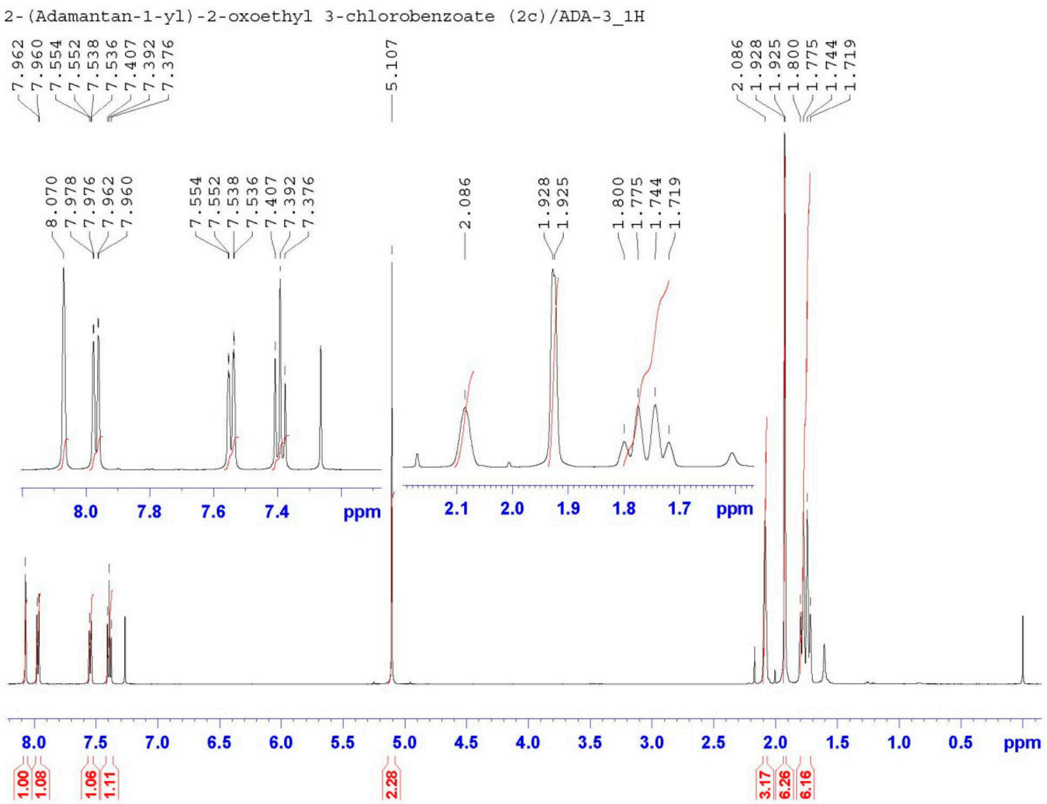

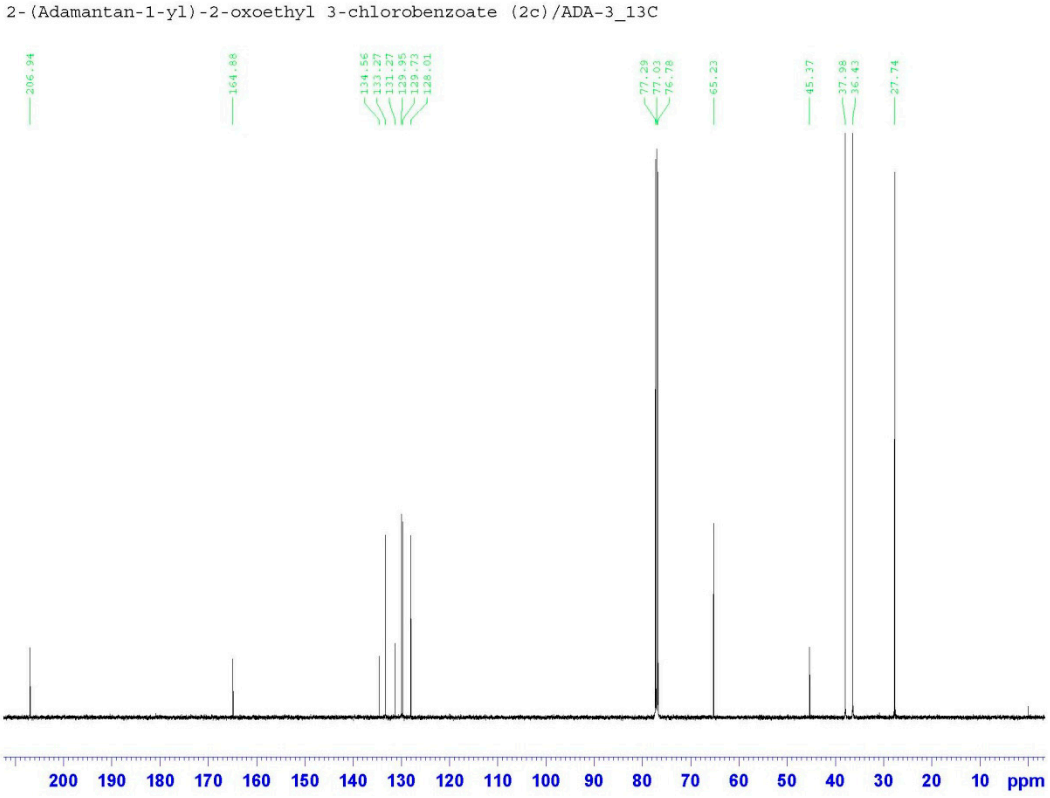

Figure S4. *Cont.*

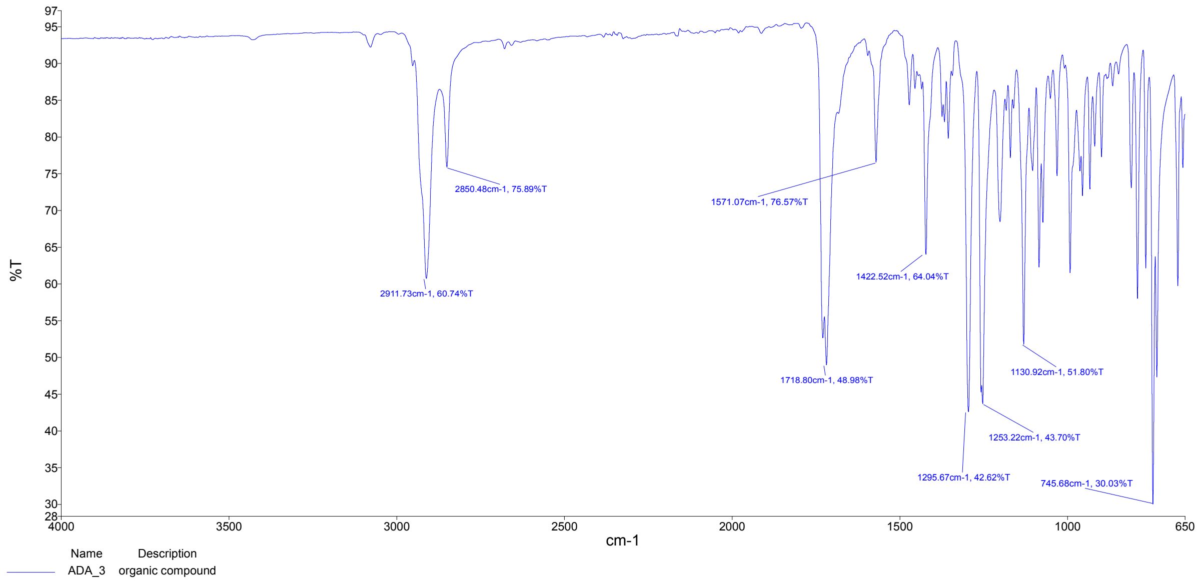

Figure S4. ^{1}H-NMR, ^{13}C-NMR and FTIR spectra of *2-(Adamantan-1-yl)-2-oxoethyl 3-chlorobenzoate* (**2c**).

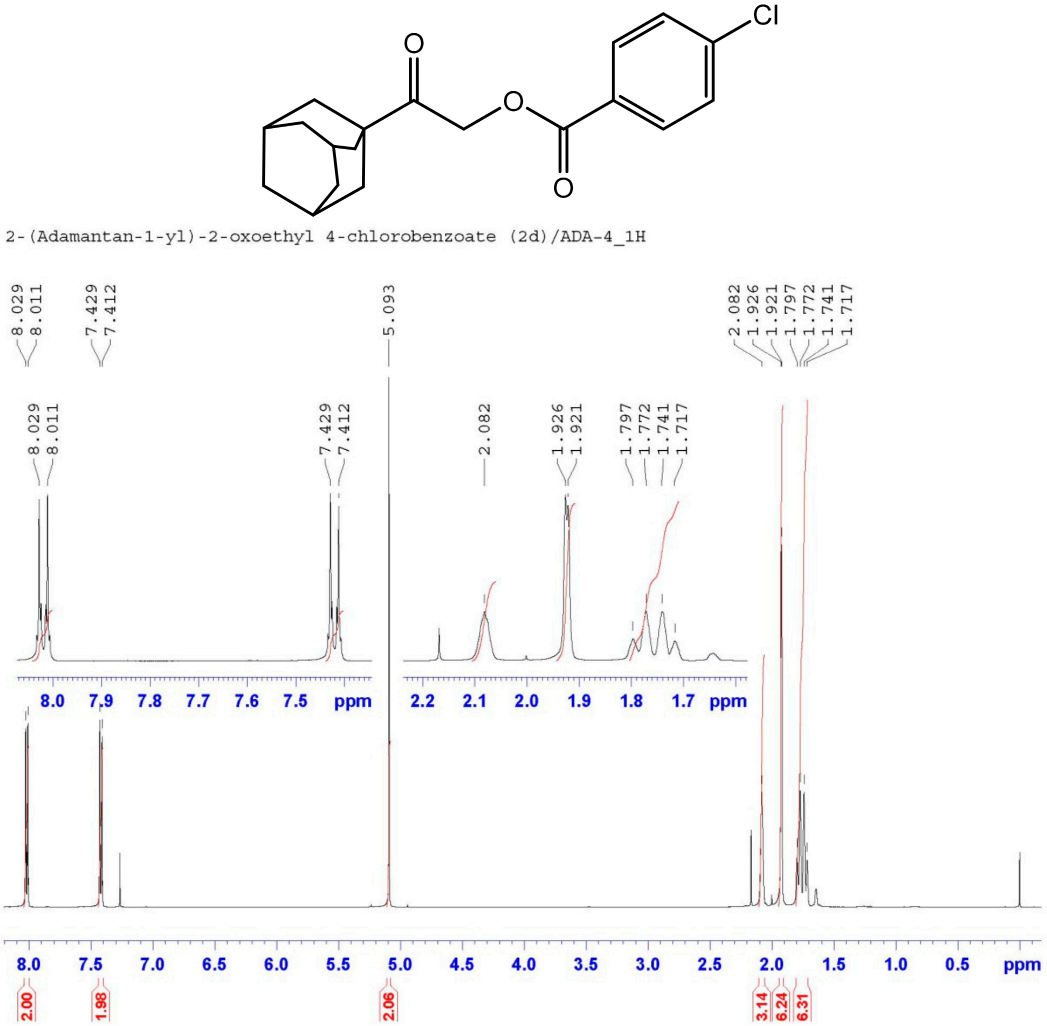

Figure S5. *Cont.*

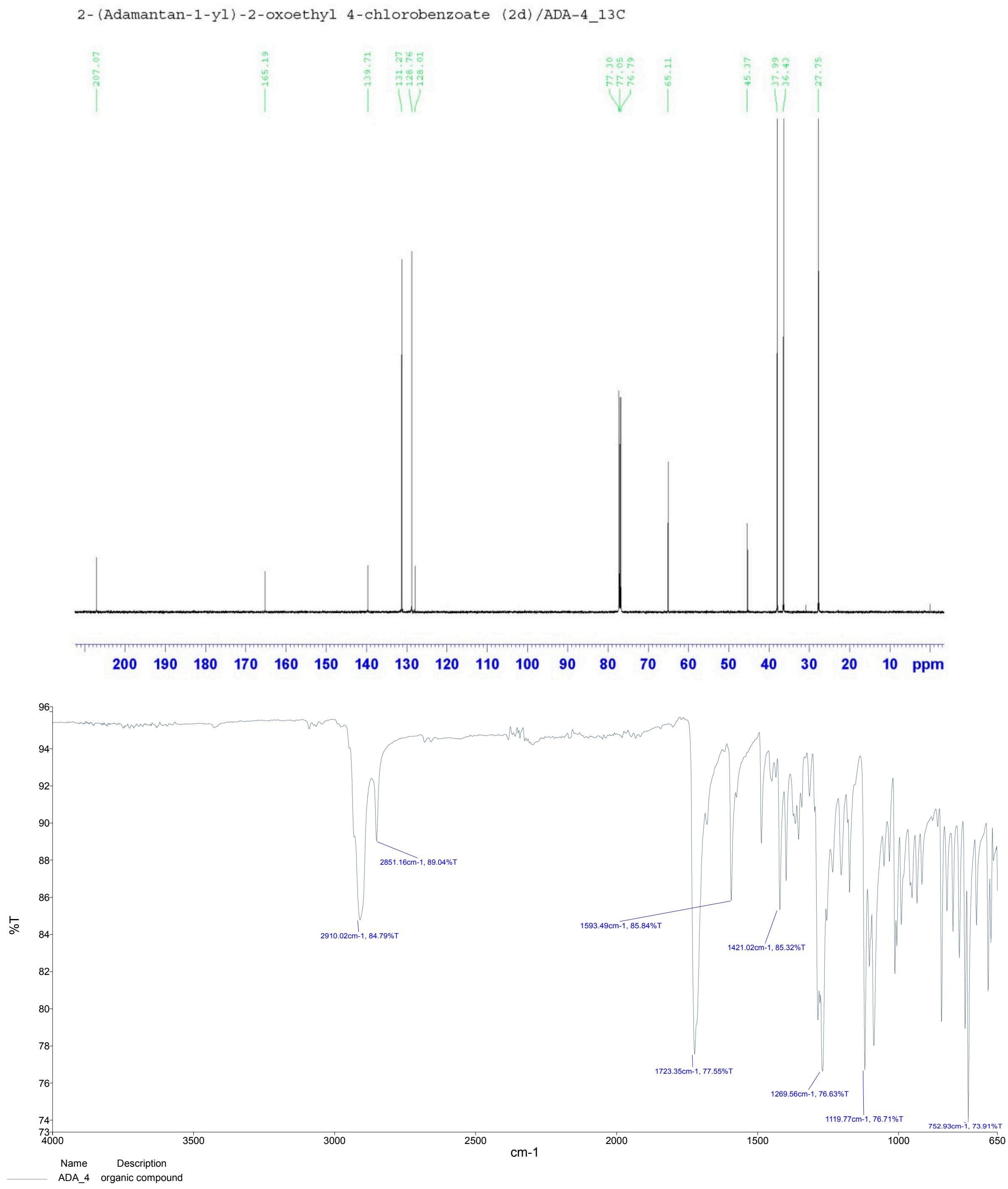

Figure S5. ^{1}H-NMR, ^{13}C-NMR and FTIR spectra of *2-(Adamantan-1-yl)-2-oxoethyl 4-chlorobenzoate* (**2d**).

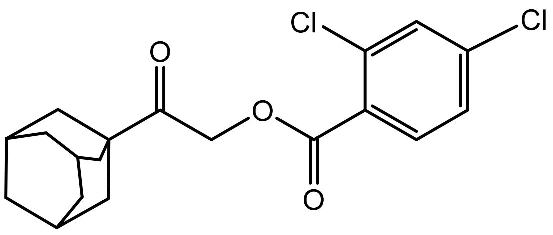

Figure S6. *Cont.*

2-(Adamantan-1-yl)-2-oxoethyl 2,4-dichlorobenzoate (2e)/ADA_5_1H

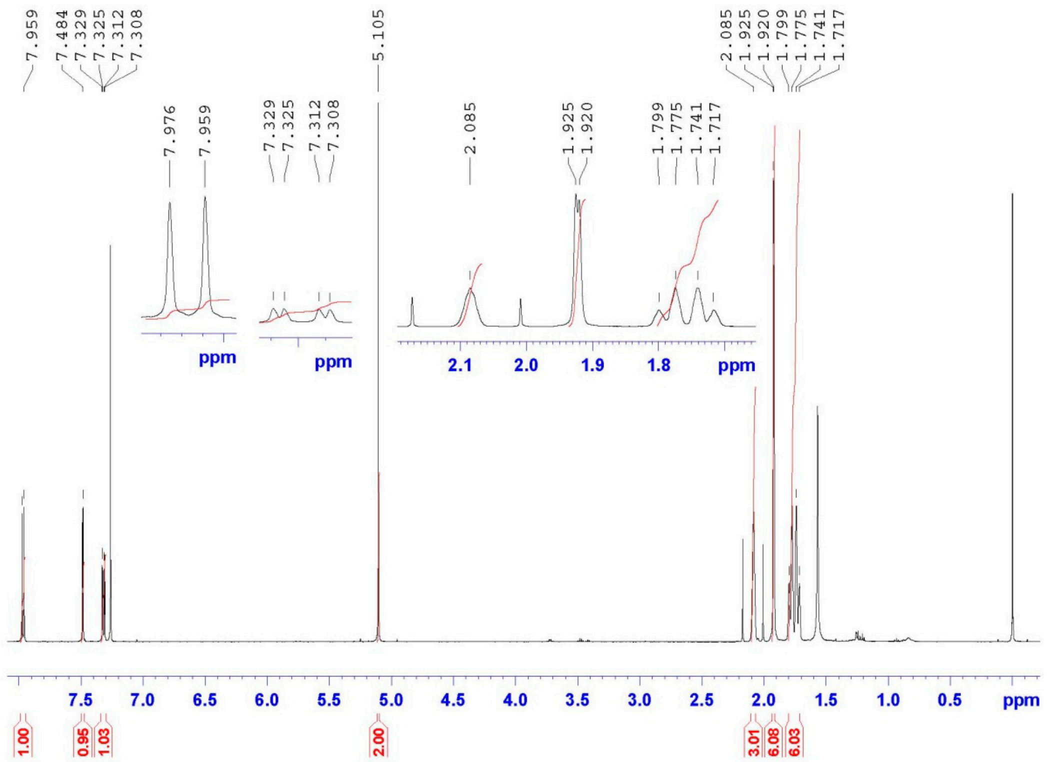

2-(Adamantan-1-yl)-2-oxoethyl 2,4-dichlorobenzoate (2e)/ADA_5_13C

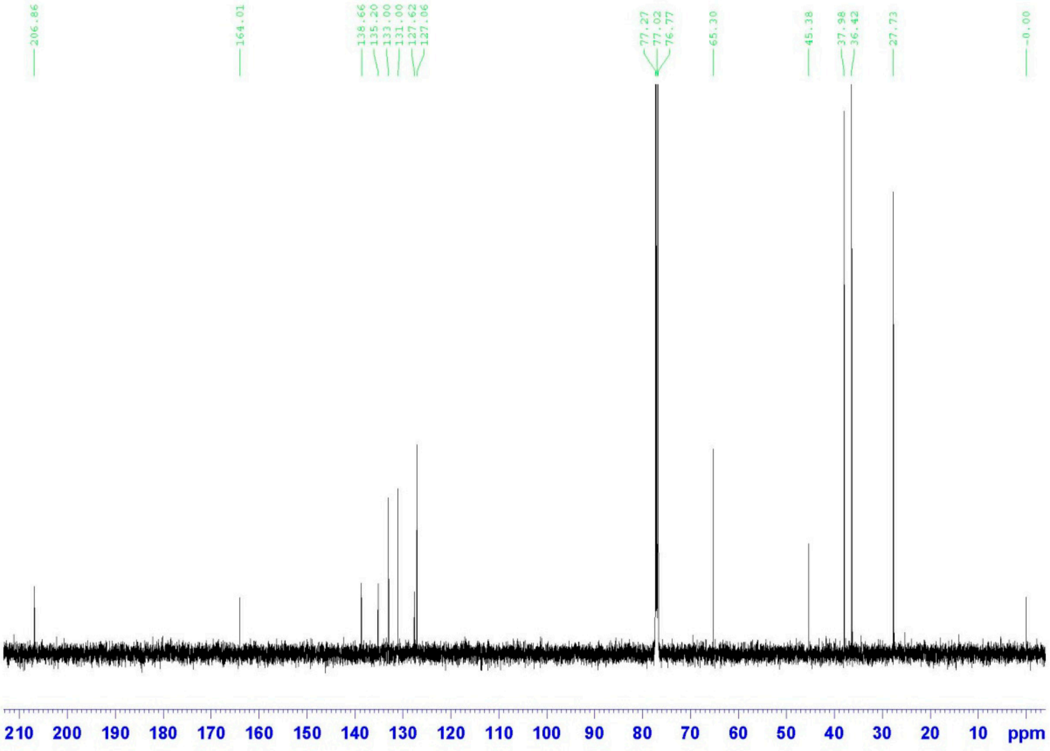

Figure S6. *Cont.*

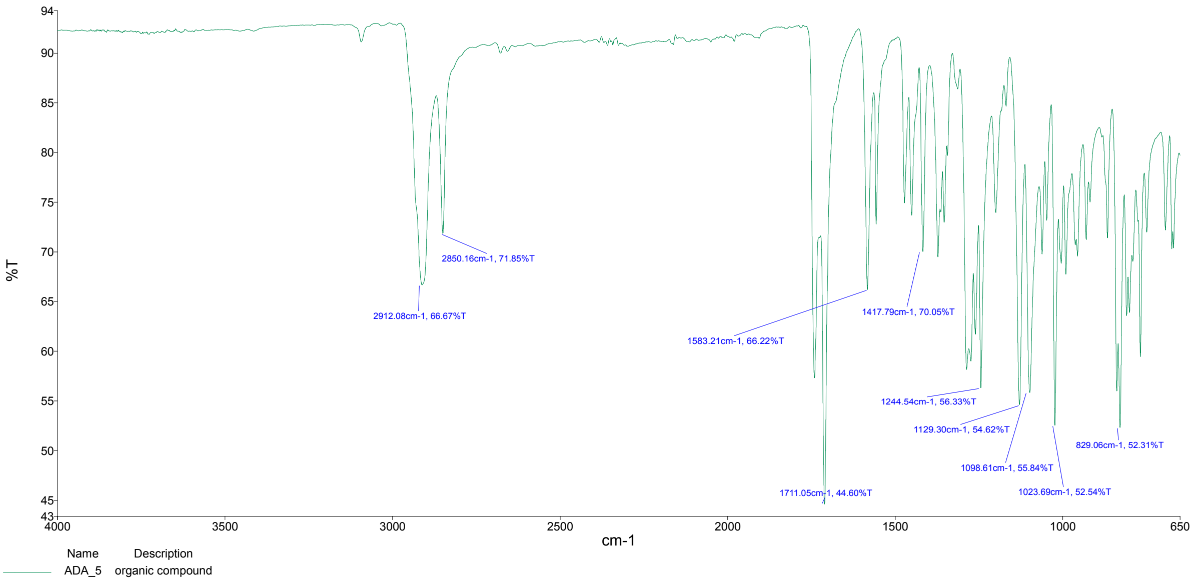

Figure S6. ^{1}H-NMR, ^{13}C-NMR and FTIR spectra of *2-(Adamantan-1-yl)-2-oxoethyl 2,4-dichlorobenzoate* (**2e**).

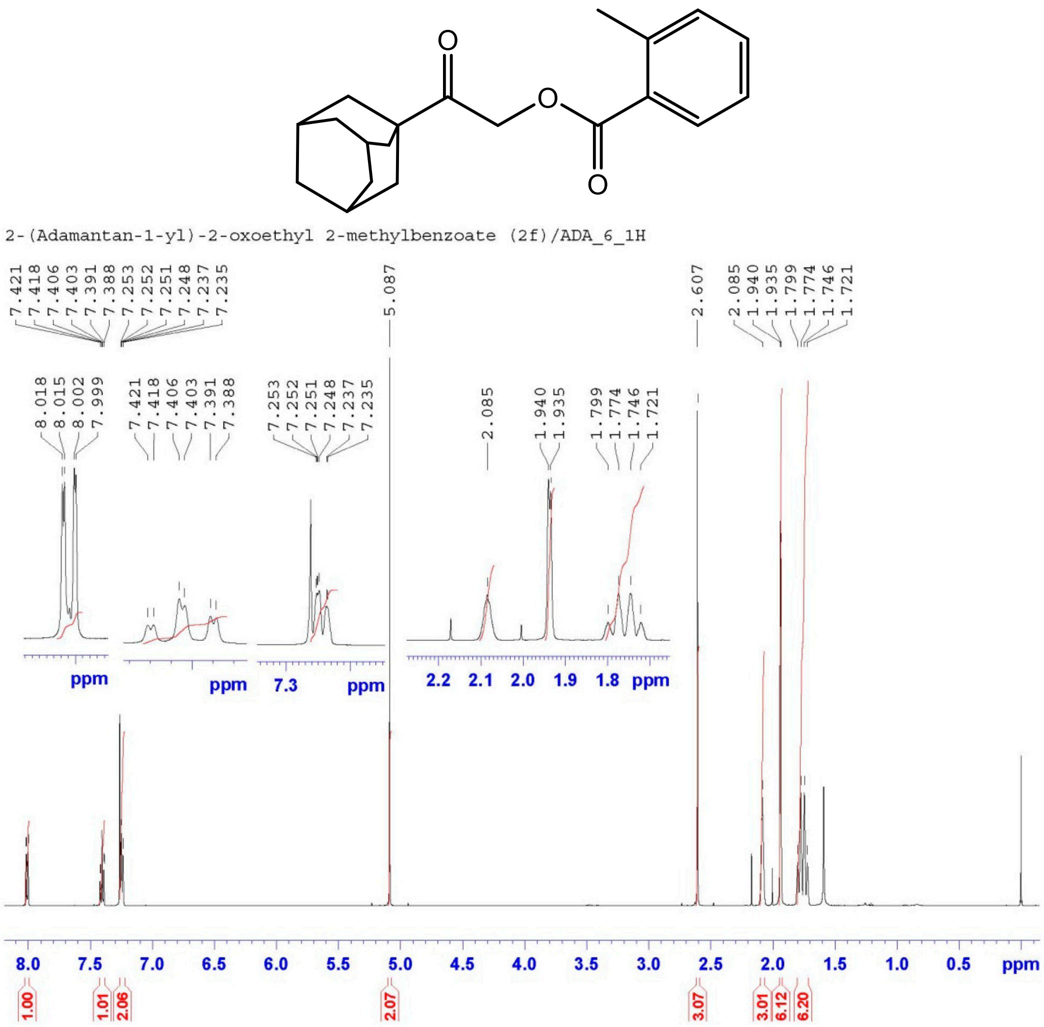

Figure S7. *Cont.*

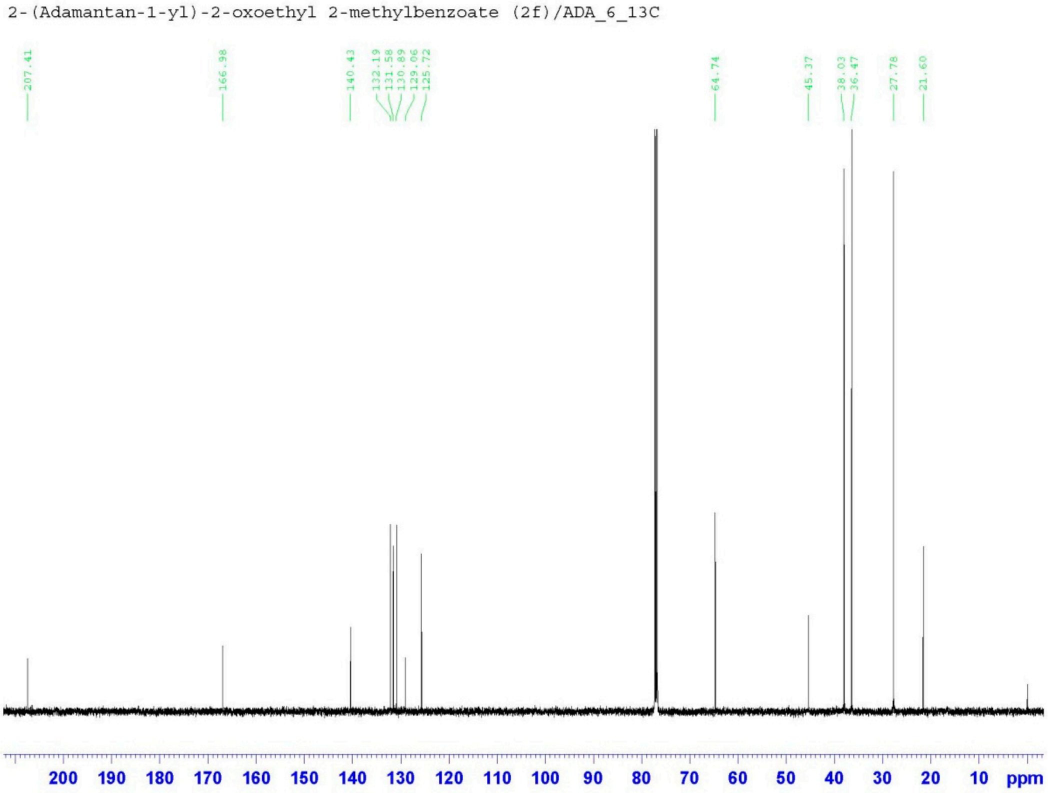

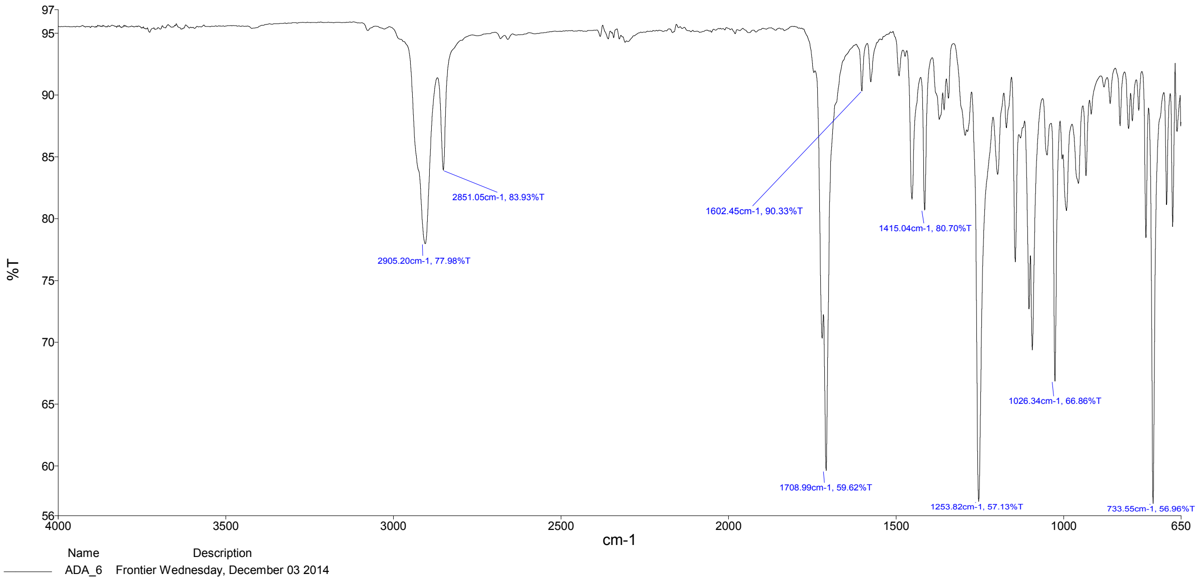

Figure S7. ^{1}H-NMR, ^{13}C-NMR and FTIR spectra of *2-(Adamantan-1-yl)-2-oxoethyl 2-methylbenzoate* (**2f**).

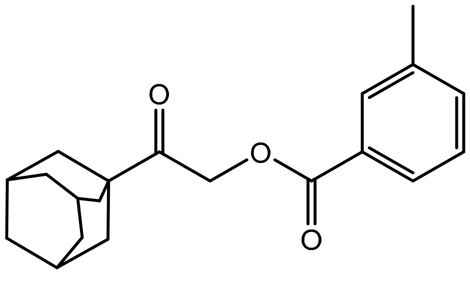

Figure S8. *Cont.*

2-(Adamantan-1-yl)-2-oxoethyl 3-methylbenzoate (2g)/ADA_7_1H

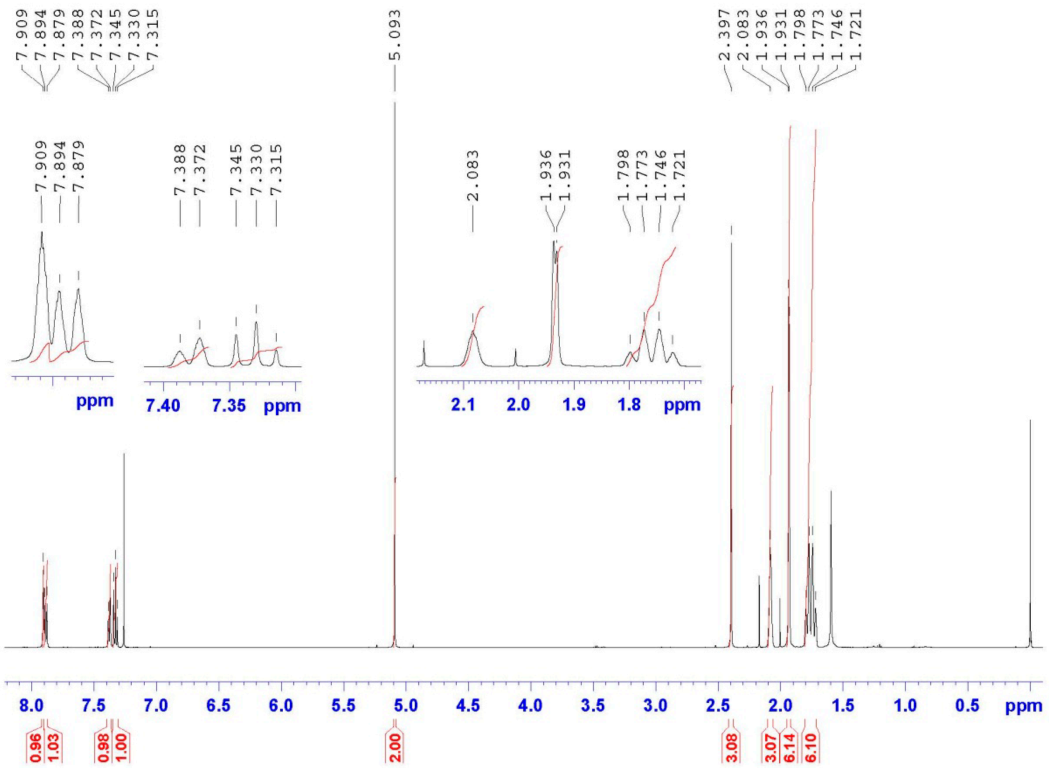

2-(Adamantan-1-yl)-2-oxoethyl 3-methylbenzoate (2g)/ADA_7_13C

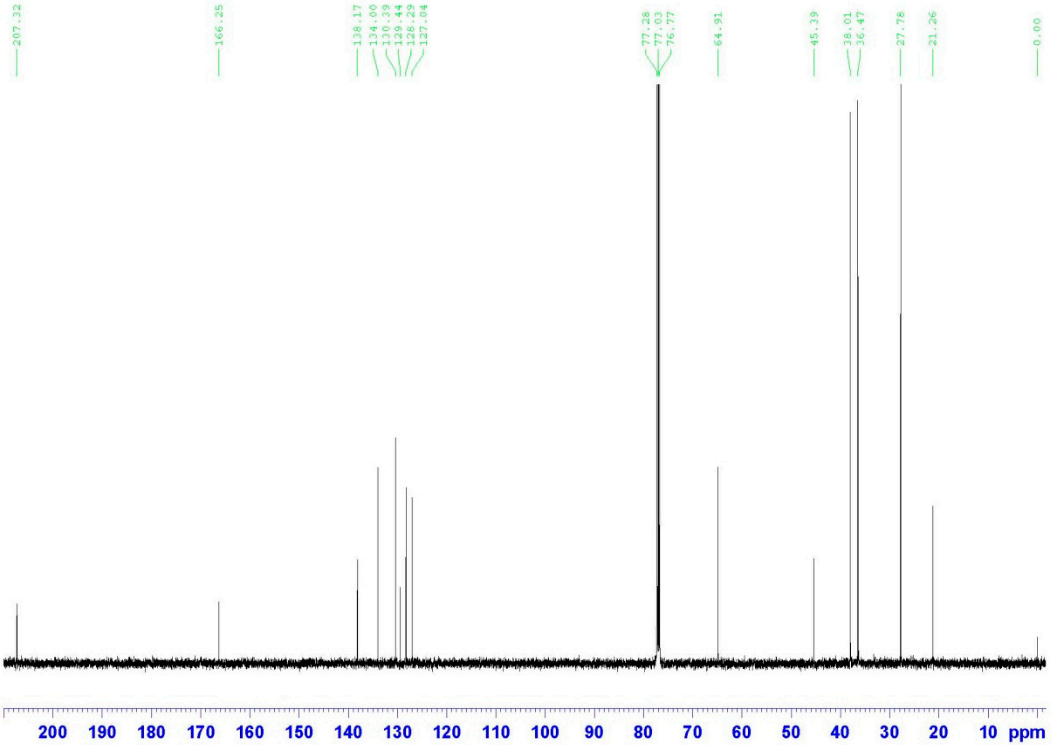

Figure S8. *Cont.*

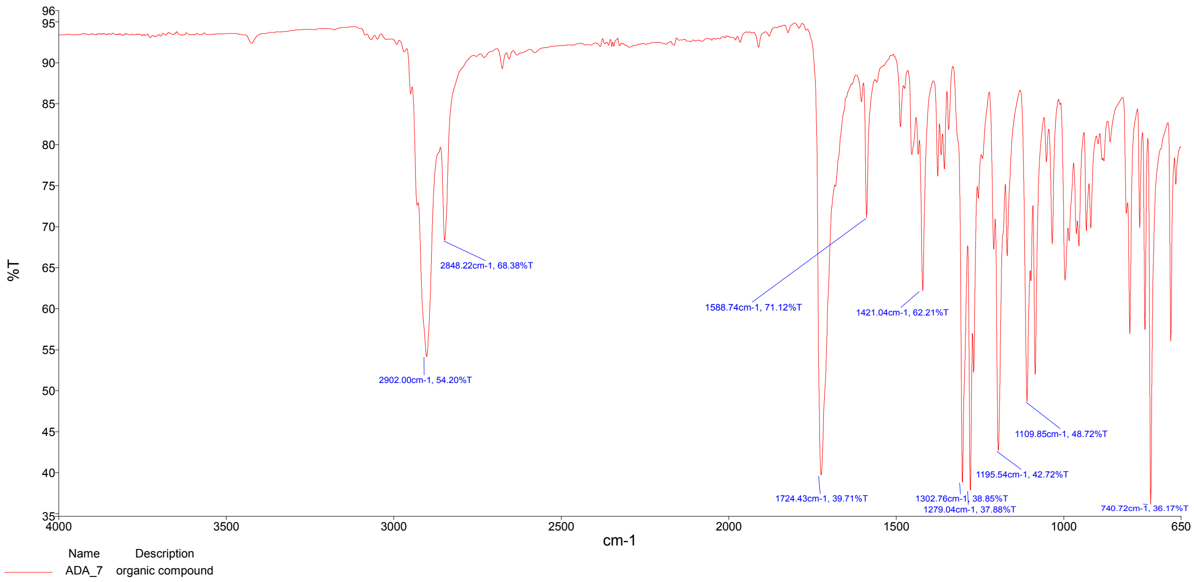

Figure S8. ^{1}H-NMR, ^{13}C-NMR and FTIR spectra of *2-(Adamantan-1-yl)-2-oxoethyl 3-methylbenzoate* (**2g**).

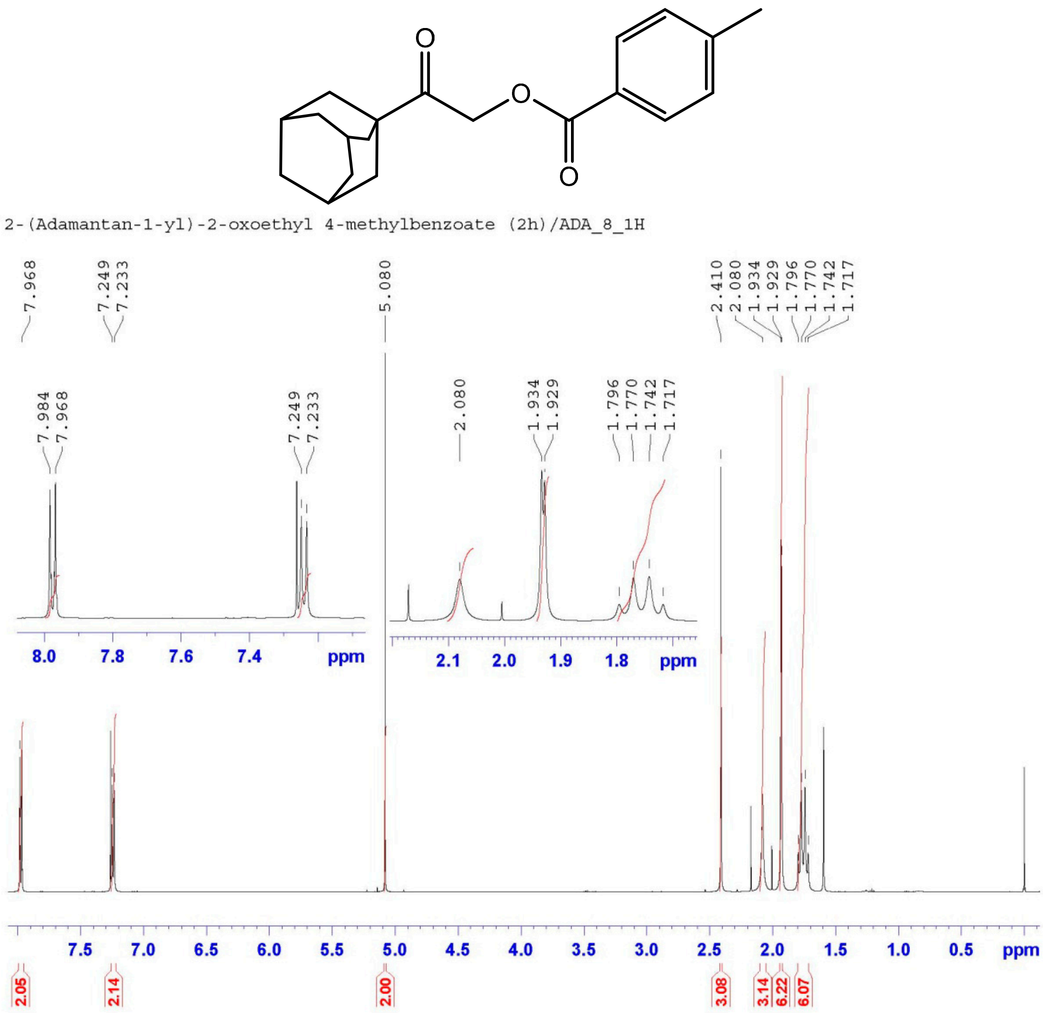

Figure S9. *Cont.*

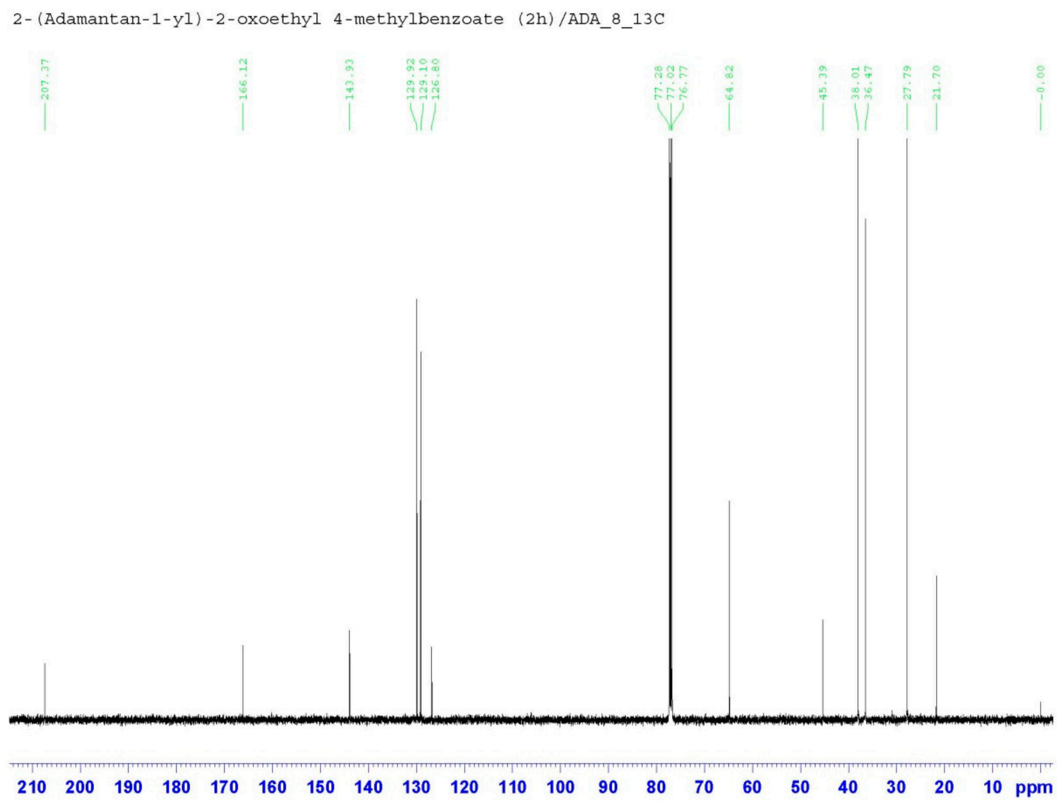

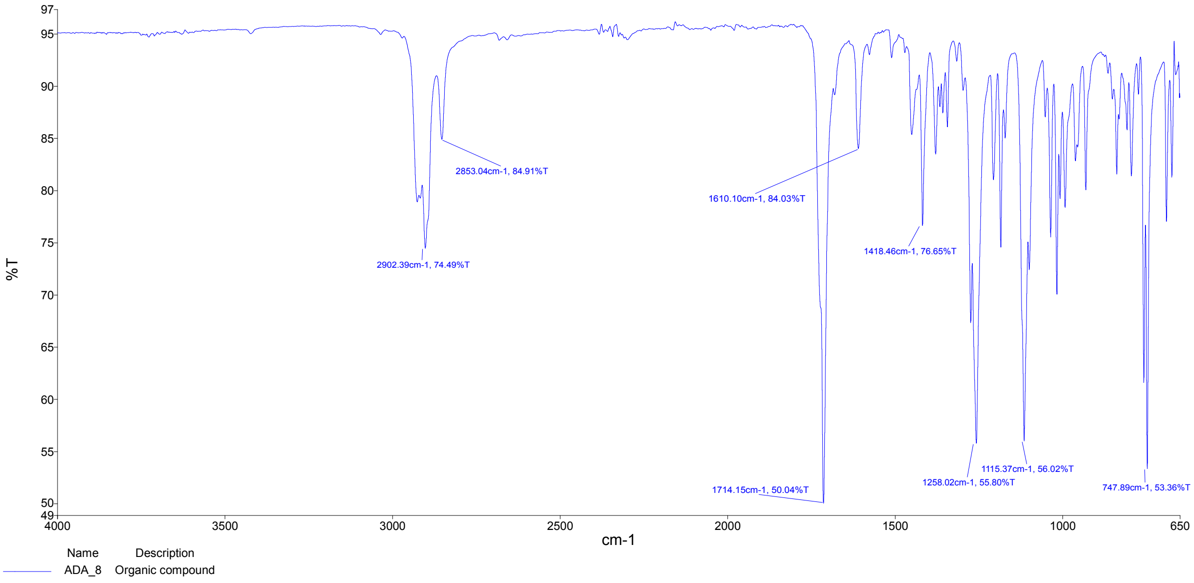

Figure S9. ^{1}H-NMR, ^{13}C-NMR and FTIR spectra of *2-(Adamantan-1-yl)-2-oxoethyl 4-methylbenzoate* (**2h**).

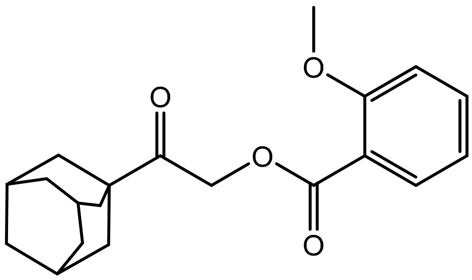

Figure S10. *Cont.*

2-(Adamantan-1-yl)-2-oxoethyl 2-methoxybenzoate (2i)/ADA_9_1H

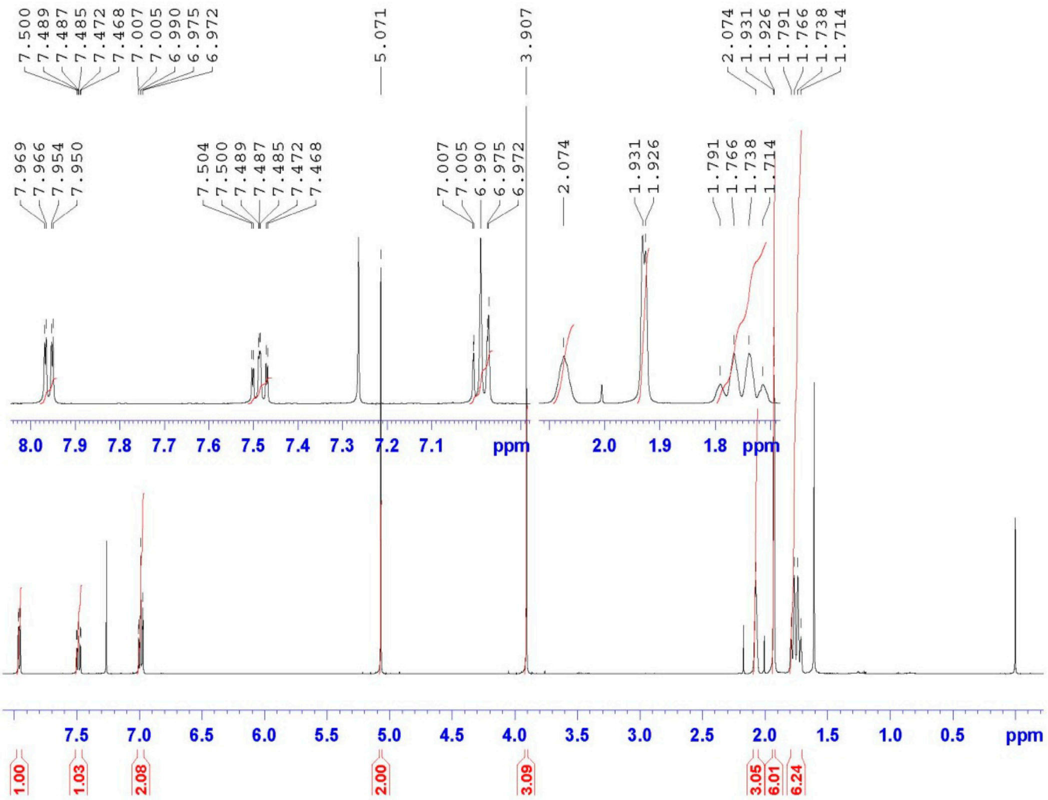

2-(Adamantan-1-yl)-2-oxoethyl 2-methoxybenzoate (2i)/ADA_9_13C

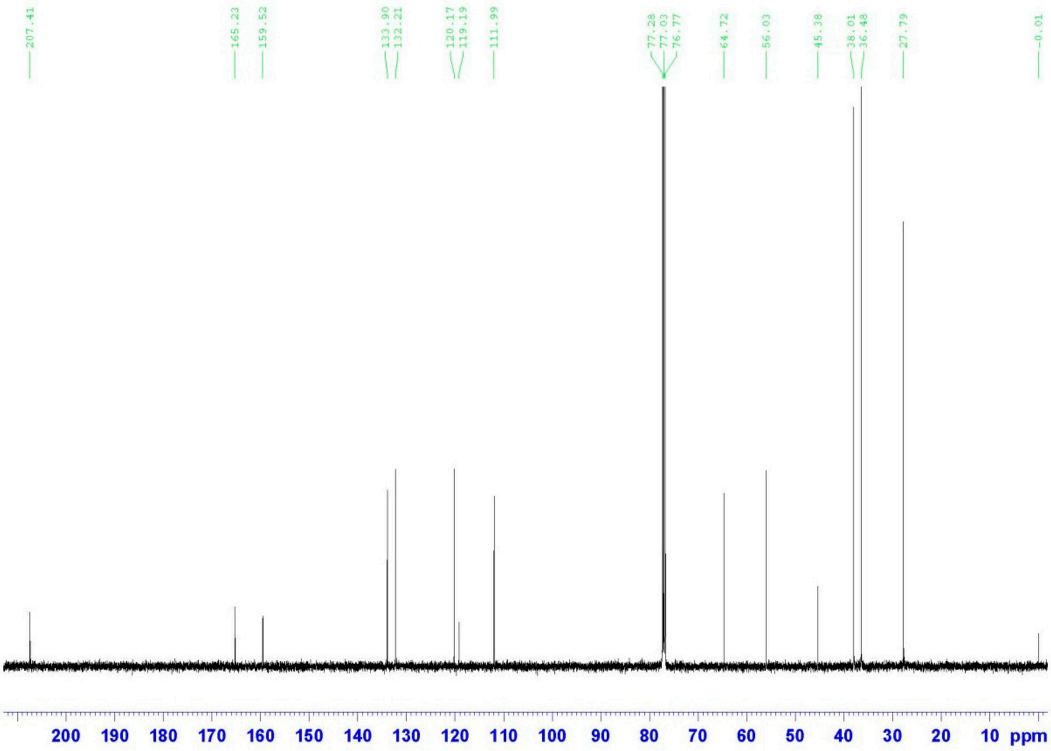

Figure S10. *Cont.*

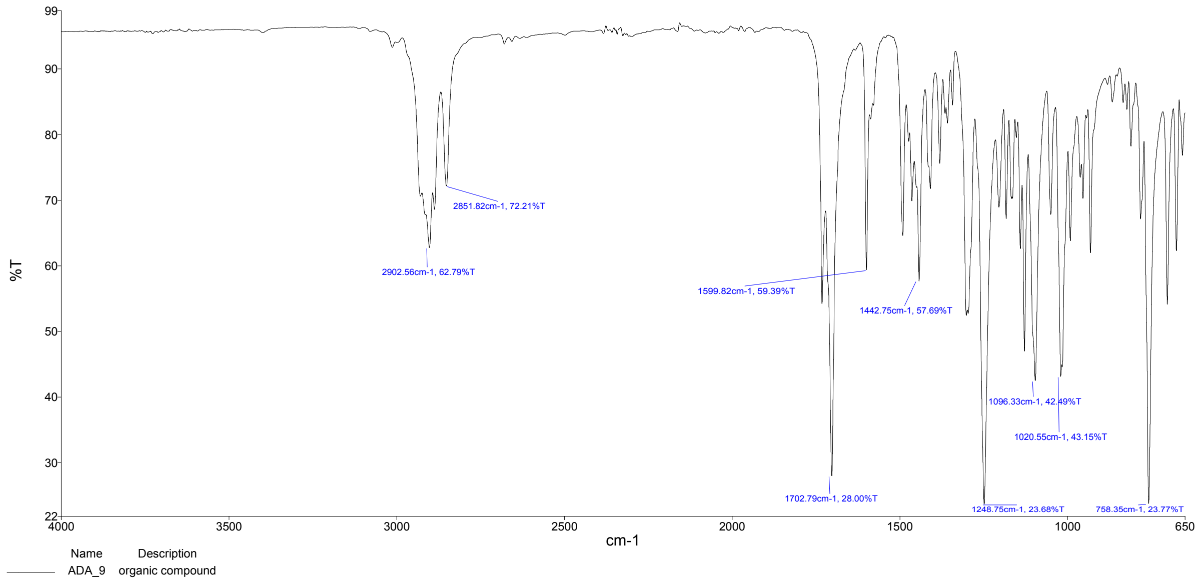

Figure S10. ^{1}H-NMR, ^{13}C-NMR and FTIR spectra of *2-(Adamantan-1-yl)-2-oxoethyl 2-methoxybenzoate* (**2i**).

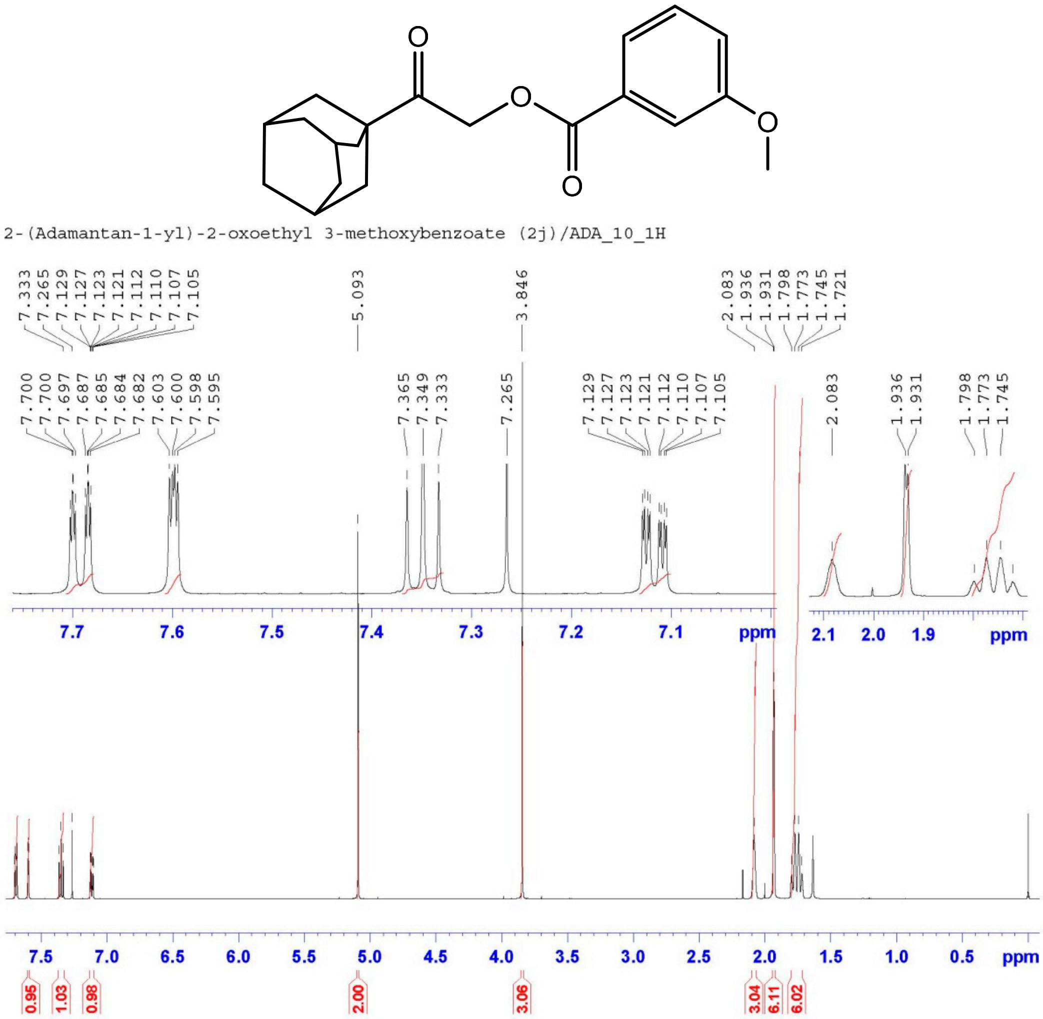

Figure S11. *Cont.*

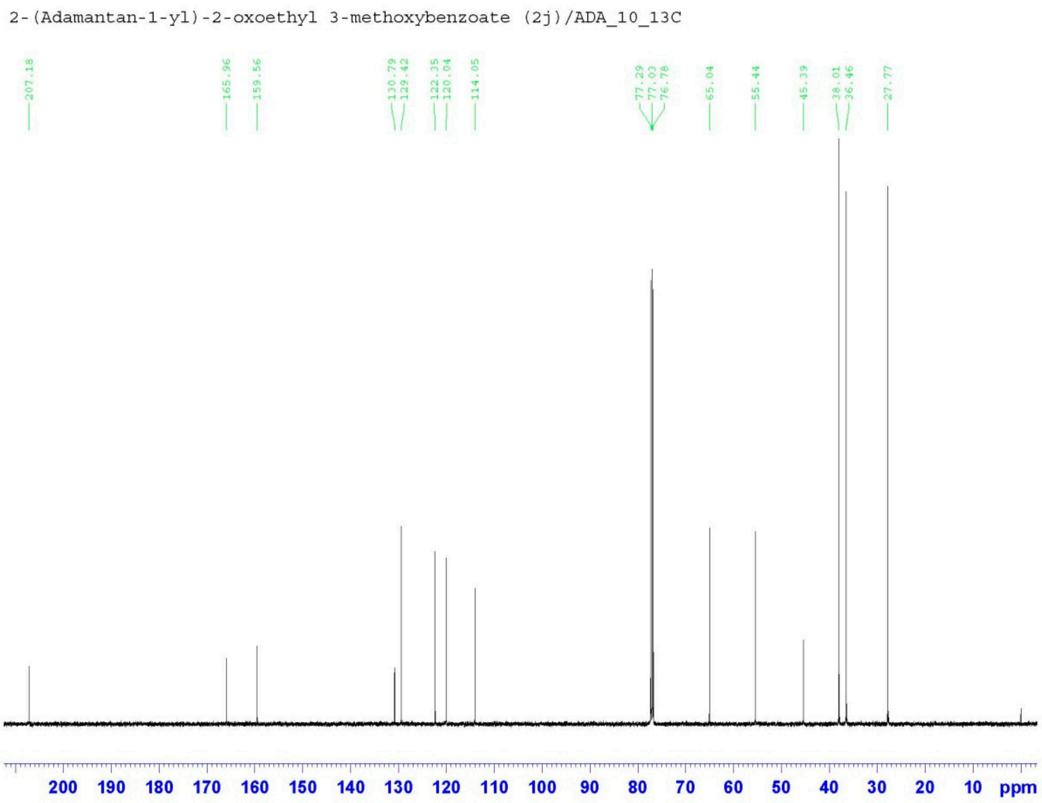

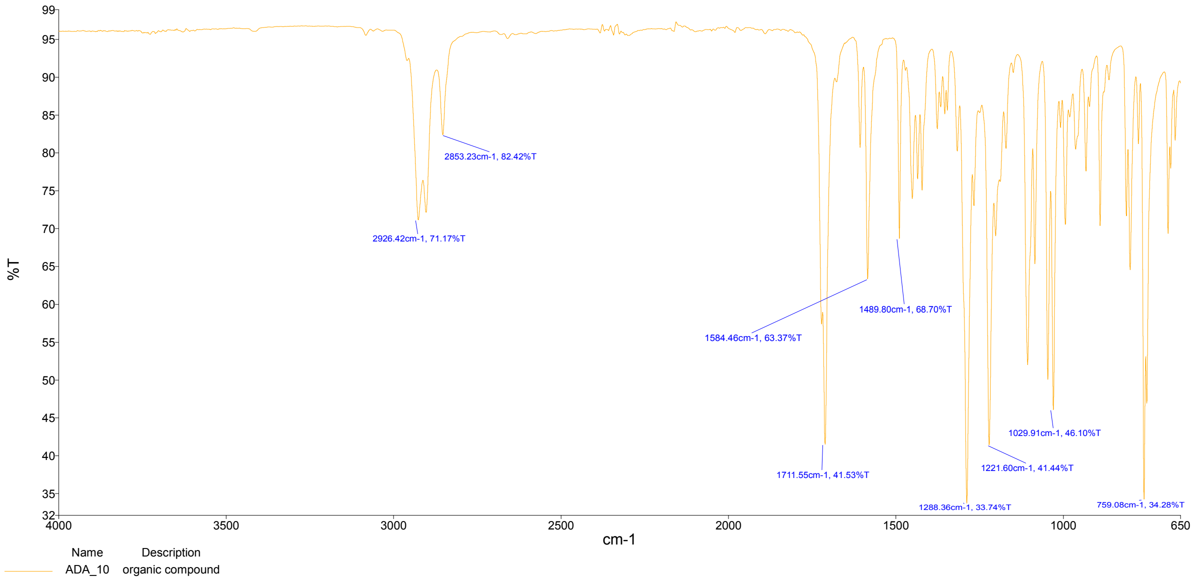

Figure S11. ^{1}H-NMR, ^{13}C-NMR and FTIR spectra of *2-(Adamantan-1-yl)-2-oxoethyl 3-methoxybenzoate* (**2j**).

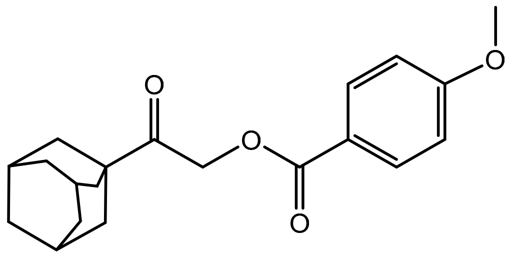

Figure S12. *Cont.*

2-(Adamantan-1-yl)-2-oxoethyl 4-methoxybenzoate (2k)/ADA_11_1H

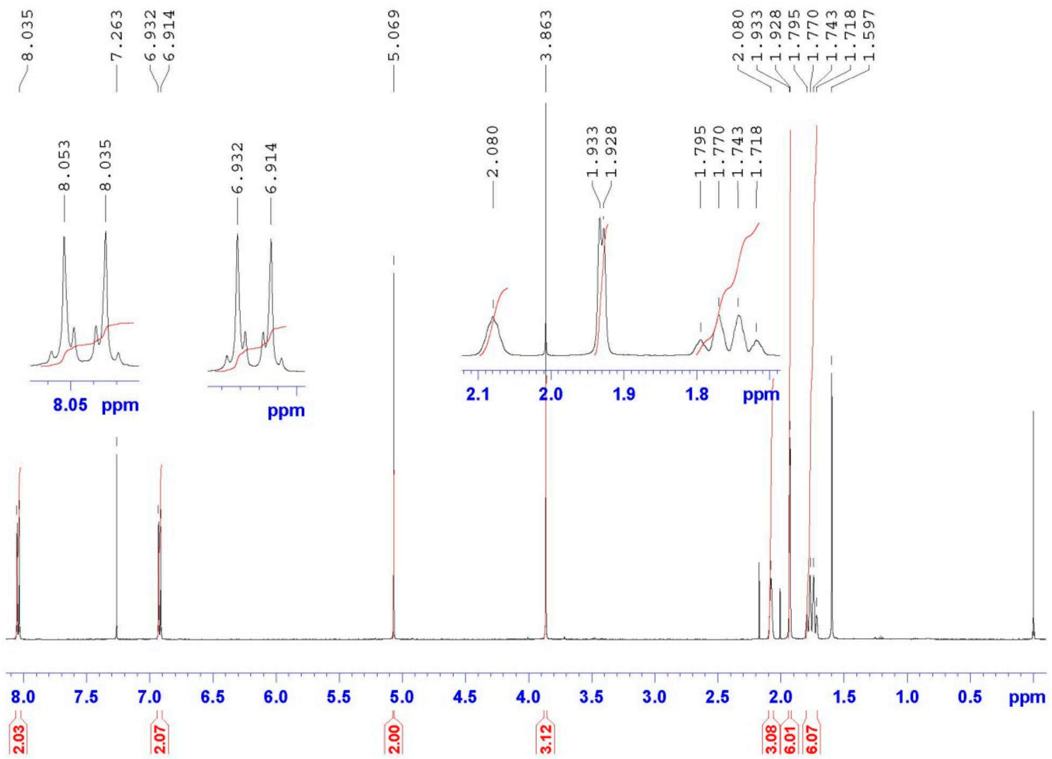

2-(Adamantan-1-yl)-2-oxoethyl 4-methoxybenzoate (2k)/ADA_11_13C

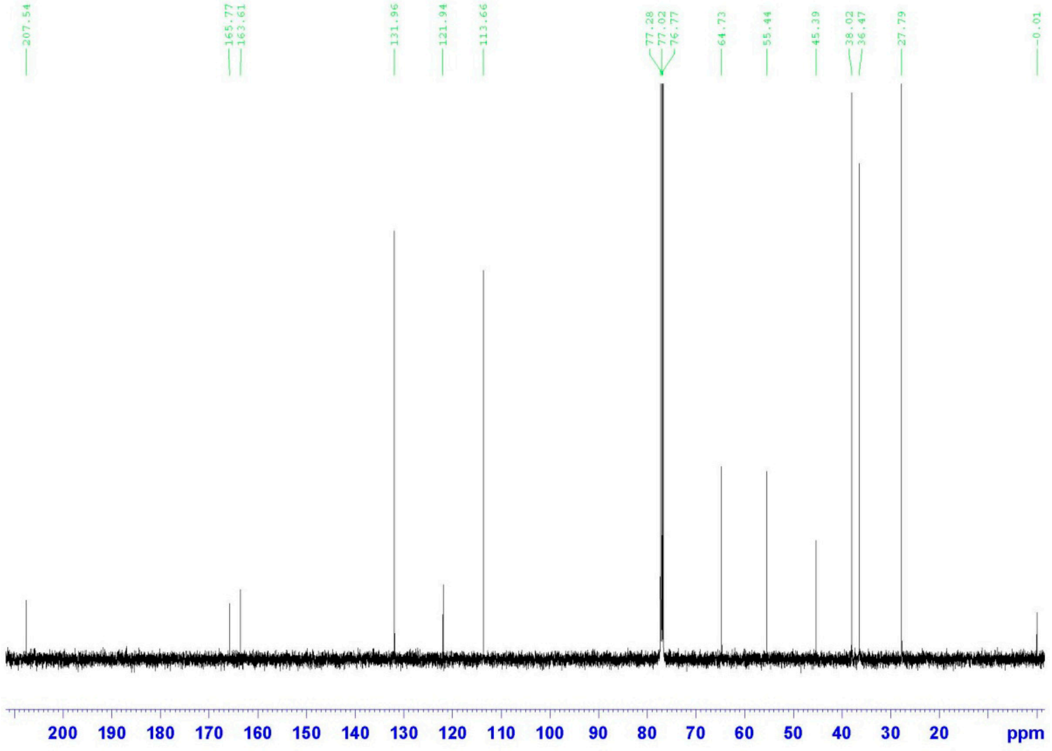

Figure S12. *Cont.*

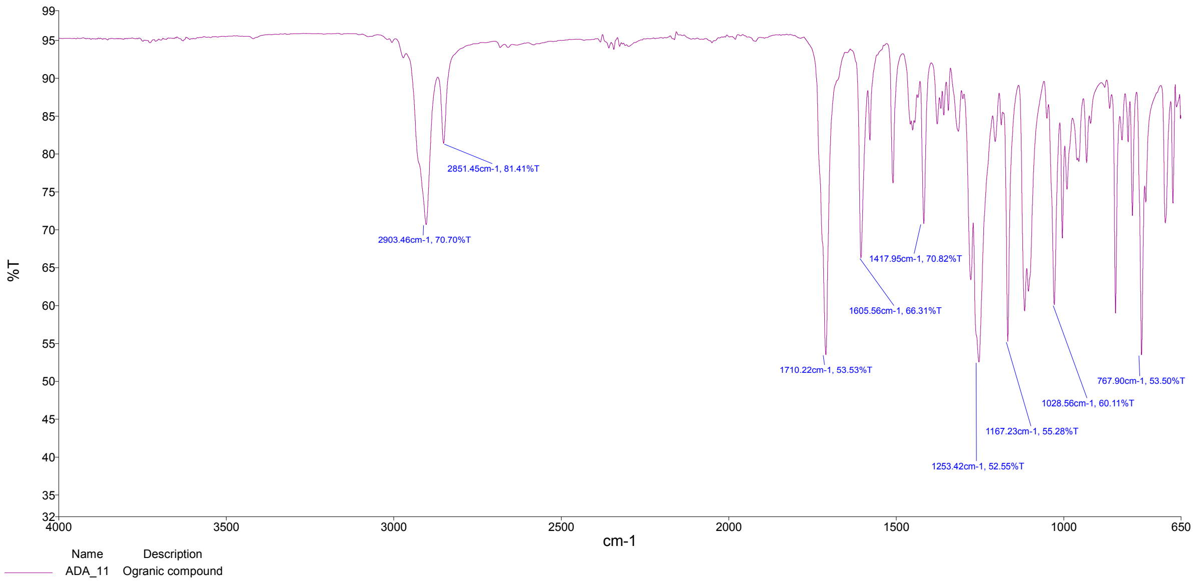

Figure S12. ^{1}H-NMR, ^{13}C-NMR and FTIR spectra of *2-(Adamantan-1-yl)-2-oxoethyl 4-methoxybenzoate* (**2k**).

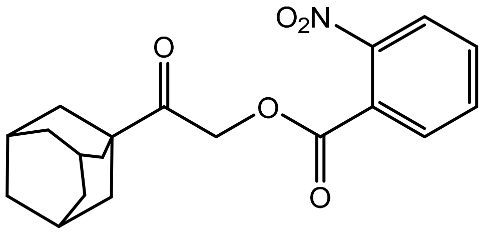

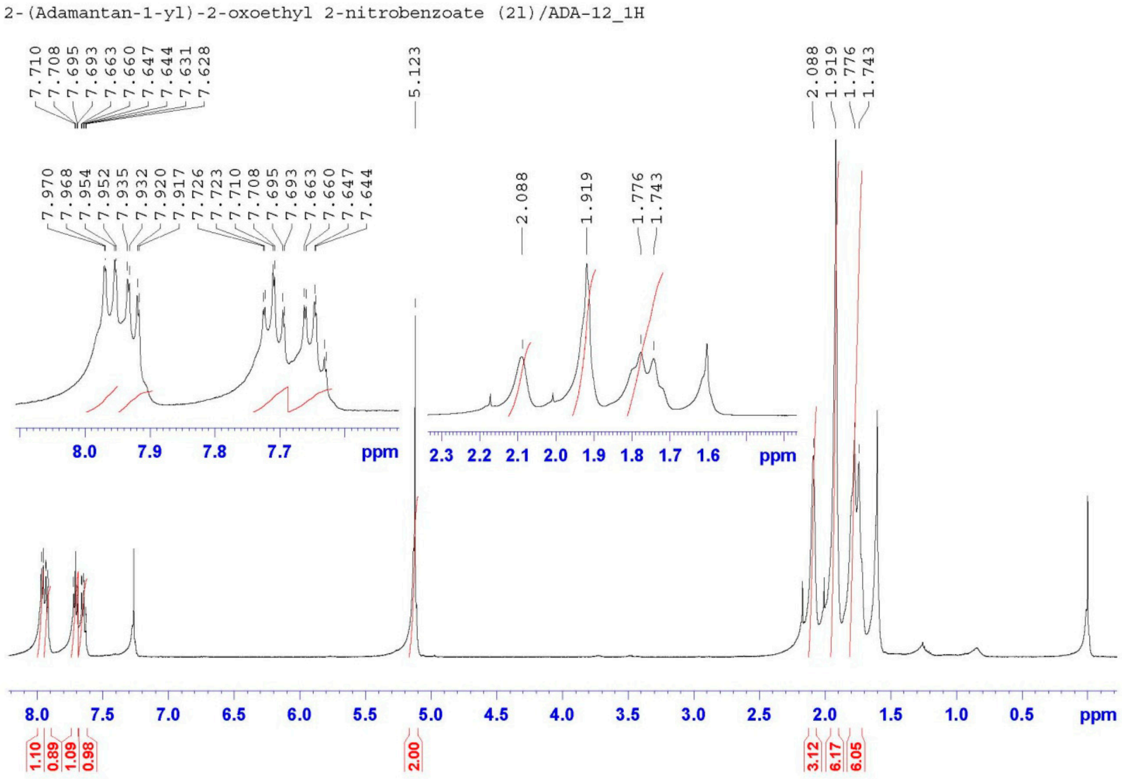

Figure S13. *Cont.*

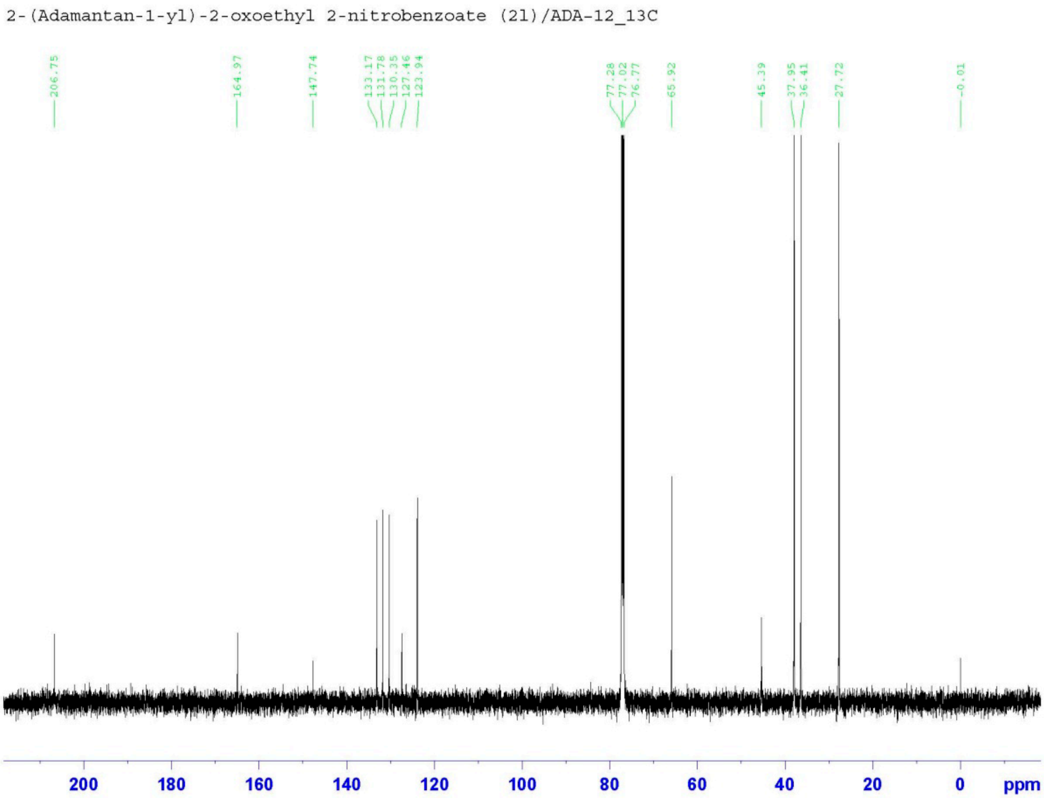

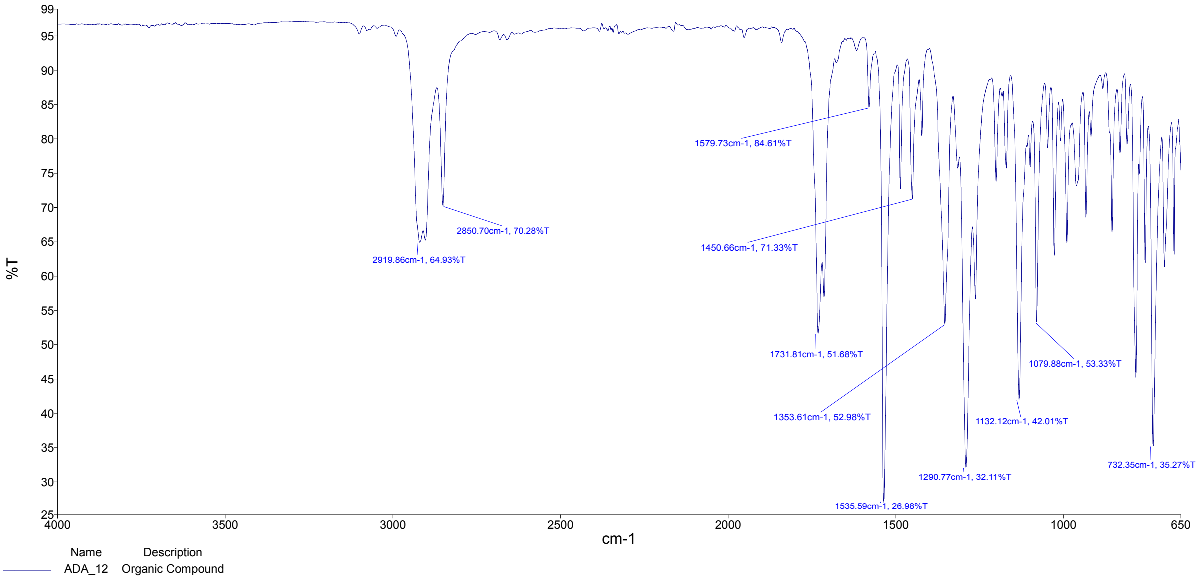

Figure S13. ^{1}H-NMR, ^{13}C-NMR and FTIR spectra of *2-(Adamantan-1-yl)-2-oxoethyl 2-nitrobenzoate* (**2l**).

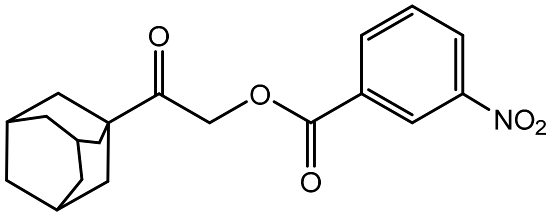

Figure S14. *Cont.*

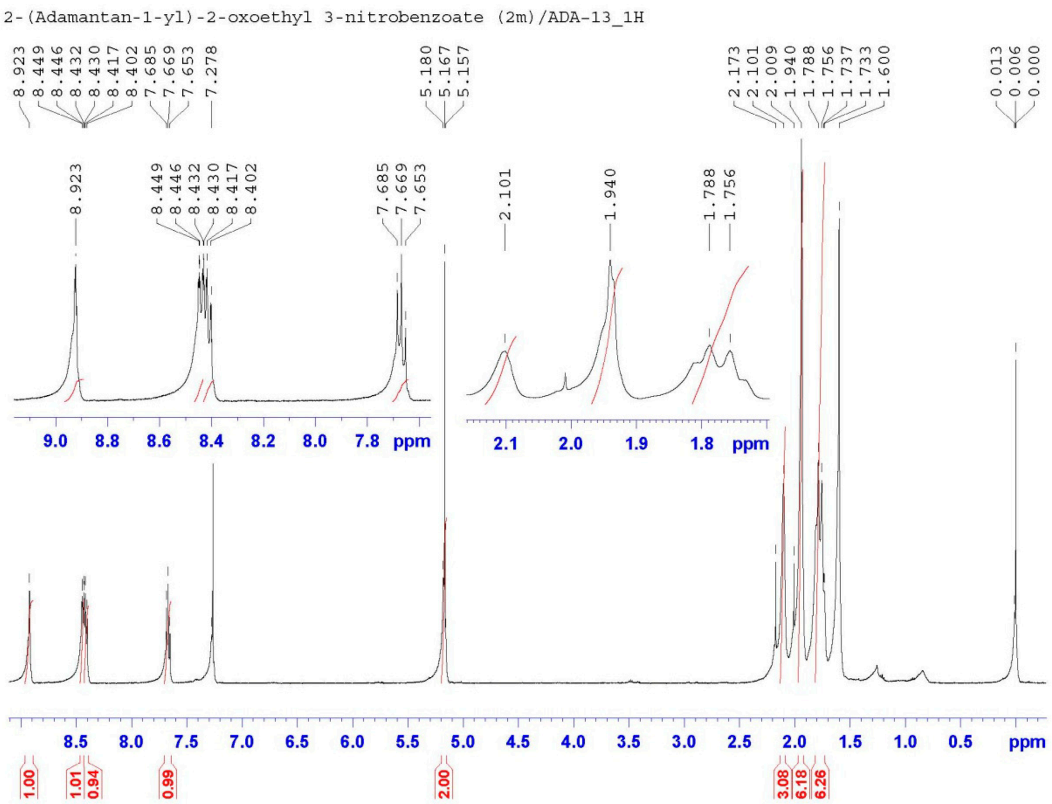

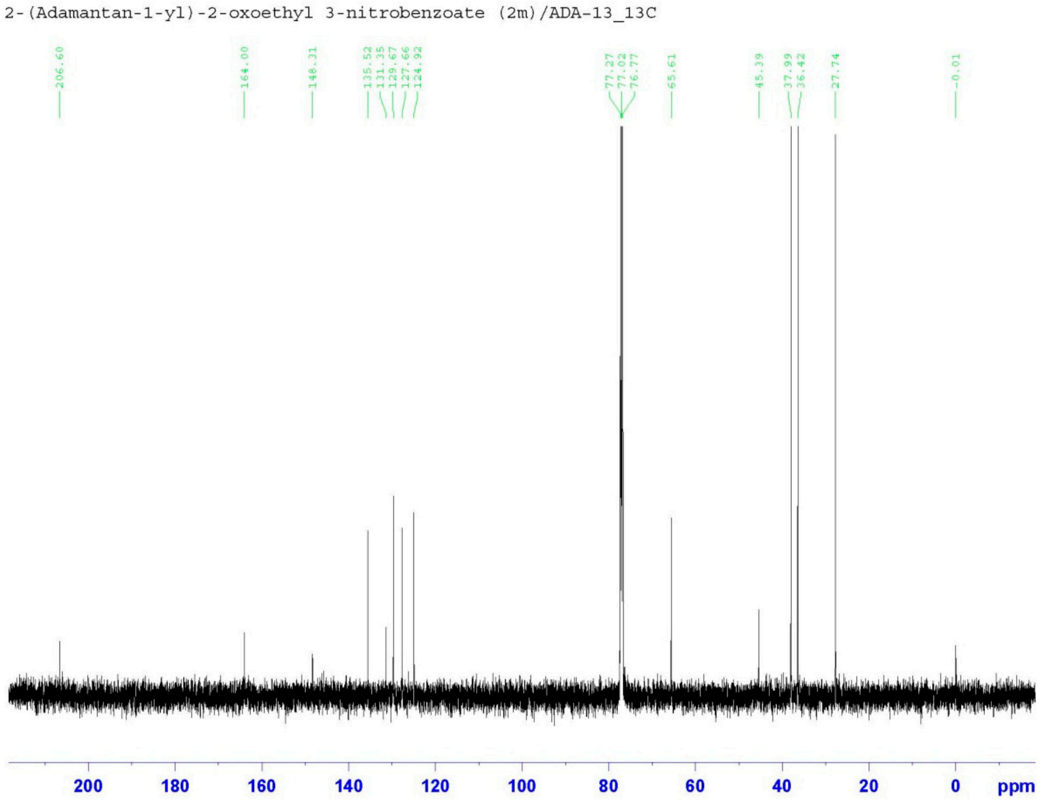

Figure S14. *Cont.*

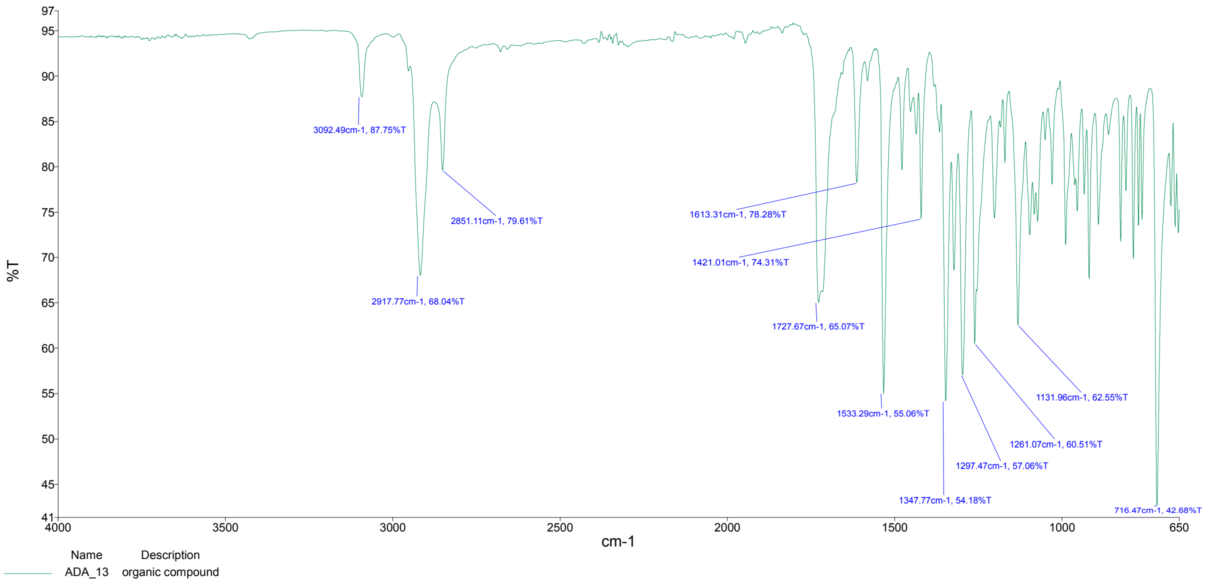

Figure S14. ^{1}H-NMR, ^{13}C-NMR and FTIR spectra of *2-(Adamantan-1-yl)-2-oxoethyl 3-nitrobenzoate* (**2m**).

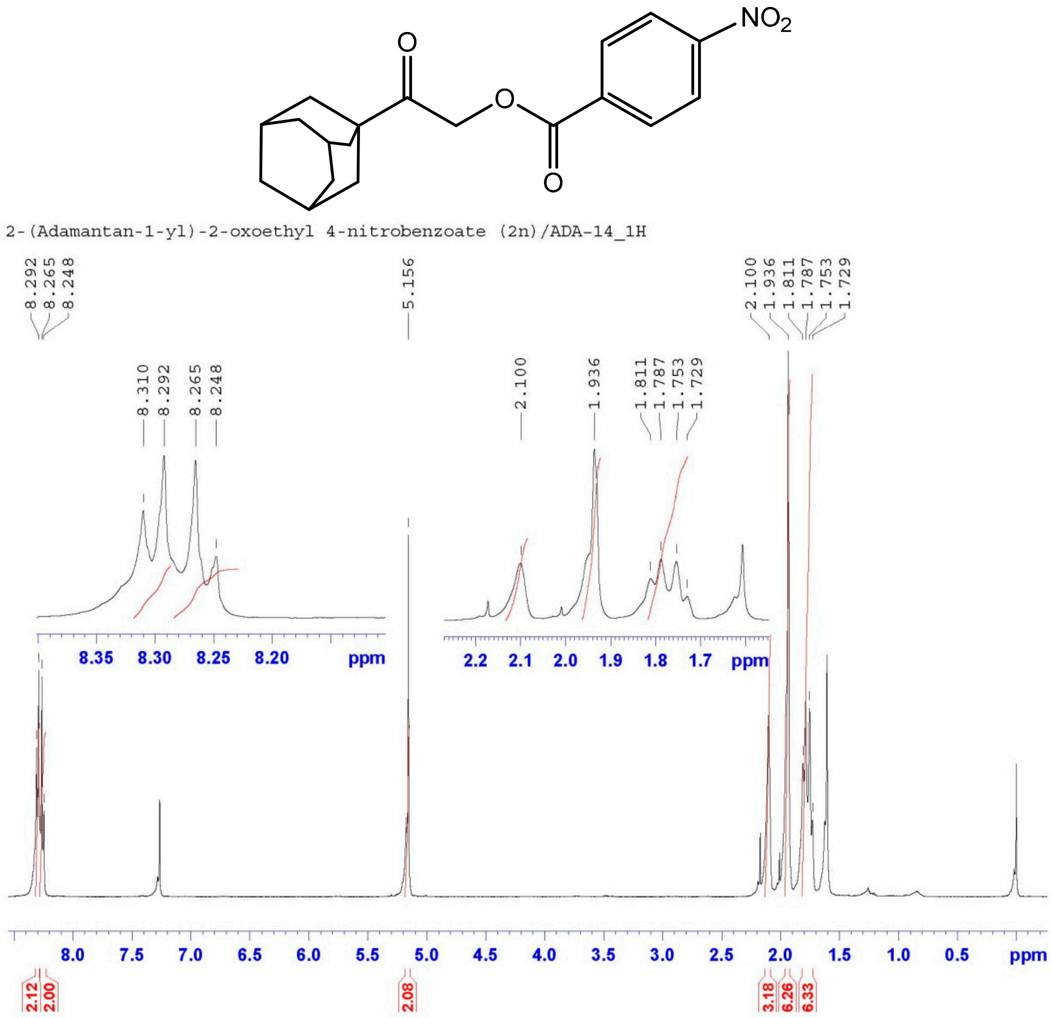

Figure S15. *Cont.*

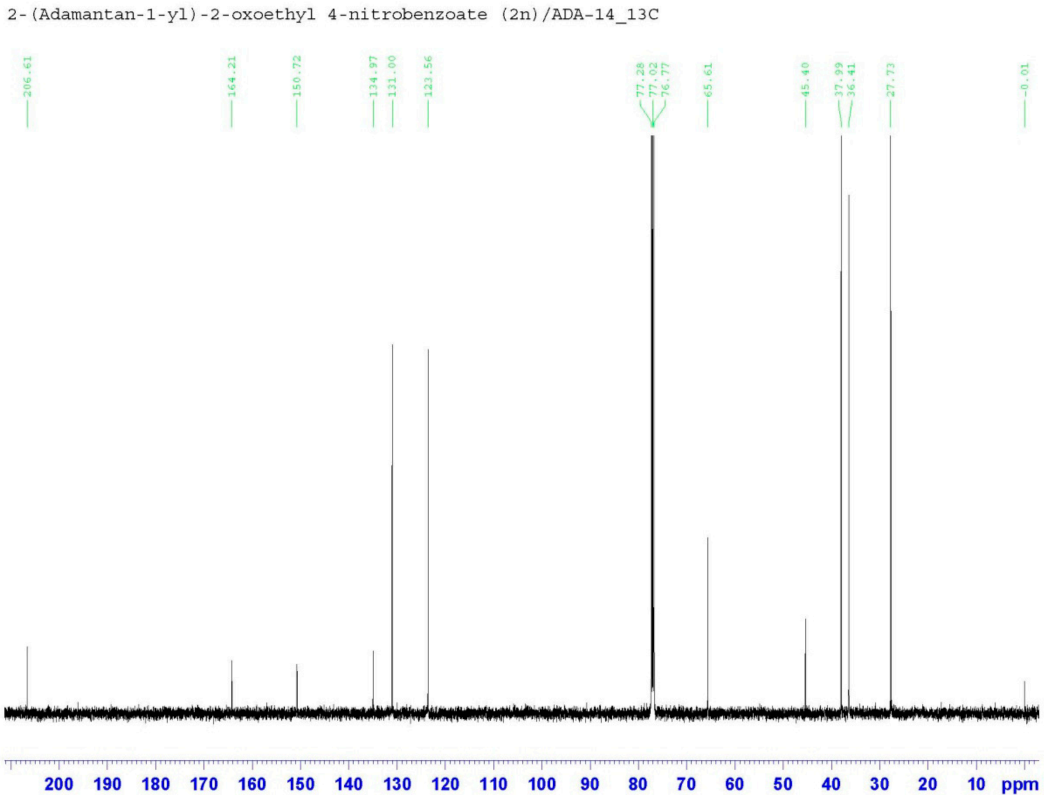

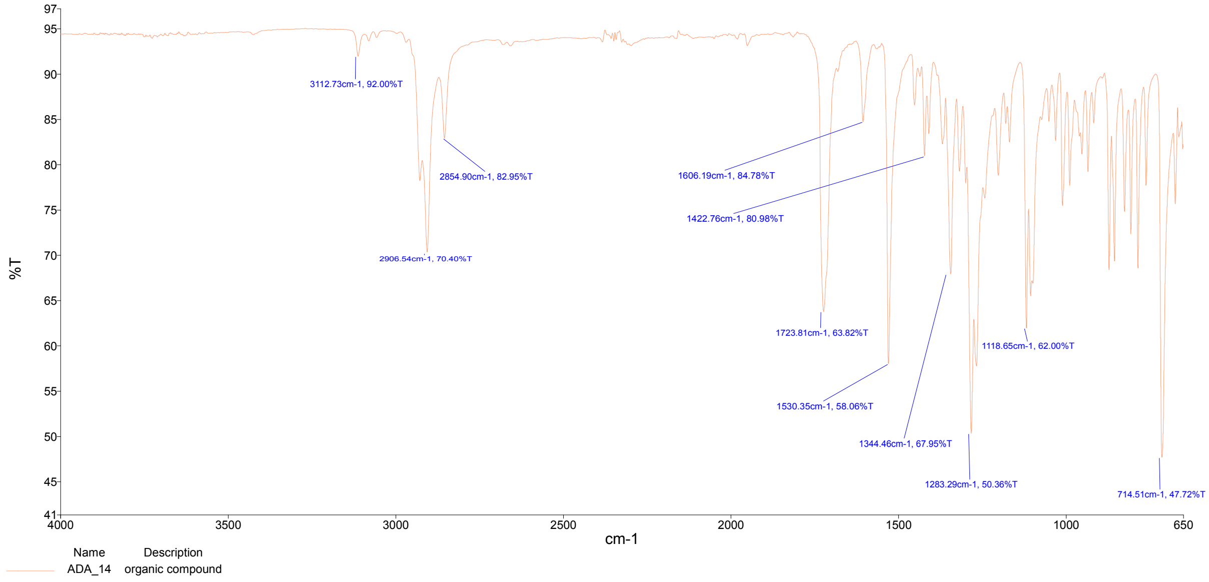

Figure S15. ^{1}H-NMR, ^{13}C-NMR and FTIR spectra of *2-(Adamantan-1-yl)-2-oxoethyl 4-nitrobenzoate* (**2n**).

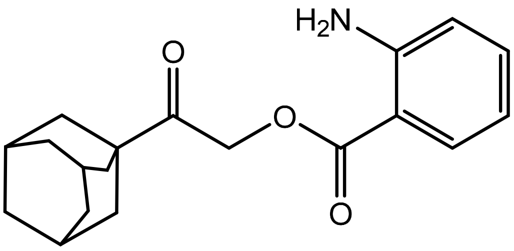

Figure S16. *Cont.*

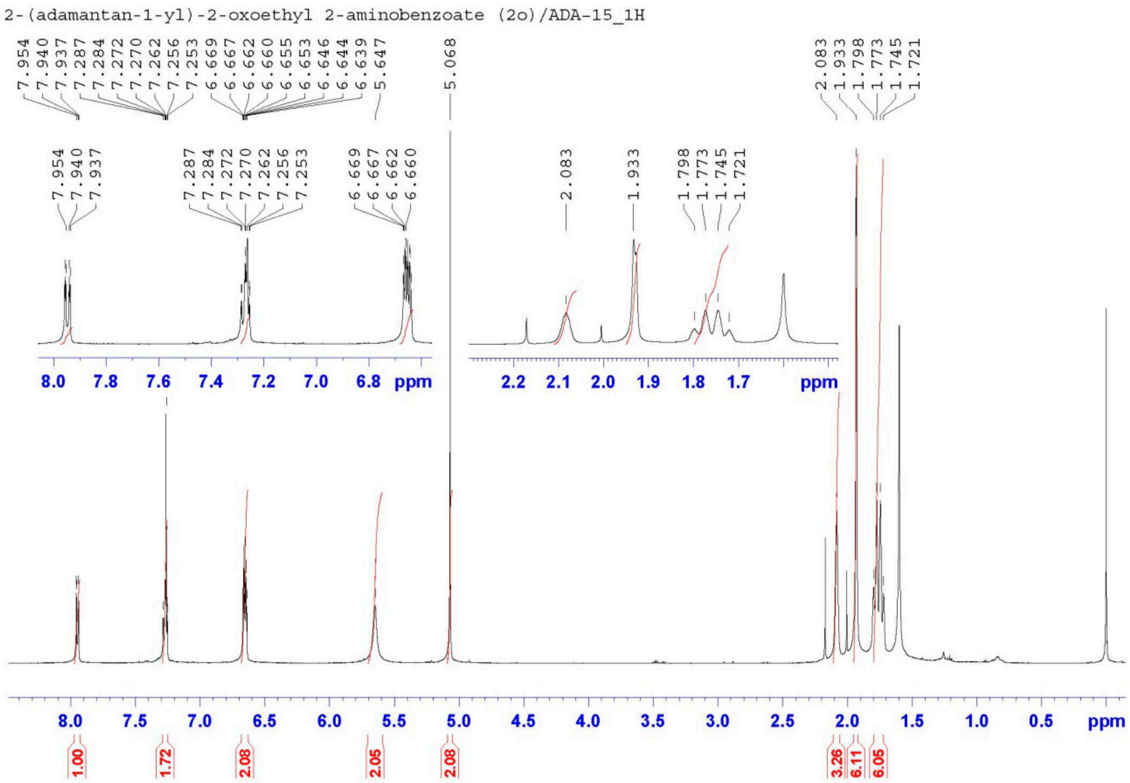

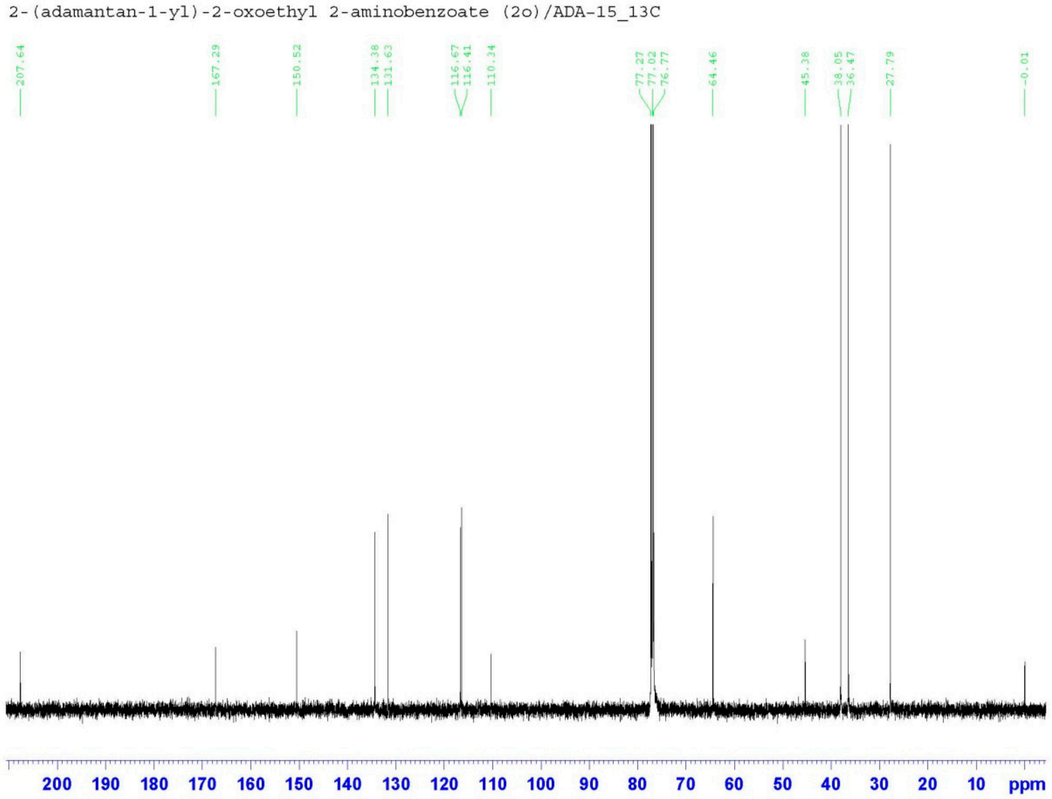

Figure S16. *Cont.*

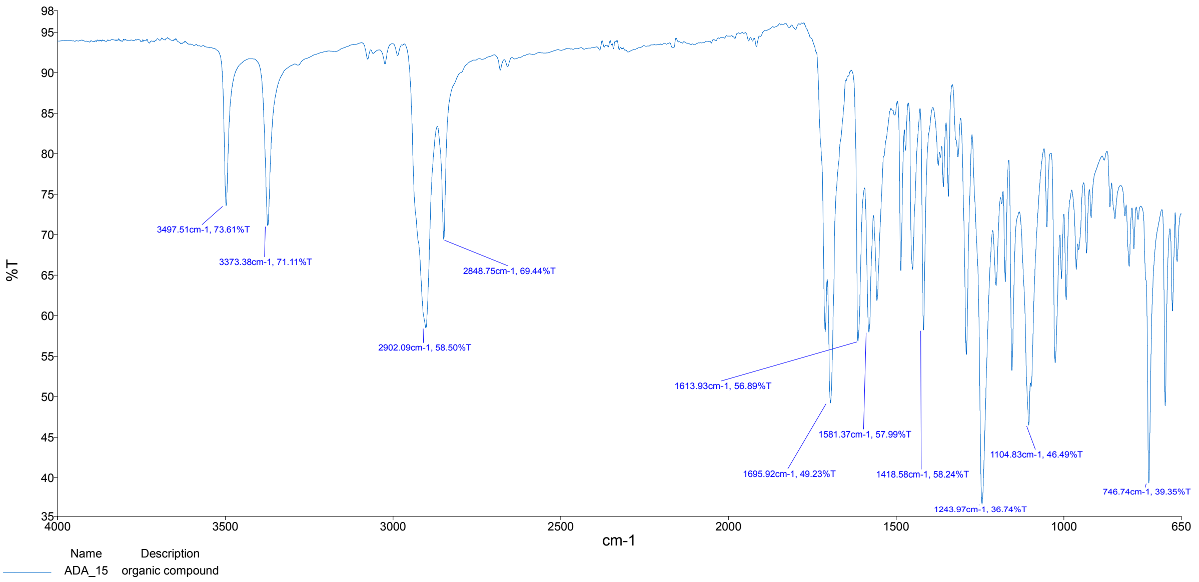

Figure S16. ^{1}H-NMR, ^{13}C-NMR and FTIR spectra of *2-(adamantan-1-yl)-2-oxoethyl 2-aminobenzoate* (**2o**).

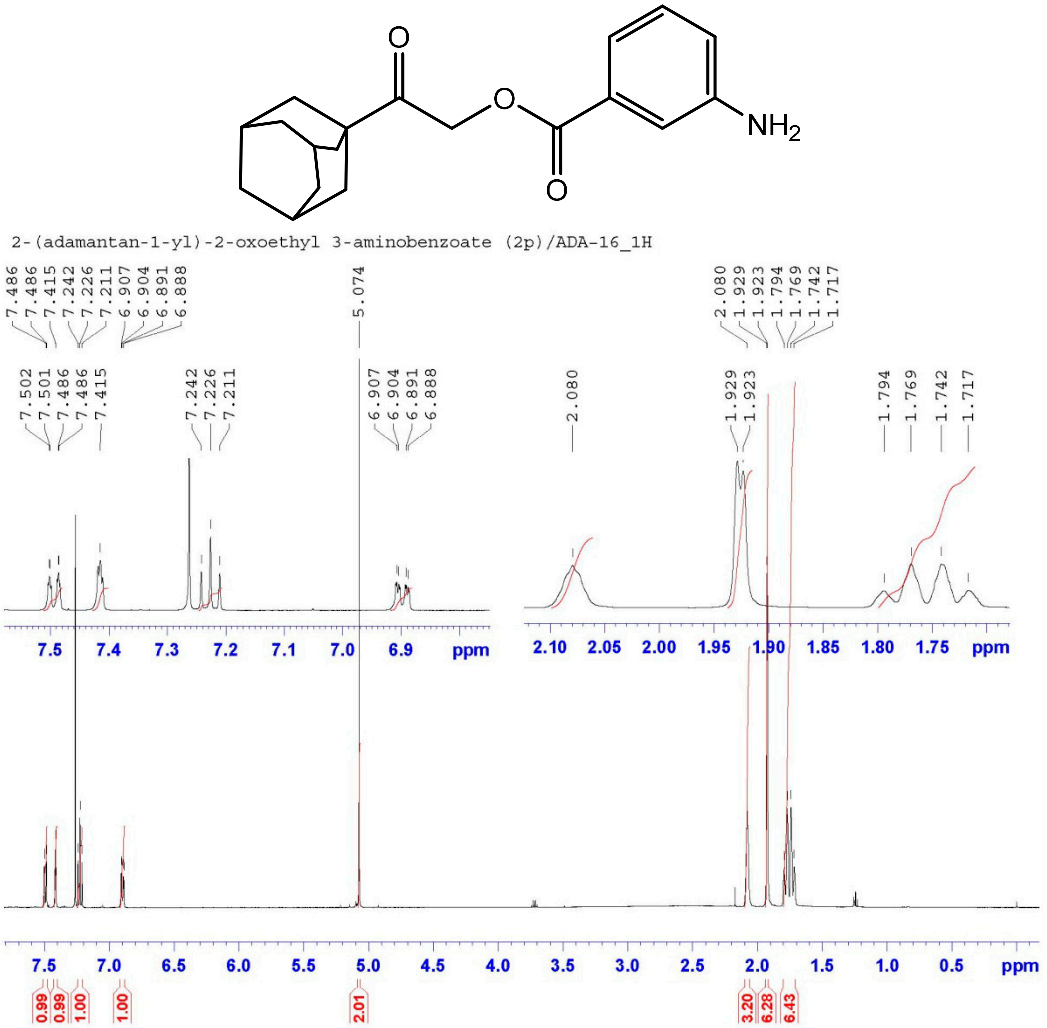

Figure S17. *Cont.*

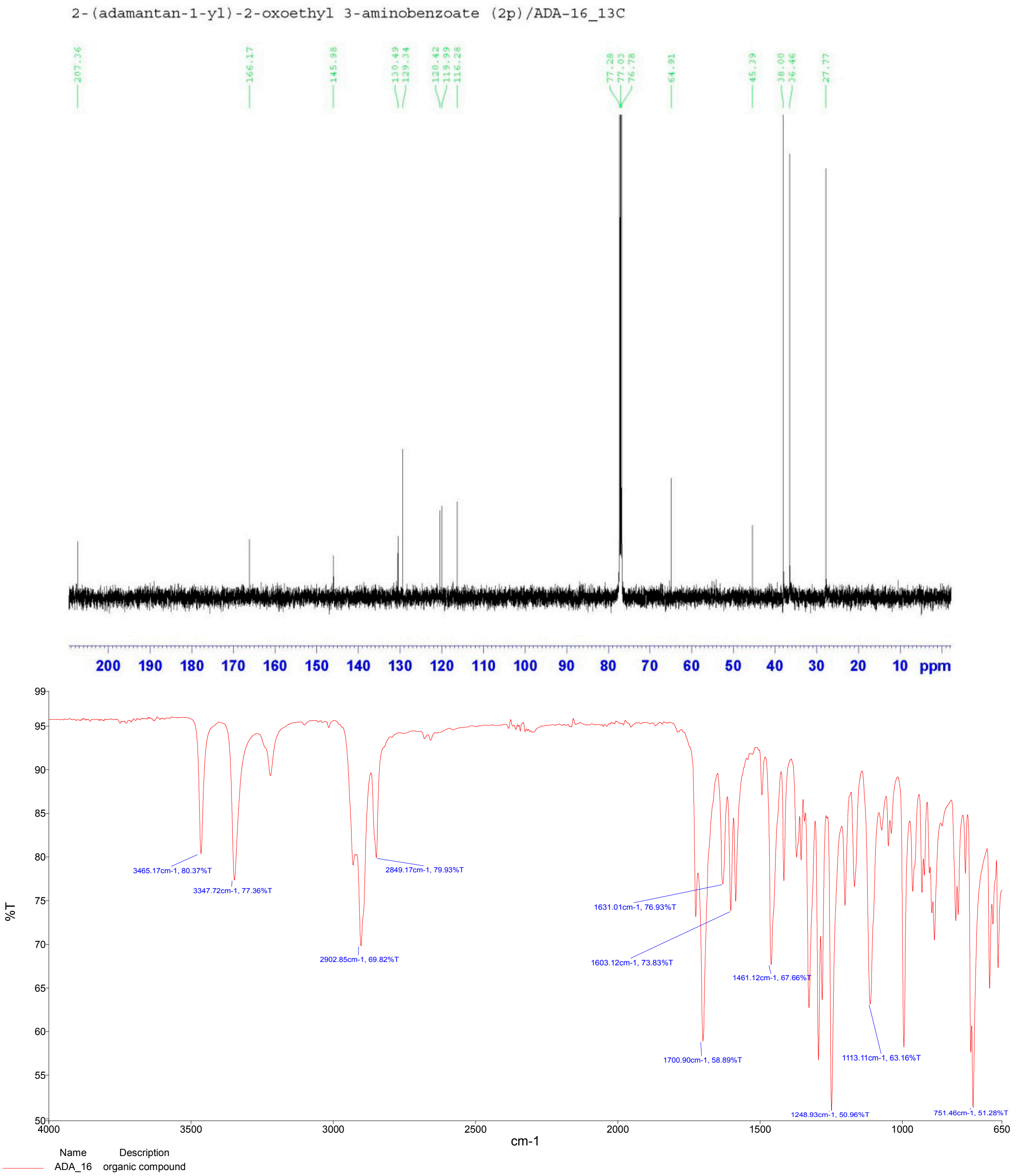

Figure S17. ^{1}H-NMR, ^{13}C-NMR and FTIR spectra of *2-(adamantan-1-yl)-2-oxoethyl 3-aminobenzoate* (**2p**).

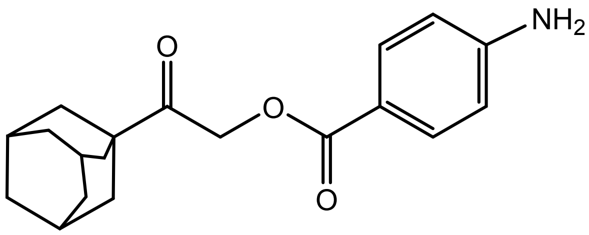

Figure S18. *Cont.*

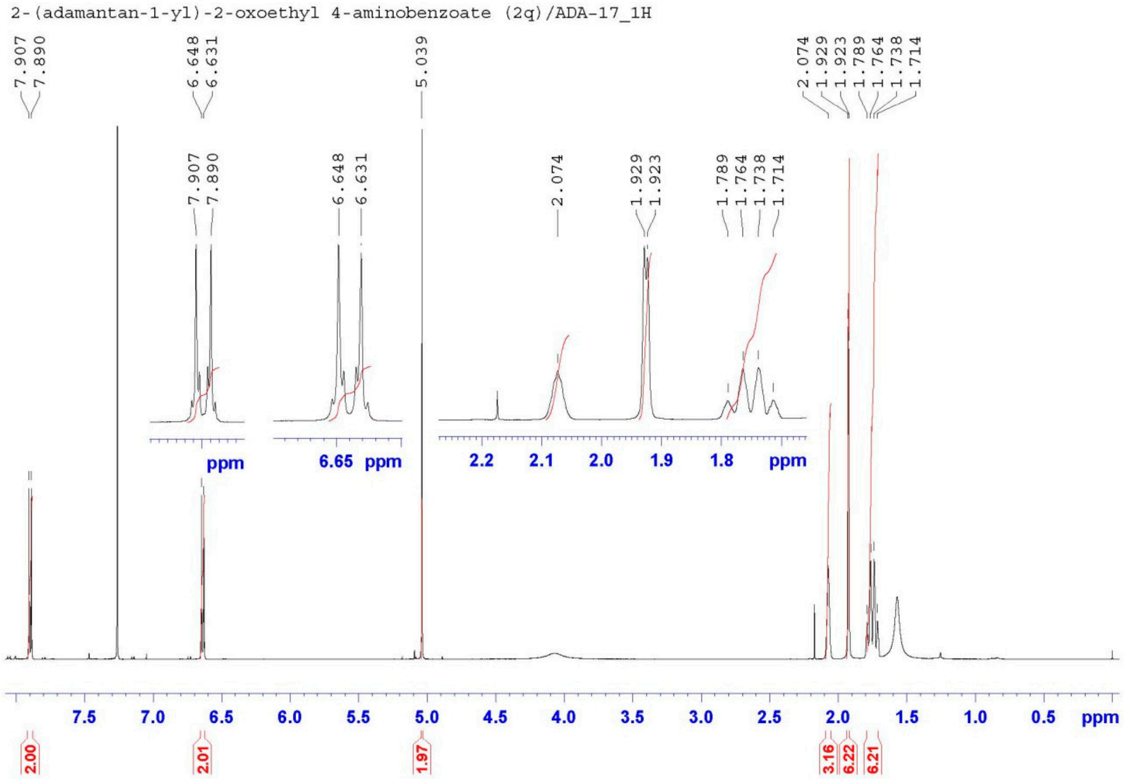

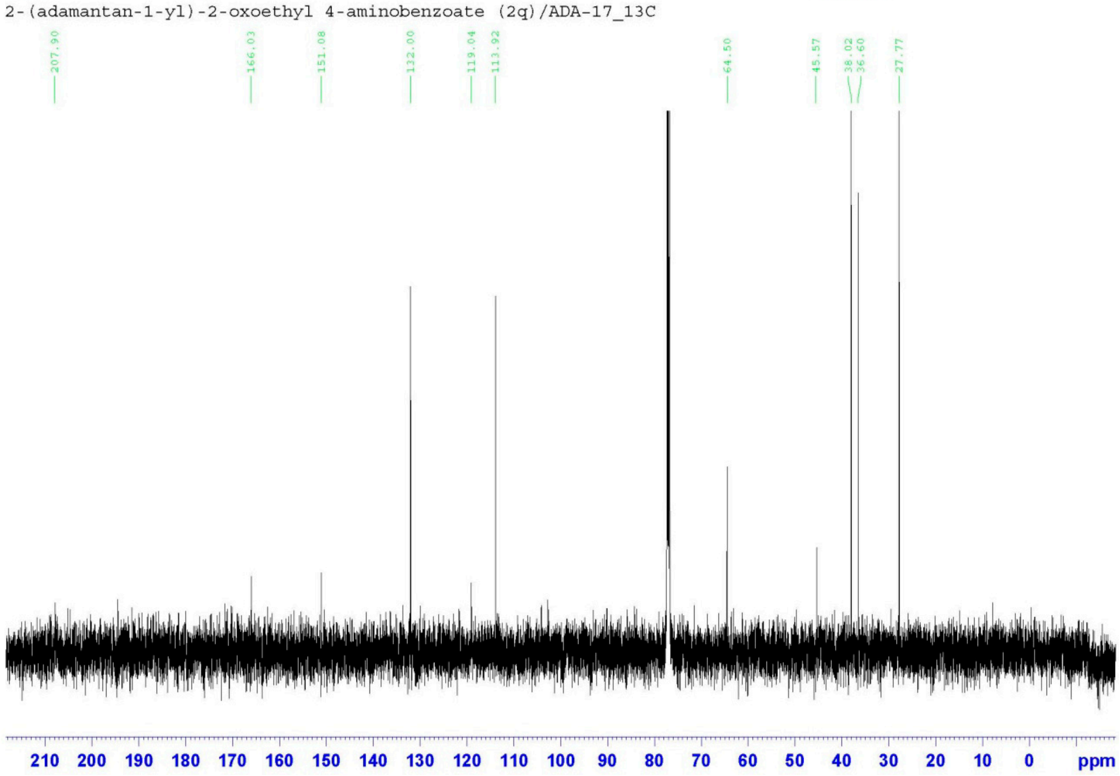

Figure S18. *Cont.*

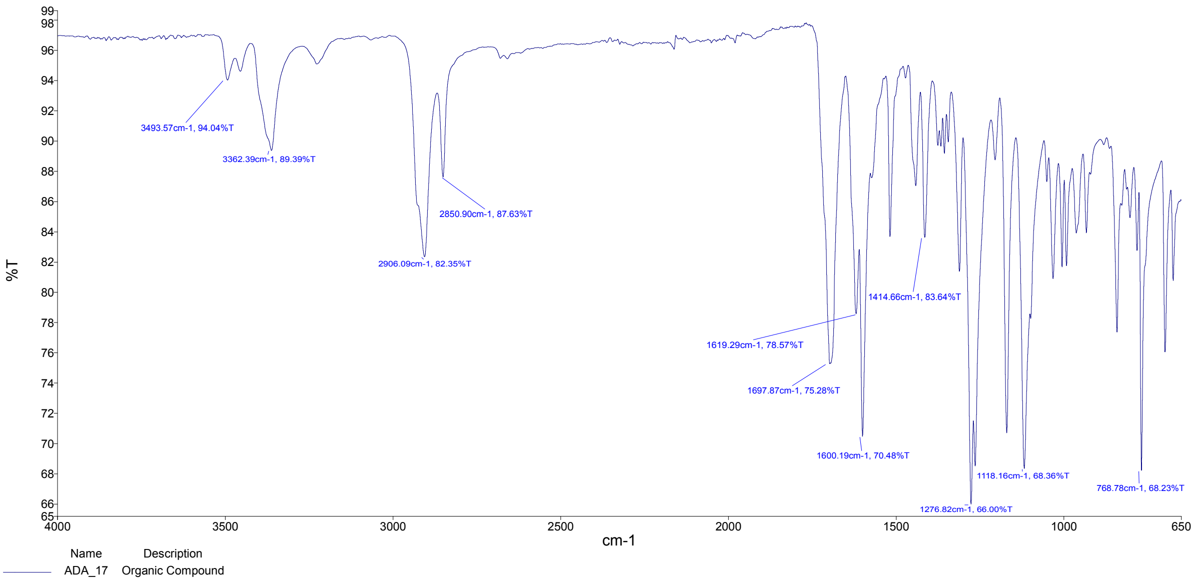

Figure S18. ^{1}H-NMR, ^{13}C-NMR and FTIR spectra of *2-(adamantan-1-yl)-2-oxoethyl 4-aminobenzoate* (**2q**).

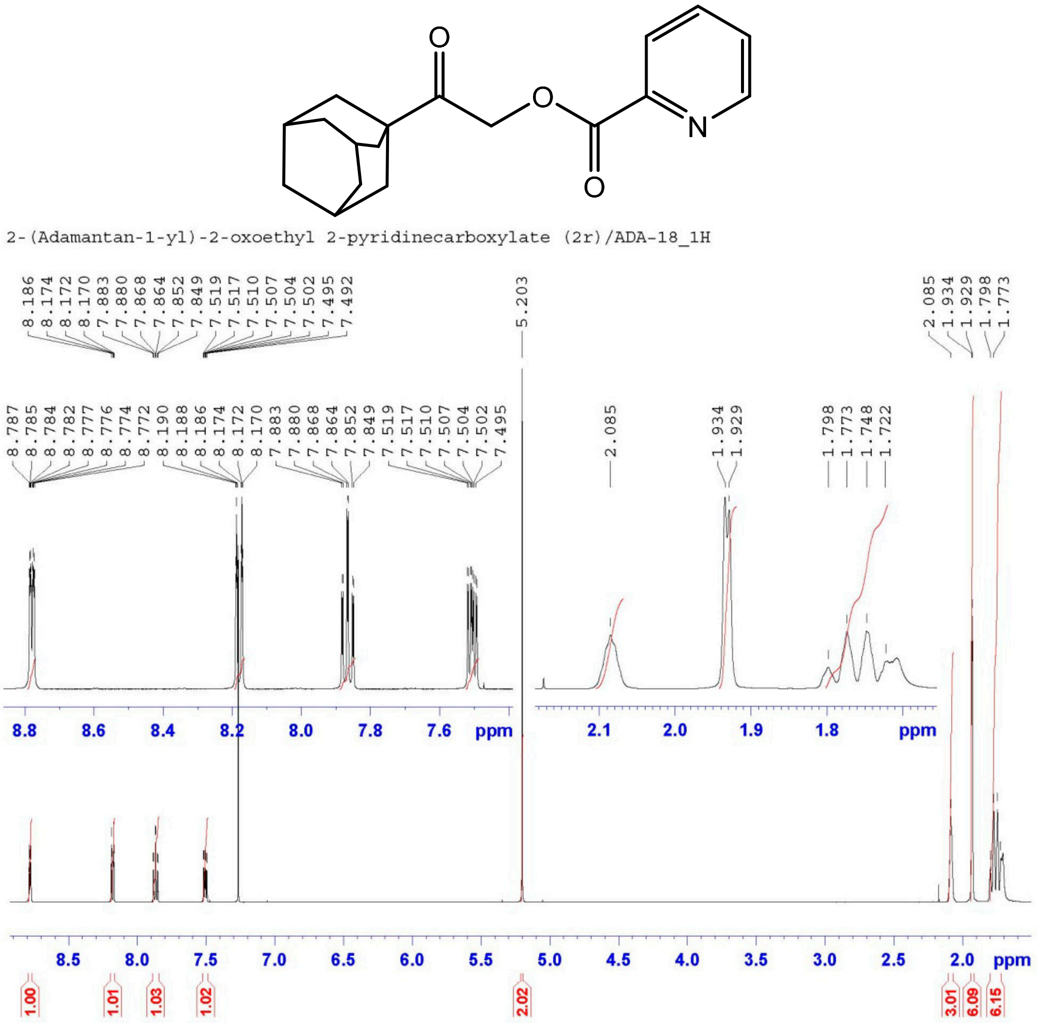

Figure S19. *Cont.*

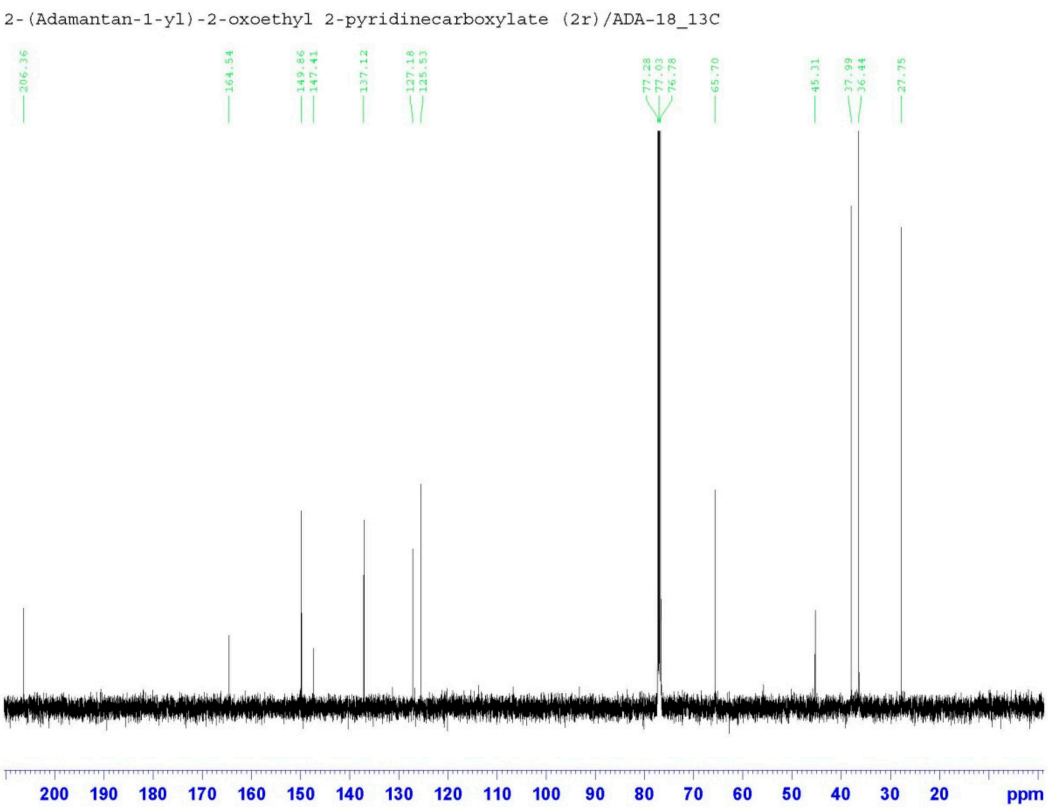

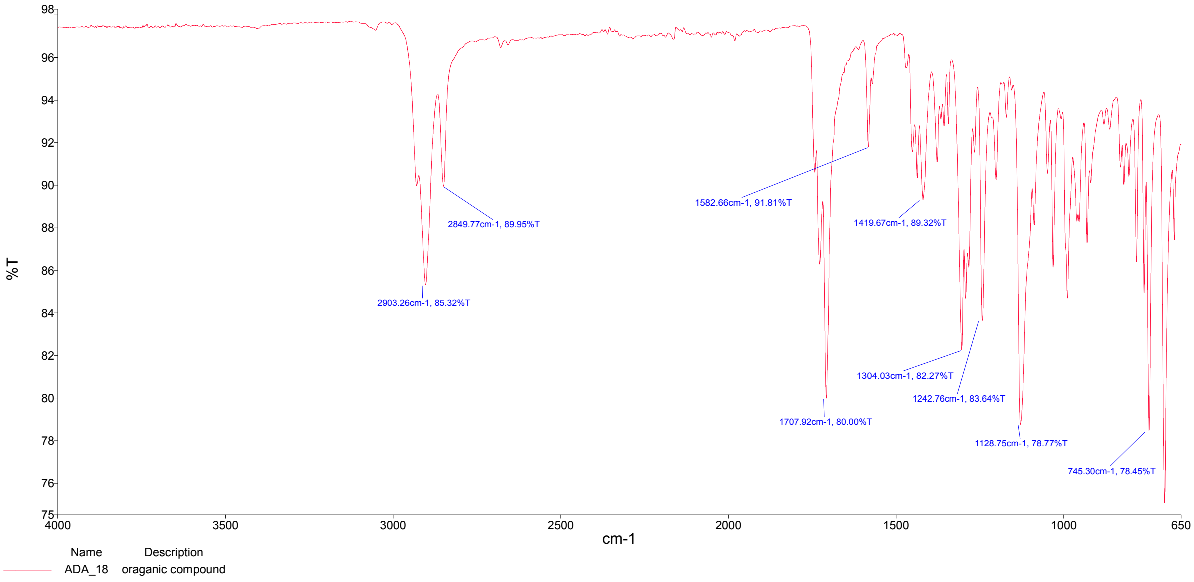

Figure S19. ^{1}H-NMR, ^{13}C-NMR and FTIR spectra of *2-(Adamantan-1-yl)-2-oxoethyl 2-pyridinecarboxylate* (**2r**).

Table S1. Crystal data and parameters for structure refinement for **1**.

**Compound**	**1 (Room Temp.)**	**1 (100 K)**
CCDC deposition number	-	-
Molecular formula	$C_{12}H_{17}BrO$	$C_{12}H_{17}BrO$
Molecular weight	257.16	257.16
Crystal system	Triclinic	Orthorhombic
Space group	$P\bar{1}$	*Pbca*
a (Å)	10.5193 (9)	9.8717 (19)
b (Å)	10.8581 (10)	9.8637 (19)
c (Å)	10.9011 (10)	21.948 (4)
α (°)	107.700 (1)	90
β (°)	90.888 (1)	90
γ (°)	104.295 (1)	90
V (Å^3)	1143.90 (18)	2137.1 (7)
Z	4	8
D_{calc} (g·cm^{-3})	1.242	1.327
Crystal dimensions (mm)	0.48 × 0.36 × 0.19	0.48 × 0.36 × 0.19
μ (mm^{-1})	3.56	3.81
T_{min}/T_{max}	0.102/ 0.168	0.086/ 0.155
Reflections measured	32783	11117
Ranges/indices (*h*, *k*, *l*)	−14, 14; −14, 14; −14, 14	−11, 13; −14, 13; −31, 19
θ limit (°)	2.0–28.7	1.9–30.3
Unique reflections	5882	3163
Observed reflections [$I > 2\sigma(I)$]	3633	2231
Parameters	253	127
Restraints	0	0
Goodness of fit on F^2	1.02	1.02
R_1, wR_2 [$I \geq 2\sigma(I)$]	0.054, 0.182	0.040, 0.099

Table S2. Crystal data and parameters for structure refinement for **2**(**a**–**h**).

Compound	2a	2b	2c	2d	2e	2f	2g	2h
CCDC deposition number	1030854	1030855	1030856	1030857	1030858	1030859	1030860	1030861
Molecular formula	$C_{19}H_{22}O_3$	$C_{19}H_{21}ClO_3$	$C_{19}H_{21}ClO_3$	$C_{19}H_{21}ClO_3$	$C_{19}H_{20}Cl_2O_3$	$C_{20}H_{24}O_3$	$C_{20}H_{24}O_3$	$C_{20}H_{24}O_3$
Molecular weight	298.37	332.81	332.81	332.81	367.25	312.39	312.39	312.39
Crystal system	Triclinic	Monoclinic	Monoclinic	Monoclinic	Monoclinic	Triclinic	Monoclinic	Monoclinic
Space group	$P\bar{1}$	$P2_1/c$	$P2_1/c$	$P2_1/c$	$P2_1/c$	$P\bar{1}$	$P2_1/n$	$P2_1/n$
a (Å)	9.7090 (9)	9.9165 (14)	15.0265 (11)	12.7776 (18)	13.5955 (10)	9.7213 (8)	14.881 (3)	8.9059 (9)
b (Å)	10.1224 (9)	26.0610 (4)	6.4316 (5)	6.4887 (9)	10.0877 (8)	10.0095 (8)	6.4393 (12)	6.4556 (7)
c (Å)	17.7587 (16)	13.6754 (19)	19.4135 (11)	19.8490 (3)	26.5950 (3)	18.9536 (13)	18.255 (3)	29.368 (3)
α (°)	77.035 (2)	90	90	90	90	75.912 (2)	90	90
β (°)	77.876 (2)	109.437 (2)	118.792 (4)	90.267 (2)	104.881 (2)	84.644 (2)	108.018 (3)	97.593 (2)
γ (°)	71.659 (2)	90	90	90	90	73.022 (1)	90	90
V (Å^3)	1596.0 (3)	3332.8 (8)	1644.3 (2)	1645.7 (4)	3525.1 (6)	1710.4 (2)	1663.4 (5)	1673.6 (3)
Z	4	8	4	4	8	4	4	4
D_{calc} (g·cm^{-3})	1.242	1.327	1.344	1.343	1.384	1.213	1.247	1.24
Crystal dimensions (mm)	1.42 × 0.33 × 0.32	0.51 × 0.15 × 0.11	0.60 × 0.51 × 0.21	0.48 × 0.33 × 0.11	0.66 × 0.12 × 0.06	0.57 × 0.42 × 0.14	0.32 × 0.18 × 0.08	0.64 × 0.35 × 0.29
μ (mm^{-1})	0.08	0.24	0.25	0.25	0.38	0.08	0.08	0.08
T_{min}/T_{max}	0.892/0.974	0.888/0.974	0.866/0.950	0.891/0.975	0.788/0.978	0.956/0.989	0.974/0.993	0.958/0.988
Reflections measured	26726	45418	20743	14606	51013	27531	14965	12089
Ranges/indices (h, k, l)	−12, 12; −13, 13; −22, 23	−13, 13; −36, 36; −19, 19	−22, 22; −9, 8; −28, 28	−16, 16; −8, 8; −25, 25	−15, 16; −11, 11; −31, 31	−11, 11; −12, 12; −23, 23	−19, 19; −8, 8: −23, 23	−10, 10; −7, 7; −35, 36
θ limit (°)	2.4–27.4	2.4–20.2	2.4–29.1	3.2–23.3	2.5–19.0	2.2–27.8	2.9–22.0	2.8–23.6
Unique reflections	7315	9729	5669	3793	6190	6684	3801	3232
Observed reflections [$I > 2\sigma(I)$]	5306	4193	3551	2541	3574	4845	2057	2301
Parameters	561	497	208	208	433	581	209	209
Restraints	0	0	0	0	0	72	0	0
Goodness of fit on F^2	1.03	1.01	1.03	1.04	1.04	1.03	1.02	1.07
R_1, wR_2 [$I \geq 2\sigma(I)$]	0.065, 0.242	0.063, 0.216	0.050, 0.168	0.046, 0.148	0.081, 0.280	0.065, 0.218	0.045, 0.154	0.044, 0.131

Table S3. Crystal data and parameters for structure refinement for **2**(**i–l**, **n**, **o**, **p** and **r**).

Compound	2i	2j	2k	2l	2n	2o	2p	2r
CCDC deposition number	1030862	1030863	1030864	1030865	1030866	1030867	1030868	1030869
Molecular formula	$C_{20}H_{24}O_4$	$C_{20}H_{24}O_4$	$C_{20}H_{24}O_4$	$C_{19}H_{21}NO_5$	$C_{19}H_{21}NO_5$	$C_{19}H_{21}NO_3$	$C_{19}H_{23}NO_3$	$C_{18}H_{21}NO_3$
Molecular weight	328.39	328.39	328.39	343.37	343.37	311.37	313.38	299.36
Crystal system	Triclinic	Monoclinic	Monoclinic	Triclinic	Monoclinic	Monoclinic	Orthorhombic	Monoclinic
Space group	$P\bar{1}$	$P2_1/n$	$P2_1/n$	$P\bar{1}$	$P2_1/c$	$P2_1/c$	$Pca2_1$	$P2_1/n$
a (Å)	7.9923 (4)	6.4434 (7)	9.4908 (9)	7.3004 (5)	12.8819 (9)	6.4577 (9)	10.5404 (14)	12.6664 (17)
b (Å)	10.6701 (6)	17.725 (2)	6.4798 (6)	10.4009 (7)	6.4845 (5)	26.664 (4)	18.371 (3)	6.6858 (9)
c (Å)	20.1249 (11)	14.9809 (17)	28.343 (3)	24.1685 (16)	20.0028 (13)	9.9048 (14)	8.3774 (11)	19.333 (3)
α (°)	90.813 (1)	90	90	90.137 (1)	90	90	90	90
β (°)	92.969 (1)	97.771 (2)	99.060 (2)	94.274 (1)	90.628 (1)	107.399 (3)	90	105.455 (2)
γ (°)	90.557 (1)	90	90	110.405 (1)	90	90	90	90
V (Å^3)	1713.65 (16)	1695.3 (3)	1721.3 (3)	1714.4 (2)	1670.8 (2)	1627.5 (4)	1622.1 (4)	1578.0 (4)
Z	4	4	4	4	4	4	4	4
D_{calc} (g·cm^{-3})	1.273	1.287	1.267	1.33	1.365	1.271	1.283	1.26
Crystal dimensions (mm)	0.55 × 0.44 × 0.36	0.87 × 0.59 × 0.40	0.54 × 0.42 × 0.37	0.57 × 0.41 × 0.22	0.71 × 0.51 × 0.34	0.26 × 0.20 × 0.08	0.55 × 0.27 × 0.07	0.56 × 0.35 × 0.10
μ (mm^{-1})	0.09	0.09	0.09	0.10	0.10	0.09	0.09	0.09
T_{min}/T_{max}	0.954/0.969	0.927/0.965	0.955/0.969	0.947/0.980	0.933/0.967	0.978/0.993	0.954/0.994	0.954/0.991
Reflections measured	47595	11481	10969	24440	22905	11958	11421	11552
Ranges/indices (h, k, l)	−12, 12; −16, 16; −30, 30	−7, 7; −14, 21; −18, 18	−11, 11; −7, 5; −34, 31	−9, 8; −12, 12; −29, 29	−15, 15; −8, 8; −24, 24	−7, 7; −31, 31; −11, 11	−13, 12; −22, 22; −10, 10	−15, 15; −6, 8; −23, 23
θ limit (°)	2.7–31.0	2.7–25.5	2.2–27.6	3.0–25.8	2.6–26.0	2.6–27.4	2.2–22.1	3.3–21.8
Unique reflections	12842	3309	3359	6664	3317	2856	3191	3116
Observed reflections [$I > 2\sigma(I)$]	7598	2555	2499	5100	2786	1720	2602	1597
Parameters	435	300	238	489	226	245	216	399
Restraints	0	0	0	0	0	24	1	65
Goodness of fit on F^2	1.03	1.06	1.04	1.03	1.03	1.03	1.02	1.03
R_1, wR_2 [$I \geq 2\sigma(I)$]	0.060, 0.212	0.042, 0.114	0.054, 0.177	0.051, 0.172	0.042, 0.126	0.061, 0.200	0.041, 0.105	0.051, 0.188

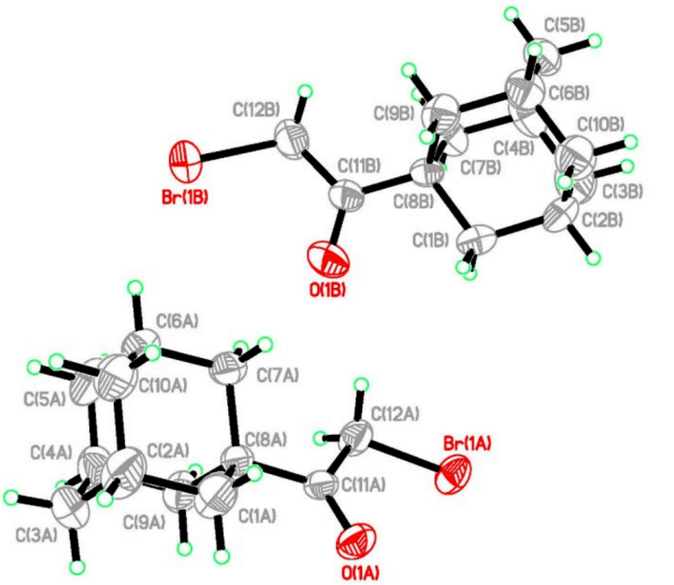

1 (polymorph *a*)

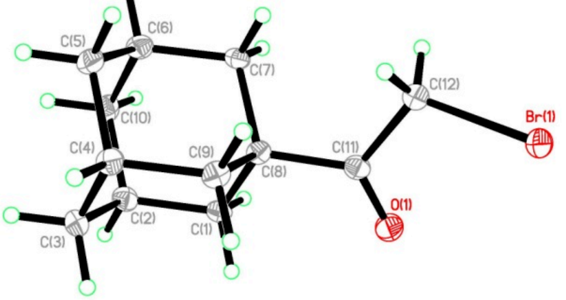

1 (polymorph *b*)

Figure S20. The molecular structures of **1** (polymorph *a* and *b*) with atom numbering schemes and 50% probability displacement ellipsoids.

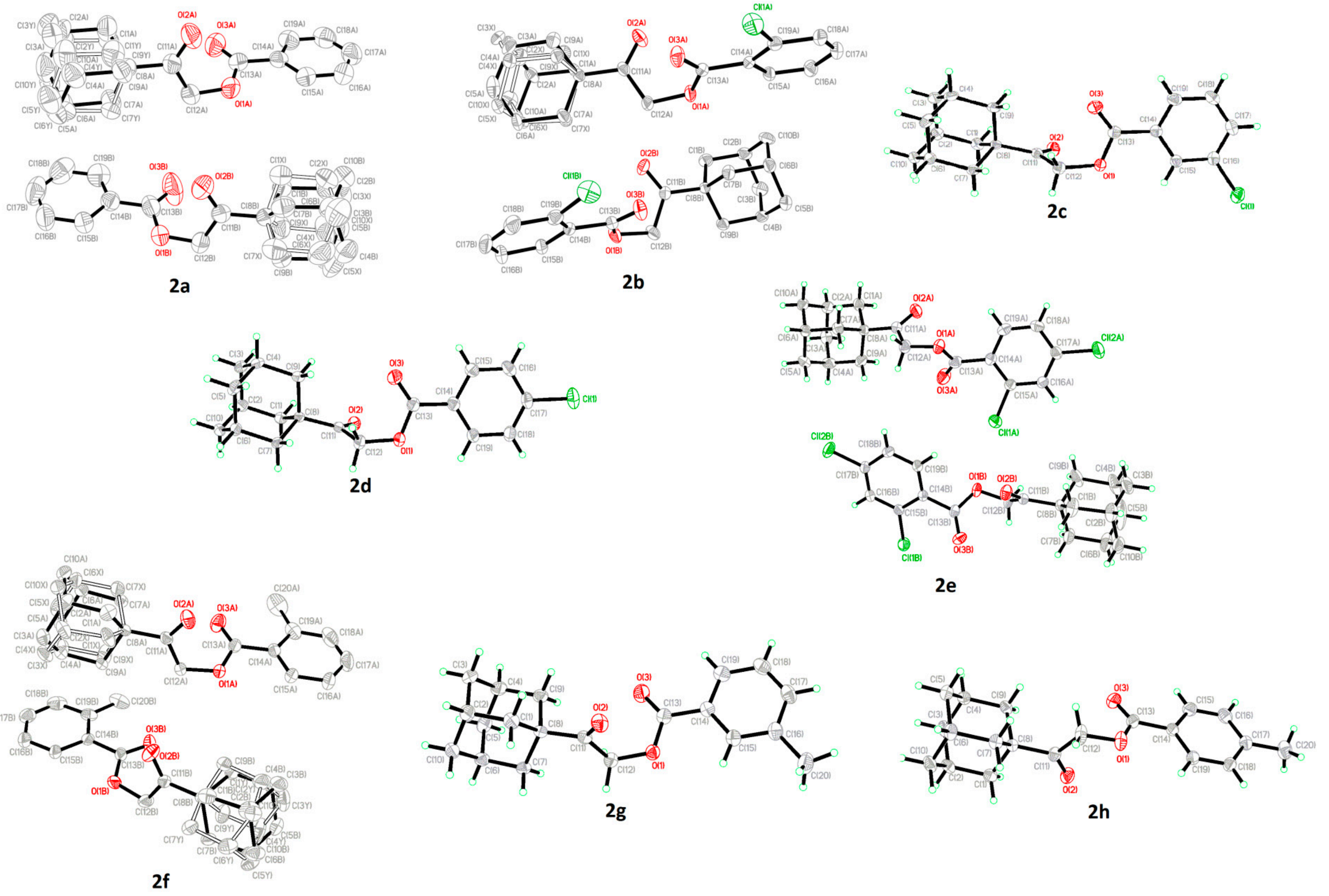

Figure S21. The molecular structures of **2**(**a**–**h**) with atom numbering schemes and 20% probability displacement ellipsoids. Hydrogen atoms for disordered structure (**2a**, **2b** and **2f**) were omitted for clarity.

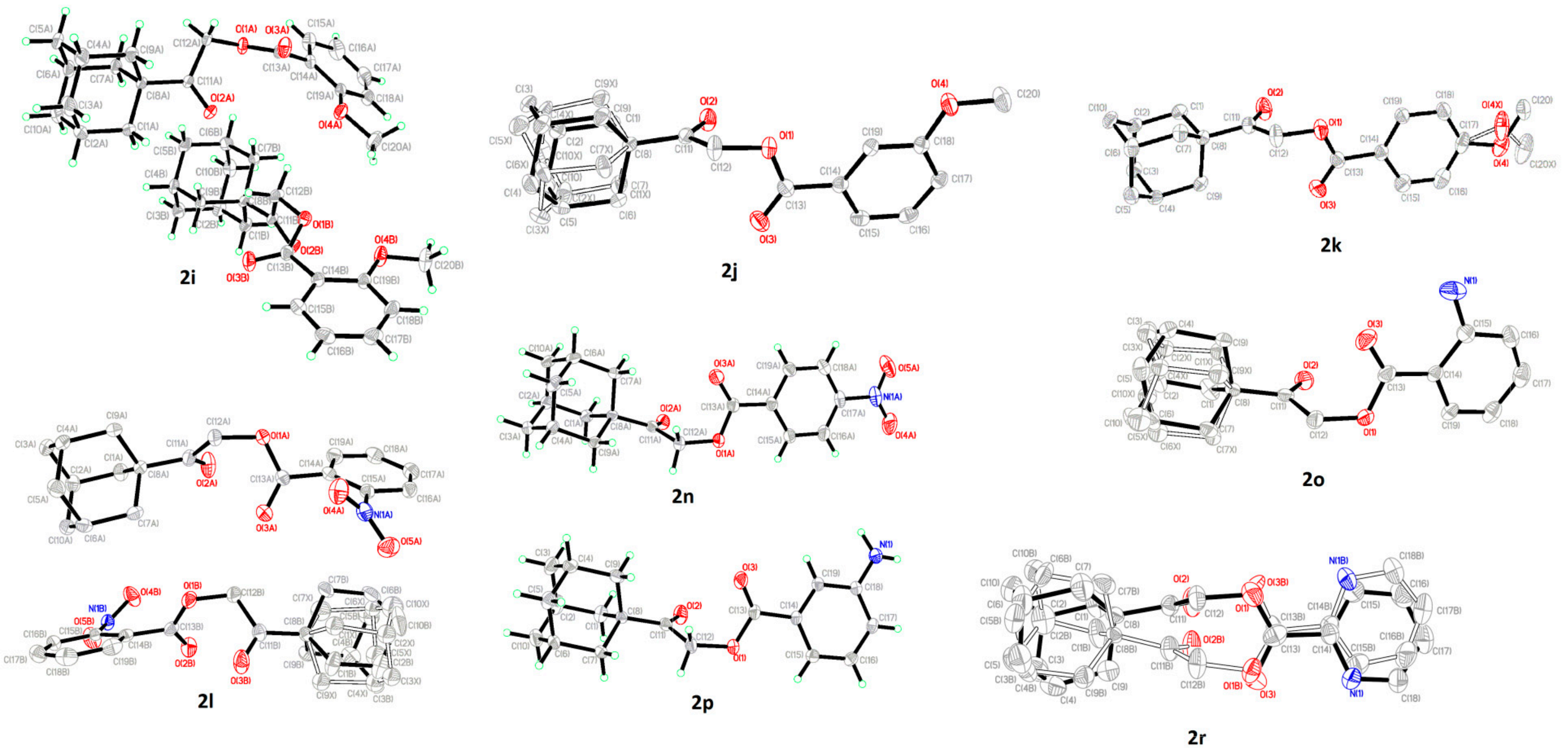

Figure S22. The molecular structures of **2**(**i–l**, **n**, **o**, **p** and **r**) with atom numbering schemes and 20% probability displacement ellipsoids. Hydrogen atoms for disordered structure **2(j–l**, **o** and **r**) were omitted for clarity.

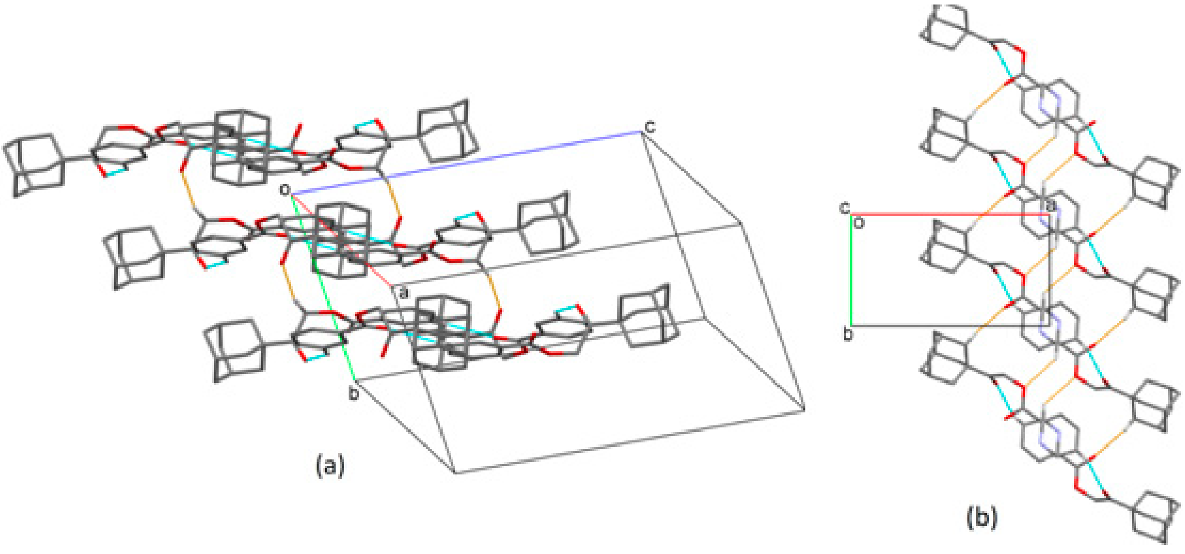

Figure S23. (**a**) Partial packing diagram of **2a** shows hydrogen-bonded column along *a*-axis (blue dashed lines indicated the tetramer hydrogen bonds whereas orange dashed lines indicated the hydrogen bonds that link the sets of tetramer into [100] column); (**b**) Partial packing diagram of **2r** view along *c*-axis. In the crystal packing of **2a**, molecules *A* and *B* are linked into tetramers via weak intermolecular C18A—H18A···O3B and C18B—H18B···O2A hydrogen bonds; these tetramers were further connected into columns along [100] direction via another weak intermolecular C12A—H12A···O2B hydrogen bond (**2a** in Figure 2). In the crystal of **2r**, molecules are linked into centrosymmetric dimers via a weak C17—H17A···O2 hydrogen bond. Similar to those one-dimensional hydrogen-bonded pattern found in compound **2a**, the dimers are also linked into columns along [010] direction via weak intermolecular C7—H7A···O3 and C18—H18A···O1 hydrogen bonds.

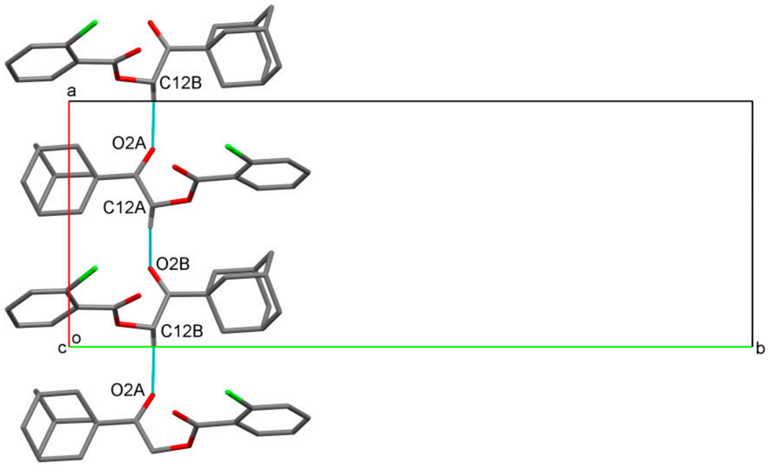

Figure S24. Partial packing diagram of **2b** viewed along the c-axis showing a single [100] chain. In the crystal of **2b**, molecules are connected in an alternate pattern, ...*ABAB*... pattern into infinite linear chain, running along the *a*-axis via weak intermolecular C12A—H12B···O2B and C12B—H12C···O2A hydrogen bonds.

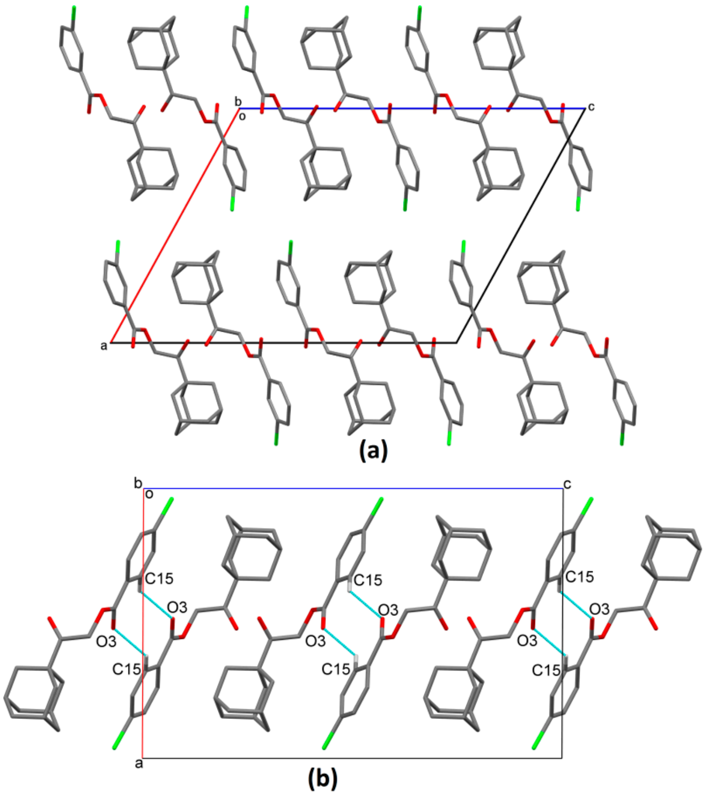

Figure S25. Packing diagrams of (**a**) **2c**; (**b**) **2d** viewed along *b*-axis. Hydrogen atoms that are not involved in hydrogen bonds were omitted for clarity. Compounds **2c** and **2d** exhibit crystal packing with 2D structural similarity which parallel to *bc*-plane.

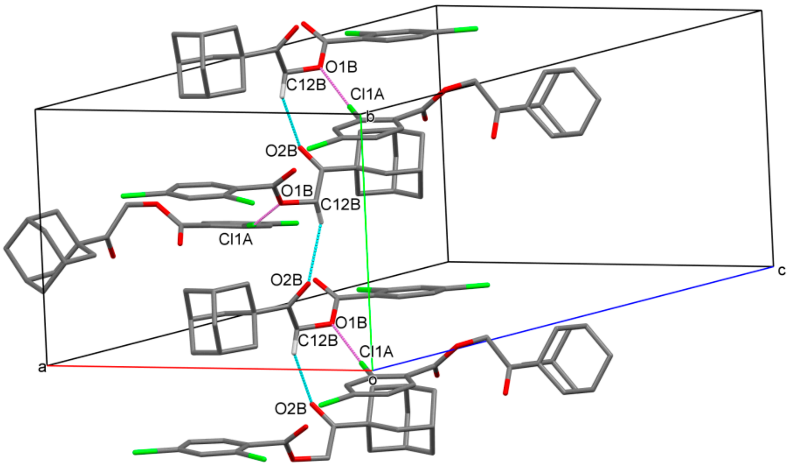

Figure S26. Partial packing diagram of **2e** showing a [010] chain (blue dashed lines indicated hydrogen bonds and short contacts are represented by violet dashed lines). There is no significant hydrogen bond observed in the crystal of **2c**. In the crystal of **2d**, the molecules are linked into centrosymmetric dimers via weak intermolecular C15—H15A···O3 hydrogen bond. In the crystal of **2e**, molecules *B* are linked into 1D chains via weak intermolecular C12B—H12C···O2B hydrogen bonds, propagating along *b*-axis, together with short intermolecular Cl1A···O1B contacts of 3.028(10) and 3.270(10) Å in between molecules *A* and *B*.

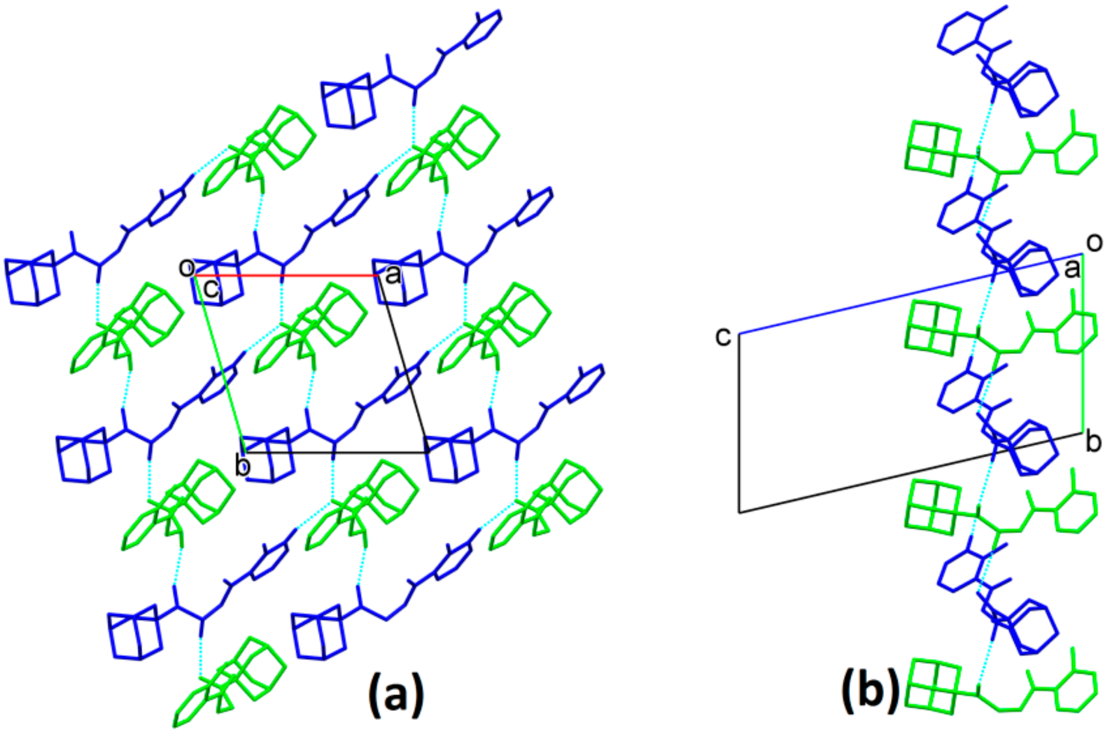

Figure S27. Partial packing diagram of **2f** with a ...*ABAB*... pattern, viewed along the (**a**) *c*-; and (**b**) *a*-axes. In the crystal of **2f**, molecules are linked into 1D chain in a ...*ABAB*... pattern along [010] via weak C12A—H12A···O2B and C12B—H12D···O2A hydrogen bonds. Atom O2A acts as a bifurcated hydrogen bond acceptor and link 1D chains into 2D (001) plane.

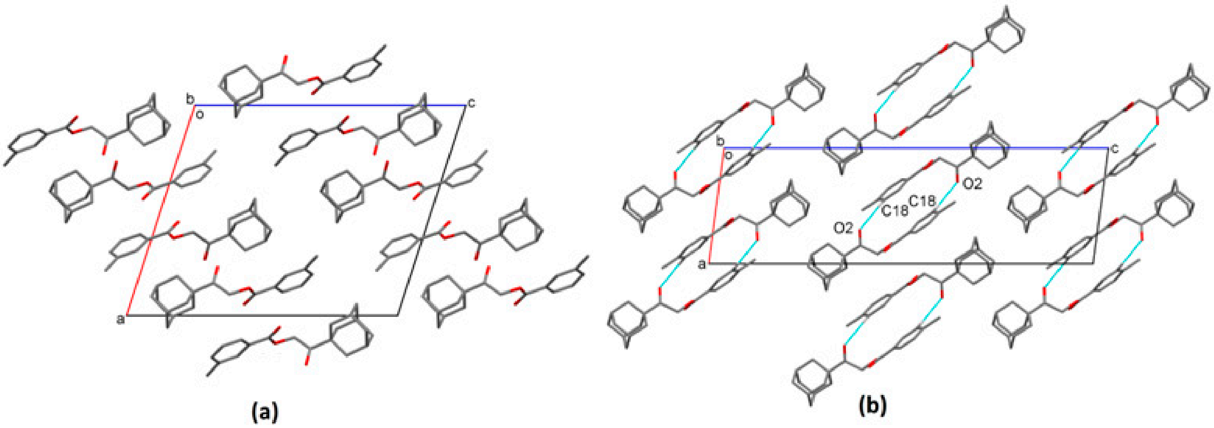

Figure S28. Packing diagrams of (**a**) **2g**; (**b**) **2h** viewed along *b*-axis. Hydrogen atoms that are not involved in hydrogen bonds were omitted for clarity. No significant hydrogen bonding has been observed in **2g**, whereas molecules of **2h** are linked into dimers by weak C18—H18A···O2 hydrogen bond.

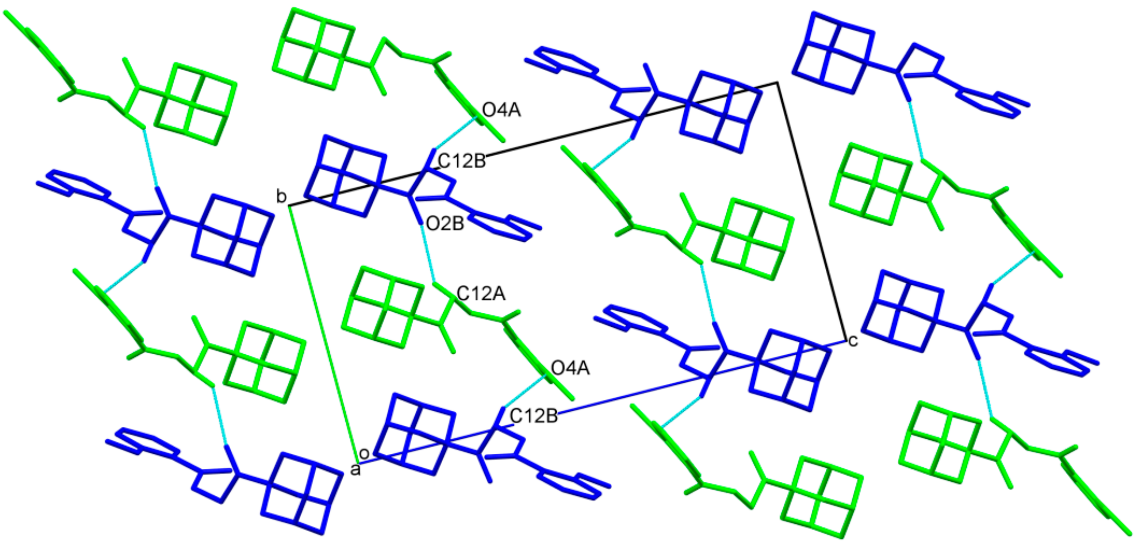

Figure S29. Packing diagrams of **2i** viewed along *a*-axis. Hydrogen atoms that are not involved in hydrogen bonds were omitted for clarity. In the crystal of **2i**, molecules *A* and *B* are linked into 1D chain along *b*-axis via weak intermolecular C12A—H12A···O2B and C12B—H12C···O4A hydrogen bonds.

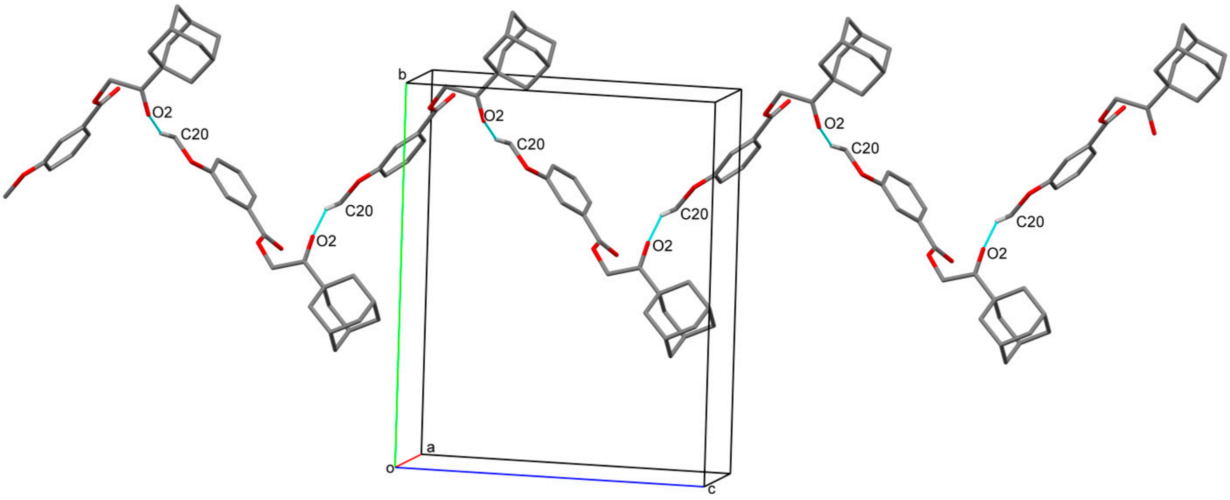

Figure S30. Partial packing diagram of **2j** forming a zigzag chain. Hydrogen atoms that are not involved in hydrogen bonds were omitted for clarity. In the crystal of **2j**, molecules are connected into infinite zigzag chains, running along [101] direction via weak intermolecular C20—H20A···O2 hydrogen bond.

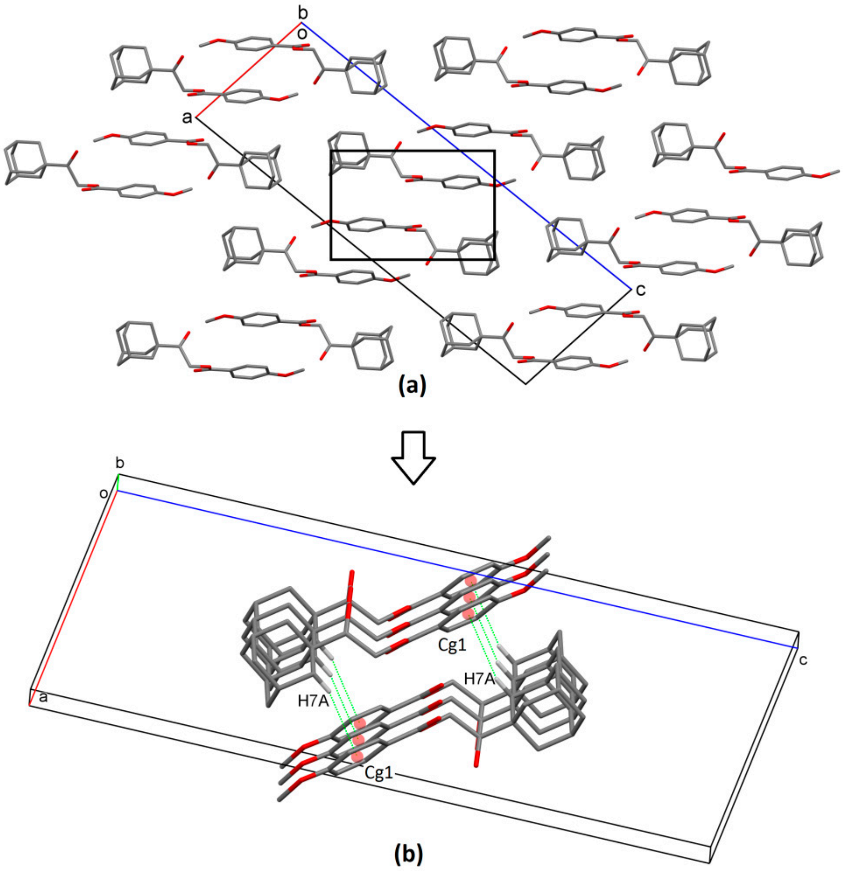

Figure S31. Packing diagrams of (**a**) **2k** and (**b**) C—H···π interaction viewed along *b*-axis. Hydrogen atoms which are not involved in hydrogen bonds were omitted for clarity. There is no significant hydrogen bond found in **2k**, however, C—H···π (C14-C19) interaction connects molecules into dimer-like motifs stacked along *b*-axis which is similar to **2h**.

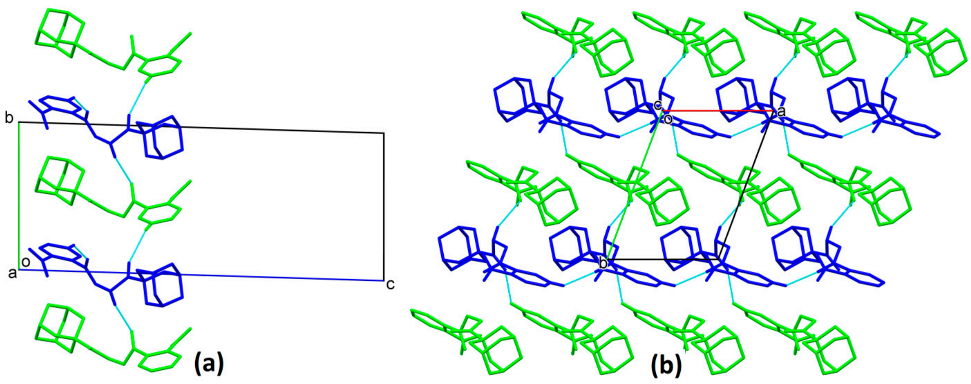

Figure S32. Partial packing diagram of **2l** viewed along (**a**) *a*- and (**b**) *c*-axes. Hydrogen atoms that are not involved in hydrogen bonds were omitted for clarity. In the crystal of **2l**, molecule *A* and *B* are linked into 1D chain with ...*ABAB*... pattern via weak intermolecular C18A—H18A···O2B and C12B—H12C···O2A hydrogen bonds (**a**). This 1D chain is linked into a 2D plane parallel to *ab*-plane through weak intermolecular C18A—H18A···O2B hydrogen bond (**b**).

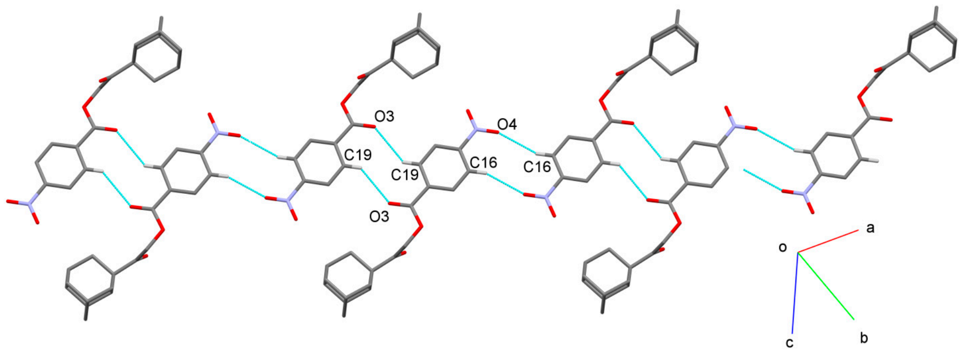

Figure S33. A 1D chain formed in the crystal packing of **2n**. Hydrogen atoms that are not involved in hydrogen bonds were omitted for clarity. In the crystal of **2n**, weak C16—H16···O4 and C19—H19···O3 hydrogen bonds link the molecules into a [110] chain comprised of R_2^2 (10) dimers.

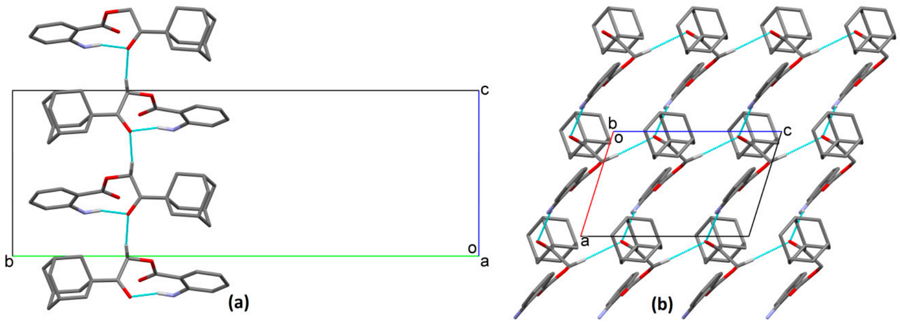

Figure S34. Partial packing diagram of **2o** viewed along the (**a**) *a*-; and (**b**) *b*-axes. Hydrogen atoms that are not involved in hydrogen bonds were omitted for clarity. In the crystal of **2o**, atom O2 acts as bifurcated acceptor and it was linked to two adjacent molecules via weak C12—H12B···O2 and N1—H1N1···O2 hydrogen bonds into 2D layers parallel to (010) plane.

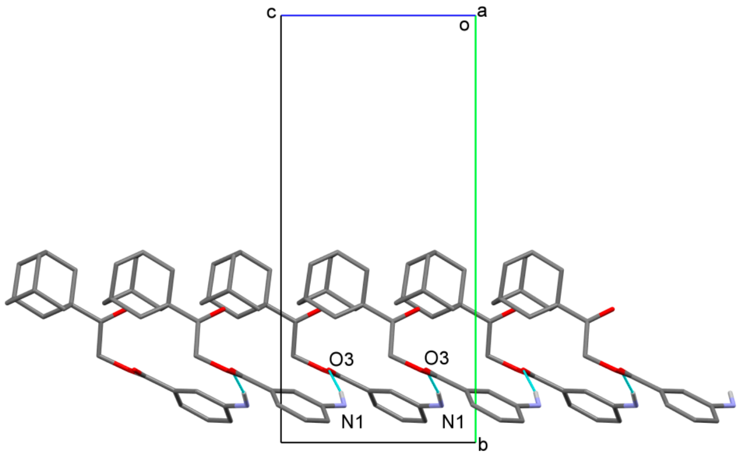

Figure S35. Partial packing diagram of **2p** which forms infinites linear chain along *c*-axis. Hydrogen atoms that not involved in hydrogen bonds were omitted for clarity. In the crystal of **2p**, molecules are linked into 1D chains via strong intermolecular N1—H1N1···O3 hydrogen bond, propagating along the *c*-axis.

Table S4. Hydrogen bond geometries for **2**(**a**–**l**, **n**, **o**, **p** and **r**).

D—H···*A*	D—H (Å)	H···*A* (Å)	D···*A* (Å)	D—H···*A* angles (°)	Symmetry Code
**2a**					
C12A—H12*A*···O2B	0.97	2.45	3.3772	159	*x* − 1, *y* + 1, *z*
C18A—H18*A*···O3B	0.93	2.46	3.2550	143	−*x* + 1, −*y* + 1, −*z*
C18B—H18*B*···O2A	0.93	2.55	3.3442	144	*x* + 2, *y* − 1, *z*
**2b**					
C12*A*—H12*B*···O2B	0.97	2.55	3.4902	162	*x*, −*y* − 1/2, *z* − 1/2
C12*B*—H12*C*···O2*A*	0.97	2.54	3.5038	175	*x* − 1, −*y* − 1/2, *z* − 3/2
**2c**					
no H-bond observed					
**2d**					
C15—H15*A*···O3	0.93	2.59	3.3680	141	−*x* + 1, −*y* + 1, −*z*
**2e**					
C12*B*—H12*C*···O2*B*	0.97	2.58	3.2241	124	−*x* + 1,*y* − 1/2, −*z* + 1/2
**2f**					
C12A—H12A···O2B	0.97	2.54	3.4937	167	
C12B—H12D···O2A	0.97	2.55	3.5155	174	*x*,1 + *y*,*z*
C18B—H18B···O2A	0.93	2.55	3.4581	166	1 + *x*,*y*,*z*
**2g**					
no H-bond observed					
**2h**					
C18—H18*A*···O2	0.93	2.59	3.4859	162	−*x* + 2, −*y* + 1, −*z*
**2i**					
C12A—H12*A*···O2B	0.97	2.57	3.2557	128	
C12B—H12*C*···O4A	0.97	2.50	3.3997	154	*x*,1 + *y*,*z*
**2j**					
C20—H20*A*···O2	0.96	2.59	3.4576	151	*x* − 3/2, −*y* − 3/2, *z* − 3/2
**2k**					
C7—H7*A* ···*Cg*1[1]	0.97	2.92	3.8111	154	
**2l**					
C12*B*—H12*C*···O2*A*	0.97	2.53	3.4511	158	*x* + 1, *y*, *z*
C18*A*—H18*A*···O2*B*	0.93	2.48	3.1938	134	*x* − 2, *y* − 1, *z*
C18*B*—H18*B*···O3*B*	0.93	2.43	3.2453	146	*x* + 1, *y*, *z*
**2n**					
C16*A*—H16*A*···O4*A*	0.93	2.52	3.3769	154	−*x* + 2, −*y* + 2
C19*A*—H19*A*···O3*A*	0.93	2.59	3.3107	135	−*x* + 1, −*y* + 1, −*z*
**2o**					
N1—H1N1···O2	0.88	2.54	3.0839	120	*x*-1,*y*,*z*
C12—H12B···O2	0.97	2.46	3.4169	171	*x*, −*y* − 1/2, *z* − 3/2
**2p**					
N1—H1N1···O3	0.93	2.07	2.9959	171	*x* + 3/2, −*y*, *z* − 1
**2r**					
C7—H7*A*···O3	0.97	2.59	3.3686	137	*x*, *y* − 1, *z*
C17—H17*A*···O2	0.93	2.52	3.3493	148	−*x* + 2, −*y* + 2, −*z* + 1
C18—H18*A*···O1	0.93	2.59	3.2523	129	*x*, *y* + 1, *z*

[1]*Cg*1 is the centroid of C14—C19 ring.

Table S5. CCDC reference code and its systematic name of reported compounds.

CCDC Reference Code	Systematic Name
AZULUD	2-(4-Chlorophenyl)-2-oxoethyl 4-hydroxybenzoate
CIQNEW	Benzoylmethyl 4-methoxybenzoate
CIXVUC	2-(4-Bromophenyl)-2-oxoethyl 3-chlorobenzoate
CIXWAJ	2-(4-Bromophenyl)-2-oxoethyl 2,4-dichlorobenzoate
CIXWEN	2-(4-Bromophenyl)-2-oxoethyl 2-aminobenzoate
CIXWIR	2-(4-Bromophenyl)-2-oxoethyl 3-aminobenzoate
CIYCAQ	2-(4-Bromophenyl)-2-oxoethyl 2-nitrobenzoate
CIYCEU	2-(4-Bromophenyl)-2-oxoethyl 3-nitrobenzoate
CIYCIY	2-(4-Bromophenyl)-2-oxoethyl 4-nitrobenzoate
CIYCOE	2-(4-Bromophenyl)-2-oxoethyl 4-aminobenzoate
CIYFUN	2-(4-bromophenyl)-2-oxoethyl benzoate
CIYGAU	2-(4-bromophenyl)-2-oxoethyl 2-chlorobenzoate
EVAFOX	2-(4-Bromophenyl)-2-oxoethyl 4-bromobenzoate
EVAJAN	2-(4-Chlorophenyl)-2-oxoethyl 3-(trifluoromethyl)benzoate
EVAJIV	2-(4-Chlorophenyl)-2-oxoethyl 2,4-difluorobenzoate
EVAZEH	2-(4-Chlorophenyl)-2-oxoethyl 2-methoxybenzoate
EVEGIW	2-(4-Bromophenyl)-2-oxoethyl 4-chlorobenzoate
EVEGOC	2-(4-Bromophenyl)-2-oxoethyl 2-methoxybenzoate
EVEVEH	2-(4-Chlorophenyl)-2-oxoethyl benzoate
GARCEJ	2-(4-Fluorophenyl)-2-oxoethyl 2-methoxybenzoate
GITHUN	Benzoylmethyl 4-chlorobenzoate
MANGIR	Phenacyl 5-bromo-2,3,4-trimethylbenzoate
OBOYIP	2-(4-Bromophenyl)-2-oxoethyl 4-methylbenzoate
OCAKUA	2-(4-Chlorophenyl)-2-oxoethyl 3,4-dimethoxybenzoate
OCAQUG	2-(2,4-Dichlorophenyl)-2-oxoethyl 4-methoxybenzoate
OCEFEJ	2-(4-Fluorophenyl)-2-oxoethyl 4-methoxybenzoate
PECZAA	2-(4-Methylphenyl)-2-oxoethyl 3-bromobenzoate
PODQIK	2-(4-chlorophenyl)-2-oxoethyl 3-methylbenzoate
PODRAD	2-(4-chlorophenyl)-2-oxoethyl 3-nitrobenzoate
USIWID	2-Oxo-2-phenylethyl benzoate
USIWOJ	2-(4-Bromophenyl)-2-oxoethyl 4-methoxybenzoate
VOBYUI	2-(4-Chlorophenyl)-2-oxoethyl 2-chlorobenzoate
YAFWEJ	2-(4-Fluorophenyl)-2-oxoethyl 3-(trifluoromethyl)benzoate
YAFZAI	2-(4-Bromophenyl)-2-oxoethyl 4-hydroxybenzoate
YAHGUL	2-(4-Chlorophenyl)-2-oxoethyl 4-methylbenzoate
YAHYOX	2-(4-Bromophenyl)-2-oxoethyl 2-methylbenzoate
